# Safety and pharmacodynamic efficacy of eculizumab in aneurysmal subarachnoid hemorrhage (CLASH): A phase 2a randomized clinical trial

**DOI:** 10.1177/23969873231194123

**Published:** 2023-08-22

**Authors:** Inez Koopman, Reinier WP Tack, Herman F Wunderink, Anke HW Bruns, Irene C van der Schaaf, Daniela Cianci, Kyra A Gelderman, Inge M van de Ridder, Elly M Hol, Gabriel JE Rinkel, Mervyn DI Vergouwen

**Affiliations:** 1 Department of Neurology and Neurosurgery, UMC Utrecht Brain Center, University Medical Center Utrecht, Utrecht University, Utrecht, The Netherlands; 2 Department of Medical Microbiology, University Medical Center Utrecht, Utrecht University, Utrecht, The Netherlands; 3 Department of Internal Medicine and Infectious Diseases, University Medical Center Utrecht, Utrecht University, Utrecht, The Netherlands; 4 Department of Radiology, University Medical Center Utrecht, Utrecht University, Utrecht, The Netherlands; 5 Julius Center for Health Sciences and Primary Care, University Medical Center Utrecht, Utrecht University, Utrecht, The Netherlands; 6 Sanquin Diagnostic Services, Amsterdam, The Netherlands; 7 Department of Intensive Care Medicine, University Medical Center Utrecht, Utrecht University, Utrecht, The Netherlands; 8 Department of Translational Neuroscience, UMC Utrecht Brain Center, University Medical Center Utrecht, Utrecht University, Utrecht, The Netherlands

**Keywords:** Subarachnoid hemorrhage, inflammation, safety and efficacy, complement

## Abstract

**Introduction:**

Complement C5 antibodies reduce brain injury after experimental subarachnoid hemorrhage.

**Patients and methods:**

In this randomized, controlled, open-label, phase 2a clinical trial with blinded-outcome assessment, we included adult aneurysmal subarachnoid hemorrhage (aSAH) patients admitted to a tertiary referral center ⩽11 h after ictus. Patients were randomized (1:1) to eculizumab plus care as usual or to care as usual. Eculizumab (1200 mg) was administered <12 h, and on days 3 and 7 after ictus. In the intervention group, all patients received prophylactic antibiotics and, after a protocol amendment, fluconazole if indicated. Primary outcome was C5a concentration in cerebrospinal fluid (CSF) on day 3 after ictus. Safety was monitored during 4 weeks. In each group, 13 patients with CSF assessments were needed to detect a 55% reduction in CSF C5a concentration.

**Results:**

From October 2018 to May 2021, we enrolled 31 patients of whom 26 with CSF samples, 13 per group. Median C5a concentration in CSF on day 3 was 251 pg/ml [IQR: 103–402] in the intervention group and 371 pg/ml [IQR: 131–534] in the control group (*p* = 0.29). Infections occurred in two patients in the intervention group and four patients in the control group. One patient in the intervention group developed a *C. albicans* meningitis prior to the protocol amendment.

**Discussion and conclusion:**

One dose of eculizumab did not result in a ⩾ 55% decrease in C5a concentration in CSF on day 3 after aSAH. The study did not reveal new safety concerns, except for a *C. albicans* drain-related infection prior to antifungal monitoring and treatment.

**Trial registration:**

EudraCT 2017-004307-51, https://www.clinicaltrialsregister.eu/

## Introduction

In patients with aneurysmal subarachnoid hemorrhage (aSAH), important determinants of poor functional outcome are the extent of early brain injury and delayed cerebral ischemia.^1^ No treatment exists to reduce early brain injury, and the effects of current strategies to prevent delayed cerebral ischemia are modest.^2^ Neuroinflammation may contribute to early brain injury and delayed cerebral ischemia.^3^ Activation of the complement system, an important driver of neuroinflammation, has been associated with poor functional outcome in aSAH patients.^4,5^ In experimental SAH, C5-specific antibodies, which prevent the formation of the pro-inflammatory peptide C5a and the membrane attack complex, reduced microglial activation and cell death by 40%.^6^ We aimed to investigate the safety and pharmacodynamic efficacy (proof-of-concept) of eculizumab administration on C5a levels in the cerebrospinal fluid (CSF) of aSAH patients.

## Methods

### Study design

The CompLement C5 Antibodies for decreasing brain injury after aneurysmal Subarachnoid Hemorrhage (CLASH) study is an investigator-initiated randomized, open-label phase 2a trial with blinded-outcome assessment in a tertiary referral center in the Netherlands. The study protocol has been published elsewhere.^7^ The research ethics committee of the University Medical Centre Utrecht, The Netherlands, approved the study (17/933). Patients and/or their legal representatives provided written informed consent in accordance with the Declaration of Helsinki.

### Participants

Between October 2018 and March 2019, adult aSAH patients were eligible for inclusion if they were admitted to the University Medical Center Utrecht ⩽6 h after ictus and had a Glasgow Coma Scale (GCS) score of 14 or less. Because of slow recruitment, we amended our in- and exclusion criteria per March 2019 to include aSAH patients if they were admitted to the University Medical Center Utrecht ⩽11 h after ictus and we no longer excluded patients with a GCS score of 15 on admission. An overview of all in- and exclusion criteria can be found in the Supplemental Material 1 Table S1. The physician on call identified potential participants. Patients or legal representatives were asked for written informed consent by the physician on call or by the research team. If the patient was incapacitated, the patient was asked for consent as soon as the patient regained capacity, even if a legal representative had already provided consent.

### Randomization and masking

Patients were randomly assigned in a 1:1 ratio to either eculizumab plus care as usual (intervention group) or care as usual alone (control group). Randomization occurred by a centralized secured website based on a computer-generated block randomization scheme (block size of one or two). No stratification factors were used. Patients and/or their legal representatives, treating physicians, and the research team were informed about the treatment allocation. Research team members who assessed the primary outcome and serological parameters (lab technicians), modified Rankin Scale (mRS) score (research nurse), and infections (HFW, AHWB) were masked to treatment allocation.

### Procedures

Patients allocated to eculizumab treatment, received intravenous infusion with eculizumab 1200 mg at three time points: <12 h, on day 3, and day 7 after ictus. Eculizumab infusions were prepared according to the instructions of the manufacturer.^8^ Day of ictus was defined as day 1. As this was the first trial to investigate the use of eculizumab in aSAH patients, the effective dosing regimen for aSAH patients was unknown. In our previous study, we found that the CSF C5a concentration in aSAH patients was increased >1400 times compared to the CSF C5a concentration in patients with unruptured intracranial aneurysms.^6^ We therefore decided to administer a high dose of eculizumab (1200 mg) with repeated drug administration to prevent a wash-out effect. Patients in the intervention group received prophylactic treatment with antibiotics covering meningococci for the first 4 weeks after ictus, because of an increased risk of meningococcal infections in patients without adequate functional complement due to eculizumab treatment. During the recruitment phase, after the inclusion of the sixth patient, we changed our protocol based on a serious adverse event (SAE) that occurred (*Candida albicans* drain-related meningitis in a patient with an external ventricular CSF drain). After the amendment, patients in the intervention group received targeted prophylactic antibiotics for the first 4 weeks after ictus and fluconazole if indicated by a higher risk of infection based on surveillance cultures. Throat and rectal swabs were performed weekly in the intervention group during hospital admission to test for carriership/colonization with yeasts and (multi-) drug resistant bacteria. In case of a central line or an external ventricular CSF drain in combination with a positive yeast culture, prophylactic fluconazole was added for the first 4 weeks after ictus. In consultation with the microbiologist and infectious disease specialist, we changed prophylactic treatment if swabs were positive for microorganisms that required treatment and were not covered by our prophylactic regimen. All patients received care as usual according to our local treatment protocol (Supplemental Material 1).

Study procedures included daily Glasgow Coma Scale evaluations; serum withdrawal on multiple days (day 1 (before the first eculizumab administration), day 2, day 4, day 6, day 9, day 12, and day 14); CSF withdrawal on day 3 (before the second eculizumab administration), National Institutes of Health Stroke Scale (NIHSS), and World Federation of Neurosurgical Societies (WFNS) scores and a brain magnetic resonance imaging (MRI) scan 2 weeks after ictus; (serious) adverse event ((S)AE) recording up to 4 weeks after ictus, and a(n) (S)AE questionnaire 4 weeks after ictus; Montreal Cognitive Assessment (MoCA) and quality of life assessment (EQ-5D-5L) in the outpatient clinic 10 weeks after ictus; and a mRS score 13 weeks after ictus. CSF was obtained by either lumbar puncture or sampling from an external lumbar or ventricular CSF drain. Serum and CSF sampling, processing, and storage are described in the Supplemental Material 1. Brain MRI scans were examined for cerebral ischemia by a neuroradiologist masked for treatment allocation. AEs, SAEs, and Suspected Unexpected Serious Adverse Reactions (SUSARs) were collected by patient or legal representative self-report and by review of the electronic health records up to 4 weeks after ictus. Safety was further examined by the data and safety monitoring board (DSMB) with ongoing monitoring of SAEs, infections, and an interim analysis based on SAE reporting, clinical outcome, and case-fatality. Blinded assessment of possible infections was performed by an expert panel consisting of a microbiologist and an infectious disease specialist and based on anonymized clinical, laboratory, and radiological data. In case of disagreement between the members of the expert panel, a consensus meeting was held. A dedicated research nurse blinded to treatment allocation determined the mRS score by telephone interview. All data were recorded in an electronic data capture system (ResearchOnline), which was integrated with the randomization system. A contract research organization performed multiple audit trials according to a monitor plan approved by the research ethics committee, which included verification of source data.

### Outcomes

The primary outcome was C5a concentration in CSF on day 3 after ictus. We chose C5a as the primary outcome instead of free C5 because the primary target of this study was the anaphylatoxin C5a and not the membrane attack complex (C5b-9). Secondary outcomes were safety until 4 weeks after ictus, inflammatory parameters and eculizumab concentration in serum on day 1, 2, 4, 6, 9, 12, and 14 and CSF on day 3 after ictus, clinical condition measured by the NIHSS and WFNS score and any cerebral infarction on MRI at 2 weeks after ictus, cognition (MoCA) and quality of life scores (EQ-5D-5L/ EQ-VAS) 10 weeks after ictus, and functional outcome as measured by the mRS score 13 weeks after ictus. Serum and CSF immunoassays are described the Supplemental Material 1.

### Statistical analysis

Our sample size was based on the findings of a previous study with eculizumab in patients with neuromyelitis optica.^9^ In that study, free C5 concentration in CSF was measured in 11 patients before and after treatment with eculizumab. In six patients, free C5 was undetectable after treatment was started, and in the remaining five patients, the mean free C5 concentration in CSF decreased with 58%. For the current trial, we assumed an overall reduction in free C5 concentration in CSF with 55% between the intervention and control group and extrapolated this to a similar reduction in C5a concentration in CSF. A sample size of 13 patients with CSF samples in each group was estimated to provide 80% power with a significance level of 5% to detect an overall 55% difference in C5a concentration. The analyses and reporting of this study were performed according to a predefined statistical analysis plan (Supplemental Material 2). Descriptive statistics were used to summarize data. We defined two analysis sets: the per-protocol set and the intention-to-treat set. In the per-protocol set, patients with CSF assessments were included if they had received the first dose of eculizumab and completed the study according to protocol up to and including CSF withdrawal on day 3 after ictus. In the intention-to-treat set, patients were included according to the treatment allocation, irrespective of whether or not they had received the first dose of eculizumab. The per-protocol set was used in the primary analysis. For the secondary analyses, both the per-protocol and intention-to-treat sets were used. We did not define a safety population due to the open label design of this trial. Normality of data was explored by a *Q*-*Q* plot. A Mann-Whitney *U*-test was used to detect a statistical difference between the CSF C5a concentration in the intervention and control group. We performed a sensitivity analysis for the primary outcome with the Prognosis on Admission of Aneurysmal Subarachnoid Hemorrhage Scale (PAASH) score and Hijdra sum score as covariates in an analysis of covariance using a rank transformation. If the patient was intubated and sedated on admission to the tertiary referral center, we used the PAASH score based on the GCS score before intubation for the sensitivity analysis. Safety analysis was based on all participants and presented as tabulated incidence rates. Statistical differences in the concentration of CSF and serum parameters and clinical outcomes between both groups were assessed with an independent *t*-test or Mann-Whitney *U*-test depending on the distribution of the data. If the concentration of CSF or serum parameters was below detection limit, we used the value of the detection limit, unless half or more patients who reached the outcome had a value below detection limit, in which case we did not report mean or median values and did not perform statistical testing. If the lower limit of quantification (LLOQ) was below the detection limit, the LLOQ was used. We made changes to the secondary analyses in the statistical analysis plan (SAP) compared to the protocol paper, because some analyses could not be performed (Supplemental Material 2). A post-hoc analysis was done to investigate CSF C5a concentration by sample type (lumbar puncture or external ventricular drain). IBM SPSS version 26 was used for all analyses.

## Results

**Figure 1.**
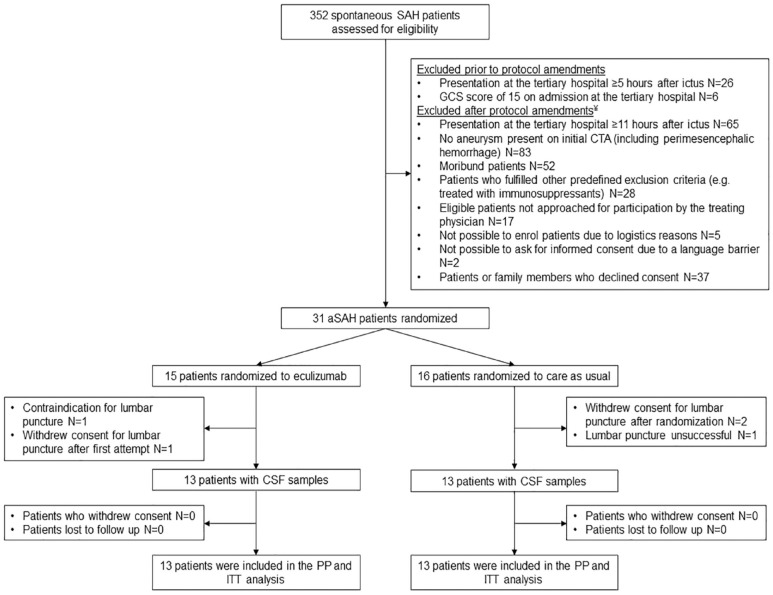
Trial profile. (a)SAH: (aneurysmal) subarachnoid hemorrhage; GCS: Glasgow Coma Scale; CTA: computed tomography angiography; CSF: cerebrospinal fluid; ITT: intention-to-treat; PP: per-protocol. ^¥^To expand eligibility criteria.

Between October 5, 2018 and May 27, 2021, 352 patients with spontaneous subarachnoid hemorrhage were screened. Out of 92 patients who were eligible for study enrollment (Figure 1), 31 patients were randomly assigned (15 to eculizumab and 16 to care as usual). Recruitment was halted after CSF samples were obtained in 26 patients according to the power calculation in the study protocol (13 patients in the intervention group and 13 patients in the control group). All 26 patients with CSF samples were included in the per-protocol and intention-to-treat analysis for the primary outcome. No patients were lost to follow-up. Patient characteristics were similar between both groups (Table 1). Patients were predominantly women and had a mean age of 59 years (SD 11). Median time from ictus to randomization was 5:34 h [IQR: 4:59–6:32] for patients in the intervention group and 5:27 h [IQR: 4:05–8:30] for patients in the control group.

**Table 1.** Patient characteristics.

	Eculizumab	Care as usual
	*N* = 15 (%)	*N* = 16 (%)
Female sex	11 (73)	13 (81)
Mean age [SD]	59 [7]	60 [14]
History of hypertension	6 (40)	4 (25)
Smoking status		
Current smoker	6 (40)	5 (31)
Past smoker	7 (47)	2 (13)
Never smoker	2 (13)	9 (56)
mRS score before SAH occurred		
0	13 (87)	15 (94)
1	2 (13)	1 (6)
PAASH score on admission to tertiary center		
1 (GCS 15)	4 (27)	5 (31)
2 (GCS 11–14)	6 (40)	5 (31)
3 (GCS 8–10)	2 (13)	1 (6)
4 (GCS 4–7)	1 (7)	1 (6)
5 (GCS 3)	0 (0)	0 (0)
Patient intubated and sedated	2 (13)	4 (25)
Loss of consciousness at onset	8 (53)	10 (63)
Median Hijdra sum score [IQR] on admission^a^	26 [24–27]	26 [22–33]
Anterior circulation aneurysm	12 (80)	12 (75)
Median size of the ruptured aneurysm in mm [IQR]	7 [4–8]	6 [4–9]
Aneurysm treatment modality		
Endovascular treatment	8 (53)	10 (63)
Neurosurgical clipping	7 (47)	6 (38)
Median time from ictus to randomization, h:min	5:34 [4:59–6:32]	5:27 [4:05-8:30]
Mean time from admission to tertiary center to treatment of the aneurysm, h:min	21:09 (15:43-26:34)	18:05 (14:40-21:29)
CSF withdrawal	13 (87)	13 (81)
Lumbar puncture	5 (38)	8 (62)
EVD	8 (62)	5 (38)

SD: standard deviation; mRS: modified Rankin Scale; SAH: subarachnoid hemorrhage; PAASH: Prognosis on Admission of Aneurysmal Subarachnoid Hemorrhage; GCS: Glasgow Coma Scale; IQR: interquartile range; CSF: cerebrospinal fluid; EVD: external ventricular drain.

a Hijdra sum score was determined on the head computed tomography (CT) scan performed on admission to the tertiary center unless no new head CT scan was made, in which case the head CT scan of the referring center was used.

### Pharmacodynamic efficacy

**Figure 2.**
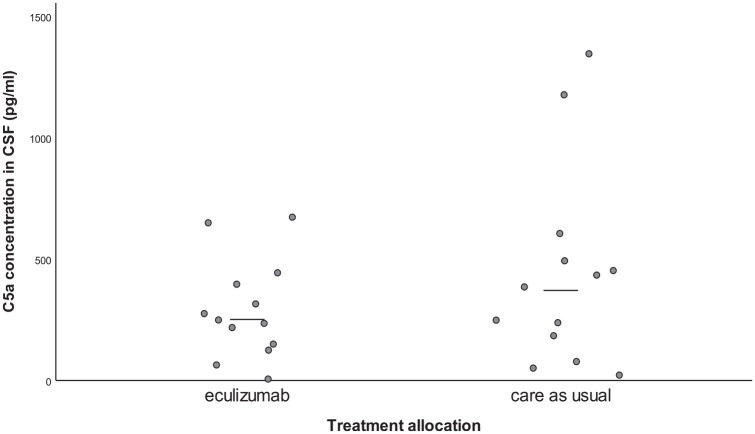
C5a concentration in CSF on day 3. CSF: cerebrospinal fluid. Points in the graph represent individual patient data. The horizontal line represents the median concentration in each group.

Median C5a concentration in CSF on day 3 was 251 pg/ml [IQR: 103–402] in the intervention group and 371 pg/ml [IQR: 131–534] in the control group (*p* = 0.29, Table 2 and Figure 2). Results of the primary analysis remained similar in a sensitivity analysis with the PAASH and Hijdra sum score on admission as covariates (*p* = 0.22).

**Table 2.** Primary and secondary outcomes.

	*N*	Eculizumab	*N*	Care as usual	*p*-Value
*Primary outcome*					
C5a concentration in CSF pg/ml [IQR]	13	251 [103–402]	13	371 [131–534]	0.29^a^
*Secondary clinical outcomes*					
NIHSS score at 9–15 days [IQR]	14	2 [0–5]	16	0.5 [0–6]	0.79
WFNS score day at 9–15 days [IQR]	14	2 [1–2]	15	1 [1–2]	0.18
Cerebral infarction on brain MRI at 9–31 days (%)	11	9 (69)	10	5 (38)	0.24
MoCA score at 7–13 weeks [IQR]	12	26 [21–27]	12	26 [23–28]	0.41
EQ-5D-5L at 7–13 weeks [IQR]	12	0.82 [0.78–0.91]	13	0.87 [0.77–1.00]	0.29
EQ-VAS at 7–13 weeks [95% CI]	12	72 [64–79]	12	80 [71–88]	0.14
mRS score at 10–15 weeks [IQR]	15	2 [2–4]	16	2 [2–3]	0.55
1		1 (7)		3 (19)	
2		9 (60)		8 (50)	
3		1 (7)		2 (13)	
4		1 (7)		1 (6)	
5		1 (7)		0 (0)	
6		2 (13)		2 (13)	

IQR: interquartile range; NIHSS: National Institutes of Health Stroke Scale; WFNS: World Federation of Neurosurgical Societies; Cerebral infarction: any region of diffusion restriction on diffusion-weighted imaging (MRI); MoCA: Montreal Cognitive Assessment; EQ-5D-5L and EQ-VAS: quality of life assessments; mRS: modified Rankin Scale.

a In both the per-protocol and intention-to-treat analysis.

### Safety

Frequently reported (S)AEs were symptoms and complications which often occur in aSAH patients (Table 3). The number of patients with (S)AEs was similar in both groups. Infections occurred in two patients (13%) in the intervention group and in four patients (25%) in the control group (Table 3 and Supplemental Material 1 Table S2). One patient in the intervention group with an external ventricular CSF drain developed a *C. albicans* drain-related meningitis prior to implementation of a protocol amendment, which included monitoring of carriership/colonization with yeasts and treatment with fluconazole if indicated. This patient, while recovering from the drain-related meningitis with antifungal treatment, had rebleeding from a partially coiled large aneurysm twice after SAE follow-up time and died. Two other patients, one in each group, died within SAE follow-up time. The patient in the intervention group died from a massive pulmonary embolism 10 days after hospital admission. The pulmonary embolism was reported as a SUSAR to the ethics committee. Pulmonary embolism or deep vein thrombosis are not reported as side effects of eculizumab in the investigators brochure, in previous trials, or in case series. Moreover, mechanistically, one would expect that eculizumab would reduce the risk of thrombosis. The DSMB and investigators therefore considered the pulmonary embolism to be unrelated to eculizumab. The patient in the control group developed multiple complications during in-hospital stay including an aspiration pneumonia with subsequent respiratory failure, methicillin-resistant *Staphylococcus aureus* sepsis, cardiomyopathy, and ICU-acquired weakness after which the decision was made to withdraw care. In none of the patients, eculizumab dosing was reduced or stopped. This included patients with a drain – related meningitis because these infections developed after the last administration of eculizumab. No infusion reactions or episodes of anaphylaxis were reported.

**Table 3.** (S)AEs after trial enrollment up until 4 weeks after ictus.

	Eculizumab *N* = 15 (%, 95% CI)^a^	Care as usual *N* = 16 (%, 95% CI)^a^
Any AE	15 (100)	16 (100)
Most frequently reported AEs in either group^b^		
Hypokalemia	14 (93, 68–100)	14 (88, 62–98)
Nausea and/or vomiting	11 (73, 45–92)	10 (63, 35–85)
Hypertension	8 (53, 27–79)	7 (44, 20–70)
Fatigue	8 (53, 27–79)	6 (38, 15–65)
Hyponatremia	6 (40, 16–68)	10 (63, 35–85)
Any SAE	11 (73, 45–92)	7 (44, 20–70)
Most frequently reported SAEs in either group		
Cerebral ischemia (post-procedural or related to delayed cerebral ischemia)	7 (47, 21–73)	6 (38, 15–65)
Hydrocephalus which required treatment	4 (27, 8–55)	3 (19, 4–46)
Rebleeding before or during treatment	3 (20, 4–48)	1 (6), 0–30
Drain-related meningitis	2 (13, 2–40)	3 (19, 4–46)
SAEs possibly or probably related to eculizumab as determined by the investigators and an expert group		
Fungal meningitis, drain-related	1 (7, 0–32)	0 (0, 0–17)
Death	1 (7, 0–32)	1 (6, 0–30)
Infections during (S)AE follow-up as determined by an expert panel according to the CDC criteria^28,c^	2 (13, 2–40)	4 (25, 7–52)
Meningitis, drain-related	2 (13, 2–40)	2 (13, 2–38)
Bacteremia	0 (0, 0–18)	2 (13, 2–38)
Pneumonia	0 (0, 0–18)	2 (13, 2–38)
Upper respiratory tract infection	0 (0, 0–18)	1 (6, 0–30)
Any SUSAR	1 (7, 0–32)	0 (0, 0–17)

(S)AEs: (serious) adverse events; CI: confidence interval; DCI: delayed cerebral ischemia; CDC: Centers for Disease Control and Prevention; SUSAR: suspected unexpected serious adverse reaction.

a Percentage of patient with (S)AEs and the 95% confidence interval.

b Headache and neck pain were not included in the frequently reported AE list because these are primary symptoms of SAH.

c Pathogens can be found in the Supplemental Material 1 Table S2.

### Secondary outcome measures

**Figure 3.**
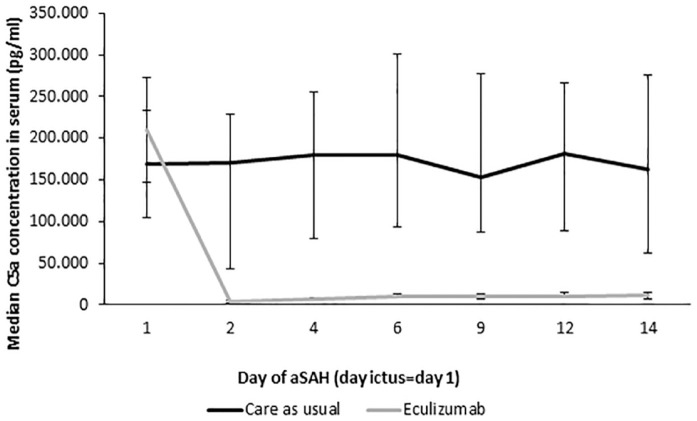
Median C5a concentration in serum. aSAH: aneurysmal subarachnoid hemorrhage; CI: confidence interval; IQR: interquartile range. On day 1 the mean value with 95% CI is depicted. The other values are medians with IQRs. Error bars represent 95% CI (on day 1) or IQR values (25th–75th percentiles).

Eculizumab concentration in CSF on day 3 varied substantially with a median concentration of 0.14 ug/ml [IQR: 0.10–0.34] (Supplemental Material 1 Figure S1). Inflammatory parameters in CSF did not differ between the intervention and control group (Supplemental Material 1 Table S3 and Figures S2–S12). Eculizumab concentration in serum ranged from 143 to 844 μg/ml (Supplemental Material 1 Figure S13). Eculizumab decreased C5a concentration in serum (Supplemental Material 1 Table S4 and Figure 3) and functional complement activity of the classical, alternative, and lectin pathways of complement activation (Supplemental Material 1 Tables S5–S7 and Figures S14–S16). Overall, interleukin-6, interleukin-10, sC5b-9, and C-reactive protein concentration in serum did not differ between both groups (Supplemental Material 1 Tables S8–S11 and Figures S17–S19). Clinical, radiological, and cognitive outcomes were similar between both groups (Table 2 and Supplemental Material 1 Figures S20–S22).

### Post hoc analysis

Mean C5a concentration in CSF samples withdrawn by lumbar puncture on day 3 was 276 pg/ml (95% CI: 90–462) in the intervention group and 210 pg/ml (95% CI: 96–325) in the control group (*p* = 0.43) (Supplemental Material 1 Table S12). Mean C5a concentration in CSF samples from an external ventricular drain on day 3 was 262 (95% CI: 63–462) in the intervention group and 798 (95% CI: 253–1342) in the control group, a decrease in C5a concentration of 67% (*p* < 0.05) (Supplemental Material 1 Table S12). The median Hijdra sum score was 24 [21–27] in patients with lumbar CSF samples and 32 [26–34] in patients with CSF samples from an external ventricular drain (*p* ⩽ 0.01) (Supplemental Material 1 Table S13).

## Discussion

In this study, we found no evidence of a ⩾55% decrease in CSF C5a concentration in aSAH patients on day 3 after the first dose of eculizumab. However, with our high dose eculizumab administration thrice in the first week after aSAH onset, we found a profound reduction in serum C5a concentration and complement activity, which confirms the pharmacological efficacy of this regimen in serum. We found no increase in infection rate with this eculizumab dosing regimen in combination with infection monitoring by performing surveillance cultures and a prophylactic regimen. There were no other safety concerns, except for a fungal drain-related meningitis in one patient prior to antifungal monitoring and treatment.

Eculizumab, a humanized monoclonal antibody directed against complement component C5, was first approved as treatment for paroxysmal nocturnal hemoglobinuria and atypical hemolytic-uremic syndrome, because it reduced complement-mediated intravascular hemolysis and thrombotic microangiopathy.^10,11^ Subsequent studies showed eculizumab also improved symptoms in complement-mediated neurological diseases, such as neuromyelitis optica spectrum disorders and refractory generalized myasthenia gravis.^9,12–14^ In aSAH, early brain injury and delayed cerebral ischemia may also be complement-mediated. Subarachnoid blood followed by the degradation of red blood cells, endothelial damage, and cerebral hypoperfusion initiate a neuroinflammatory response.^15,16^ Subsequent cell death and inflammation-induced vasoconstriction may contribute to early brain injury and delayed cerebral ischemia.^15,16^

The CLASH trial is the first in-human study on short-term treatment with eculizumab in an acute disease. Therefore, we based the high dose eculizumab and repeated drug administration on the results of a previous study, in which we found that the CSF C5a concentration in aSAH patients is increased >1400 times on day 1 after ictus compared to the CSF C5a concentration in control patients and slowly decreased until day 10 after ictus.^6^ Because SAH is an acute disease, it was not possible to vaccinate patients against meningococcal disease 2 weeks in advance, as is recommended in patients with a chronic disease treated with eculizumab.^8^

CSF eculizumab concentration varied substantially between patients after one dose of eculizumab, possibly due to differences in blood-CSF barrier disturbances among aSAH patients and thereby eculizumab penetration. In patients with neuromyelitis optica, treatment with eculizumab resulted in a decrease in CSF free C5 concentration of more than 55%, while CSF eculizumab concentrations were lower than in our study.^9^ One possible explanation why such a decrease in C5a concentration was not reached in our study is because other enzymes, such as thrombin and trypsin, have been found to generate biologically active C5a despite the presence of eculizumab.^17^ Since CSF thrombin levels are increased in aSAH patients,^18^ inhibition of C5 cleavage to C5a by eculizumab might be insufficient. However, the results of the post hoc analysis contradict this hypothesis, since the post-hoc analysis showed that in CSF samples from an external ventricular drain a >55% CSF C5a reduction was achieved. aSAH patients with a high burden of subarachnoid hemorrhage were found to have the highest thrombin levels.^18^ We would expect that patients with CSF samples from a ventricular drain would have an increased amount of subarachnoid blood on the initial scan compared to patients with lumbar CSF samples, which in turn would result in increased C5a levels in CSF and thus higher levels of thrombin and C5a in the eculizumab group. Instead, a stronger reduction in C5a levels with eculizumab treatment was found in patients with CSF samples from an external ventricular drain compared patients with lumbar CSF samples.

Various neuroinflammatory parameters, such as complement components, interleukin-6, and TNF-a have been associated with poor functional outcome after SAH and are therefore a target for intervention.^4,5,19,20^ Two non-randomized studies investigating the effect of the serine protease inhibitor FUT-175 (nafamostat mesilate), an inhibitor of the complement system, showed that patients treated with FUT-175 had a lower risk of delayed cerebral ischemia and a trend toward better functional outcomes compared to historical controls.^21,22^ In a randomized clinical trial, treatment of SAH patients with interleukin-1 receptor antagonists resulted in decreased peripheral inflammation, but the reduction in inflammatory CSF parameters did not reach statistical significance, possibly because of a small sample size.^23,24^ These results are in line with our findings, in which complement activity is highly reduced in serum, but a similar effect is not found in CSF.

Eculizumab can increase susceptibility to fungal infections.^8,25^ A relation between eculizumab and the *C. albicans* drain-related meningitis which occurred in our study was considered “possible or probable” by the investigators and an expert group (microbiologists and an infectious disease specialist). Nevertheless, a fungal drain-related meningitis can also occur in aSAH patients with an external ventricular drain who do not receive eculizumab.^26^ No other cases of fungal drain-related meningitis were reported during the remainder of the study after amendment of the study protocol to include yeast carriership/colonization monitoring and treatment with fluconazole if indicated.

SAH patients are often treated with anti-inflammatory drugs such as aspirin or steroids. Aspirin or other types of platelet aggregation inhibitors are often used after coiling or placement of a flow-diverting stent, while steroids may be given (in the absence of evidence) for reducing cerebral edema after SAH. No contra-indication exists for treatment with eculizumab in patients already treated with aspirin, other platelet aggregation inhibitors or steroids.^8^

Several limitations need to be mentioned. First, CSF samples were obtained either by lumbar puncture or from an external ventricular CSF drain. In the post hoc analysis, C5a concentration in lumbar CSF samples and in CSF samples from an external ventricular drain differed between the intervention and control group. Future studies should preferably use only one method of CSF sampling. Second, sampling, processing and storage conditions can influence C5a measurements.^27^ To minimize variability, we used standard operating procedures for sample handling. Third, the variation in CSF C5a concentration was higher than expected, making it difficult to detect a large difference between groups in a small sample. The study that detected a 55% difference used a pre-test and post-test design and was therefore less affected by between-patient variability.^9^ The high between-patient variation in C5a concentration should be taken into consideration in future studies, either by using a pre-test and post-test design or by performing a study in a larger sample.

In conclusion, treatment with eculizumab did not decrease CSF C5a concentration with ⩾55% on day 3 after one dose of eculizumab. Safety findings showed no other safety concerns than in previous studies, except for a *C. albicans* drain-related infection prior to antifungal monitoring. Since the results of this study were inconclusive about a C5a reduction smaller than 55%, a phase 2b study in a larger population is needed to investigate a potential smaller pharmacodynamic effect of eculizumab in aSAH patients and the clinical effects of such a smaller pharmacodynamic effect.

## Supplemental Material

sj-docx-1-eso-10.1177_23969873231194123 – Supplemental material for Safety and pharmacodynamic efficacy of eculizumab in aneurysmal subarachnoid hemorrhage (CLASH): A phase 2a randomized clinical trial Click here for additional data file. Supplemental material, sj-docx-1-eso-10.1177_23969873231194123 for Safety and pharmacodynamic efficacy of eculizumab in aneurysmal subarachnoid hemorrhage (CLASH): A phase 2a randomized clinical trial by Inez Koopman, Reinier WP Tack, Herman F Wunderink, Anke HW Bruns, Irene C van der Schaaf, Daniela Cianci, Kyra A Gelderman, Inge M van de Ridder, Elly M Hol, Gabriel JE Rinkel and Mervyn DI Vergouwen in European Stroke Journal

sj-docx-2-eso-10.1177_23969873231194123 – Supplemental material for Safety and pharmacodynamic efficacy of eculizumab in aneurysmal subarachnoid hemorrhage (CLASH): A phase 2a randomized clinical trial Click here for additional data file. Supplemental material, sj-docx-2-eso-10.1177_23969873231194123 for Safety and pharmacodynamic efficacy of eculizumab in aneurysmal subarachnoid hemorrhage (CLASH): A phase 2a randomized clinical trial by Inez Koopman, Reinier WP Tack, Herman F Wunderink, Anke HW Bruns, Irene C van der Schaaf, Daniela Cianci, Kyra A Gelderman, Inge M van de Ridder, Elly M Hol, Gabriel JE Rinkel and Mervyn DI Vergouwen in European Stroke Journal

sj-pdf-3-eso-10.1177_23969873231194123 – Supplemental material for Safety and pharmacodynamic efficacy of eculizumab in aneurysmal subarachnoid hemorrhage (CLASH): A phase 2a randomized clinical trial Click here for additional data file. Supplemental material, sj-pdf-3-eso-10.1177_23969873231194123 for Safety and pharmacodynamic efficacy of eculizumab in aneurysmal subarachnoid hemorrhage (CLASH): A phase 2a randomized clinical trial by Inez Koopman, Reinier WP Tack, Herman F Wunderink, Anke HW Bruns, Irene C van der Schaaf, Daniela Cianci, Kyra A Gelderman, Inge M van de Ridder, Elly M Hol, Gabriel JE Rinkel and Mervyn DI Vergouwen in European Stroke Journal
