# Supplementary material for: Safety and pharmacodynamic efficacy of eculizumab in aneurysmal subarachnoid hemorrhage (CLASH): A phase 2a randomized clinical trial
Source: Eur Stroke J. 2023 Aug 22;8(4):1097–106. doi: 10.1177/23969873231194123 (PMC10683736; doi:10.1177/23969873231194123)
Supplement: sj-docx-1-eso-10.1177_23969873231194123 – Supplemental material for Safety and pharmacodynamic efficacy of eculizumab in aneurysmal subarachnoid hemorrhage (CLASH): A phase 2a randomized clinical trial [file sj-docx-1-eso-10.1177_23969873231194123.docx]

**Supplementary material 1**

**Safety and pharmacodynamic efficacy of eculizumab in aneurysmal subarachnoid hemorrhage** **(CLASH): a phase 2a randomized clinical trial**

Inez Koopman, MD;^1^ Reinier W.P. Tack, MD;^1^ Herman F. Wunderink, MD;^2^ Anke H.W. Bruns, MD;^3^ Irene C. van der Schaaf, MD;^4^ Daniela Cianci, PhD;^5^ Kyra A. Gelderman, PhD;^6^ Inge M. van de Ridder, MD;^7^ Elly M. Hol, PhD;^8^ Gabriel J.E. Rinkel, MD;^1^ Mervyn D.I. Vergouwen, MD^1^

**Table of contents**

Table S1. Inclusion and exclusion criteria p. 2

Local treatment protocol for aSAH patients p. 3

Sampling, processing, storage, and immunoassays serum and CSF samples p. 4

Table S2. Infections and their pathogens p. 5

Figure S1. Eculizumab concentration in CSF on day 3 p. 6

Table S3. Secondary CSF outcomes p. 7

Figures S2-S12. Secondary CSF outcomes p. 8-13

Figure S13. Eculizumab concentration in serum p. 14

Table S4. C5a concentration in serum p. 15

Tables S5-S7. Median complement pathway activity in serum p. 16

Figures S14-16. Median complement pathway activity in serum p.17-18

Tables S8-S11. Serum outcomes p. 19-20

Figures S17-S19. Serum outcomes p. 21-22

Figures S19-S21. GCS score per day p. 23

Figure S22. mRS score according to treatment allocation p. 24

Table S12. C5a concentration in CSF by sample type p. 25

Table S13. PAASH and Hijdra sum score by sample type p. 25

References p. 26

**Table S1. Inclusion and exclusion criteria**

| Inclusion criteria:  • Aneurysmal subarachnoid hemorrhage  • Admission to the University Medical Centre Utrecht ≤11 hours after ictus  • Age of 18 years or older  Exclusion criteria:  • Life expectancy < 10 days  • Pregnant or breastfeeding women  • Participation in another clinical therapeutic study  • History of splenectomy or asplenia  • Hematological malignancy  • Patients receiving chemotherapy  • Patients who will undergo or underwent an organ transplantation  • Patients with myasthenia gravis, glucose-6-phosphate dehydrogenase  (G6PD) deficiency, or tuberculosis  • Patients who are or will be treated by plasmapheresis or hemodialysis  • Patient with a creatinine clearance of < 30 or serum creatinine levels of > 169 μmol/l  • Patients with a known hereditary complement deficiency  • Patients allergic to eculizumab, proteins derived from mouse products, or other monoclonal antibodies  • Patients allergic to (prophylactic) antibiotic treatment for Neisseria meningitidis (quinolones or ceftriaxone)  • If on admission, it is likely that the aneurysm can only be treated with extracranial-intracranial bypass surgery  • If based on head imaging, it will be unlikely that CSF can be obtained at day 3 after ictus  • Patients with an ongoing infection on admission which is not appropriately treated  • Patients who were treated > 4 times with antibiotics during the last year  • Patients on immunosuppressive therapy |
| --- |

CSF= cerebrospinal fluid.

**Local treatment protocol for aSAH patients**In our hospital, aSAH patients are admitted to an intensive care unit (ICU) if the patient has a Glasgow Coma Scale (GCS) score of 8 or lower on admission with a need of mechanical ventilation. All other aSAH patients are admitted to a medium care unit (MCU). Aneurysm treatment occurs ≤ 72 hours after admission according to the ESO guidelines.^1^ If the ruptured aneurysm is treated by neurosurgical clipping, the patient is observed afterwards at the ICU for at least one day. Patients receive nimodipine 60 mg every 4 hours orally or via nasogastric tube. In addition to treatment with nimodipine, we aim for normovolemia (fluid intake 2.5-3L) with saline fluids (sodium chloride 0.9%). Patients on the ICU receive selective decontamination of the digestive tract (SDD) if the expected stay at the ICU is ≥48 hours. SDD is a prophylactic antibiotic regimen, that consist of topical antibiotics applied to the oropharynx and the intestinal tract to prevent colonization of Gram-negative bacteria, Staphylococcus aureus, and yeasts. These topical antibiotics contain polymyxine, tobramycine and nystatine in combination with intravenous administration of ceftriaxone during the first 4 days in ICU. No SDD is given at the neurology and neurosurgery medium care units or wards. Antihypertensive medication is halted on admission. Blood pressure is kept below ≤180 mmHg before aneurysm treatment. If the aneurysm is secured, hypertension (>180 mmHg in patients with an unsecured aneurysm and >220 mmHg or a MAP >130 in patients with a secured aneurysm) is treated with analgesic and/or antihypertensive drugs at the ICU. At the MCU, the use of antihypertensive drugs is avoided as much as possible and is only administered according to the treating physician’s opinion.

**Sampling, processing, storage, and immunoassays** **serum and CSF samples**

*Blood sampling, processing and storage*

Blood from participants was collected in lithium heparin tubes for C-reactive protein (CRP) measurements and serum tubes for cytokine levels and functional activity of complement pathways. Upon blood draw, the two serum collection tubes were inverted five times. One of the serum collection tubes was placed on ice with the exception of the tube drawn on day 1 because logistically this was not possible to arrange in the emergency setting. All samples were immediately transferred to the clinical laboratory of the hospital. The lithium heparin samples were spun at room temperature 1861g for 10 minutes. The supernatant was used to measure CRP levels. The serum collection tubes were allowed to stand for 30 minutes after receipt at the clinical laboratory and were then spun at 4 °C 2000g for 10 minutes. The supernatant was collected, aliquoted, and frozen in a –80°C freezer, where it was stored until analysis.

*CSF sampling, processing and storage*

CSF from participants was collected in polystyrene collection tubes. The sample tube was immediately placed on ice and transferred to the clinical laboratory where samples were spun at 4 °C 2000g for 10 minutes. The supernatant was collected, aliquoted, and frozen in a –80°C freezer, where it was stored until analysis.

*Immunoassays*

To minimize intra-assay variation, the multiplex immunoassays in serum and CSF were measured in one batch. Other assays were performed in multiple batches, since analysis in one batch was not possible.

*Multiplex immunoassays*

C5a, IL-1B, IL-6, and Il-10 in serum and C5a, IL-1B, IL-6, IL-10, IL-18, MCP-1, sICAM, sVCAM, sCD163, P-selectin, E-selectin, TNF-a, and MIF levels in CSF were measured by Multiplex immunoassays at the Multiplex Core facility of the Center of Translational Immunology (UMC Utrecht, The Netherlands), as described previously.^2^ The limit of detection was defined as the lowest analyte concentration that can reliably be distinguished from the blank value without consideration of precision and accuracy. The lower limit of quantification (LLOQ) was defined as the lowest analyte concentration that can be quantified with acceptable (predefined goals for bias and imprecision) precision and accuracy.

*CRP immunoassay*

CRP measurements in serum and CSF were performed with a turbidimetric immunoassay on a DxAU 5811 automated chemistry analyzer (Beckman-Coulter, Brea, CA, USA). CRP measurements in serum are ISO9001 certified. CRP measurements in CSF are not certified.

*ELISA’s*
sC5b-9 was detected in serum and CSF by in-house ELISA as described previously.^3^ Fully complement activated serum was used as a standard and was assigned 1000 AE/ml. Eculizumab concentration was also measured by in-house ELISA. In short, C5 was coated on the plate, eculizumab from the sample was allowed the bind and detected by an HRP-labeled anti-IgG4 antibody. Eculizumab diluted in buffer, was used as a standard, to calculate the concentration in the sample. Functional activity of complement pathways was measured with an ELISA-based complement system screen assay (WIESLAB® Complement System Screen COMPL 300; Euro-Diagnostica) and was performed according to the manufacturer’s instructions.

**Table S2. Infections and their pathogens**

| **Treatment allocation** | **Type of infection per patient** | **Culture** | **Pathogen** |
| --- | --- | --- | --- |
| Eculizumab | Meningitis, drain-related | CSF | *Candida Albicans* |
|  | Meningitis, drain-related | CSF | *Staphylococcus epidermidis (CNS)* |
| Care as usual | Meningitis, drain-related | Drain tip  CSF | *Staphylococcus epidermidis (CNS)*  Negative |
|  | Meningitis, drain-related  Bacteremia  Pneumonia | CSF  Blood  Central venous catheter tip  Bronchoalveolar lavage fluids | *Staphylococcus epidermidis (CNS)*  *Staphylococcus hemolyticus (CNS)*  *Staphylococcus epidermidis (CNS)*  *Staphylococcus epidermidis (CNS)*  *Staphylococcus hemolyticus (CNS)*  No pathogenic micro-organisms |
|  | Bacteremia  Pneumonia | Blood  Bronchoalveolar lavage fluids and sputum culture | *Methicillin-resistant staphylococcus aureus*  *Methicillin-resistant staphylococcus aureus* |
|  | Upper respiratory tract infection | No culture available | No culture available |

CSF= cerebrospinal fluid; CNS= central nervous system.

**Figure S1. Eculizumab concentration in CSF on day 3**


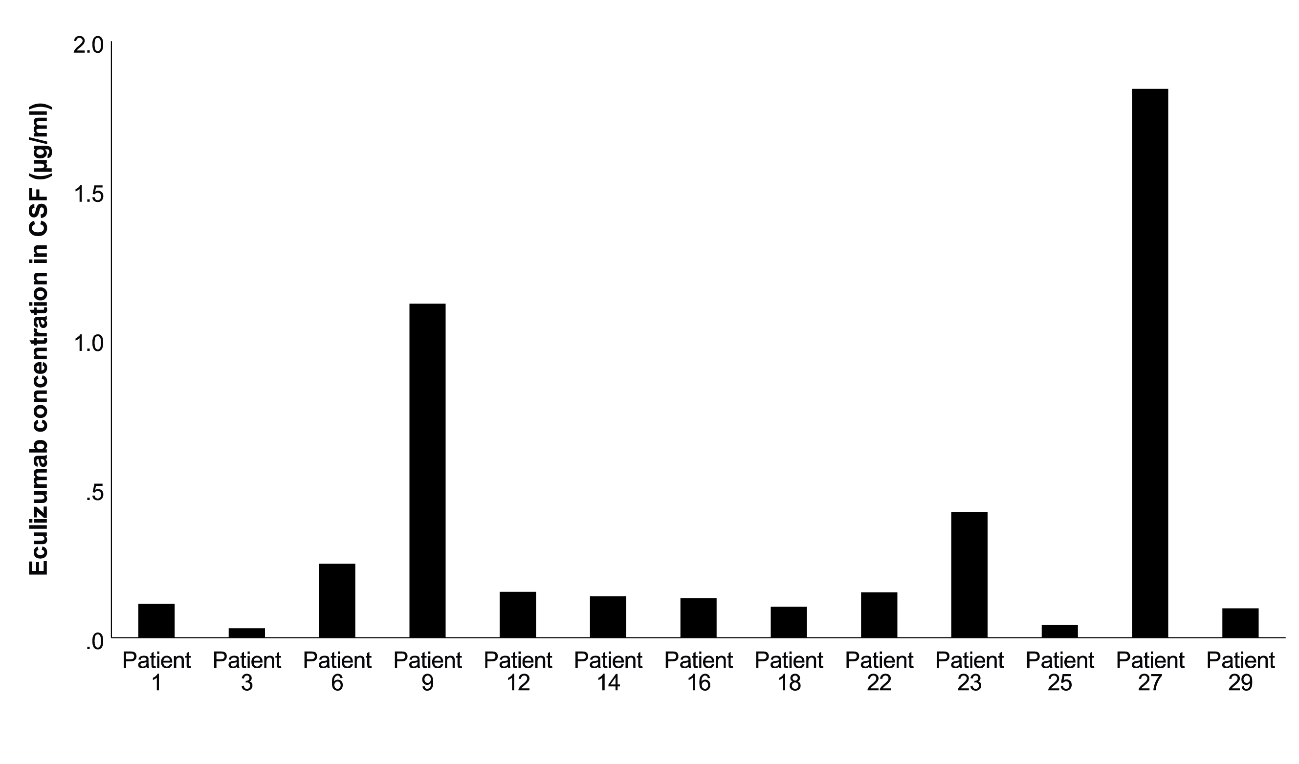


CSF= cerebrospinal fluid.

**Table S3. Secondary CSF outcomes**

|  | n | Eculizumab  N=13 | n | Care as usual  N=13 | P-value |
| --- | --- | --- | --- | --- | --- |
| Secondary CSF outcomes | | | | | |
| IL-6 concentration pg/ml [IQR] | 13 | 1104.2 [281.8-3119.0] | 13 | 1779.8 [353.3-2525.1] | 0.76 |
| IL-10 concentration pg/ml (95% CI) | 13 | 11.5 (6.2-16.7) | 13 | 14.6 (10.4-18.9) | 0.31 |
| IL-18 concentration pg/ml [IQR] | 13 | 7.5 [5.1-12.2] | 13 | 8.4 [6.4-11.4] | 0.61 |
| MCP-1 concentration pg/ml (95% CI) | 13 | 2380.0 (1703.2-3056.8) | 13 | 2385.2 (1264.9-3505.6) | 0.99 |
| sICAM concentration pg/ml [IQR] | 13 | 3620.0 [2198.1-5038.6] | 13 | 3582.1 [2261.7-7707.8] | 0.76 |
| sVCAM concentration pg/ml [IQR] | 13 | 16363.6 [8594.6-25378.1] | 13 | 20195.7 [11467.3-31469.6] | 0.39 |
| P-selectin concentration pg/ml [IQR] | 13 | 15468.0 [6180.8-21737.0] | 13 | 11866.6 [5477.6-26049.3] | 0.80 |
| E-selectin concentration pg/ml (95% CI) | 13 | 834.1 [190.2-3100.1] | 13 | 2348.5 (224.7-4184.2) | 0.24 |
| TNF-a concentration pg/ml [IQR] | 13 | 6.6 [5.3-7.7] | 13 | 6.3 (5.7-7.4) | 0.87 |
| MIF concentration pg/ml [IQR] | 13 | 3417.0 [2291.3-14712.6] | 13 | 4510.2 [2400.4-11599.6] | 1.0 |
| sC5b-9 concentration AE/ml [IQR] | 13 | 1.1 [0.5-2.4] | 13 | 1.1 [0.5-3.6] | 0.81 |
| CRP concentration mg/L [IQR] | 13 | 0.5 [0.5-0.5] | 13 | 0.5 [0.5-0.5] | 0.74 |

IL-1B and sCD163 values were below the limit of detection in more than half of the patients. Mean or median values are therefore not reported for IL-1B and sCD163 and no statistical testing was performed. IQR= interquartile range; CI= confidence interval; IL= interleukin; MCP-1= monocyte chemoattractant protein-1; sICAM= soluble intercellular adhesion molecule; sVCAM= soluble vascular cell adhesion molecule; TNF-a= tumor necrosis factor alpha; MIF= macrophage migration inhibitory factor (MIF); sC5b-9= soluble C5b-9; and CRP= C-reactive protein.

**Figure S2. IL-6 concentration in CSF**


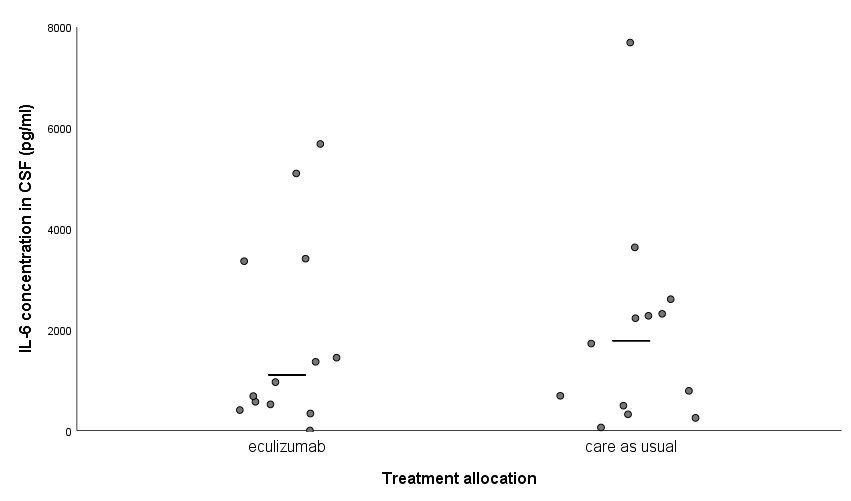


Points in the graph represent individual patient data. The horizontal line represents the median concentration in each group. IL= interleukin; CSF= cerebrospinal fluid.


**Figure S3. IL-10 concentration in CSF**


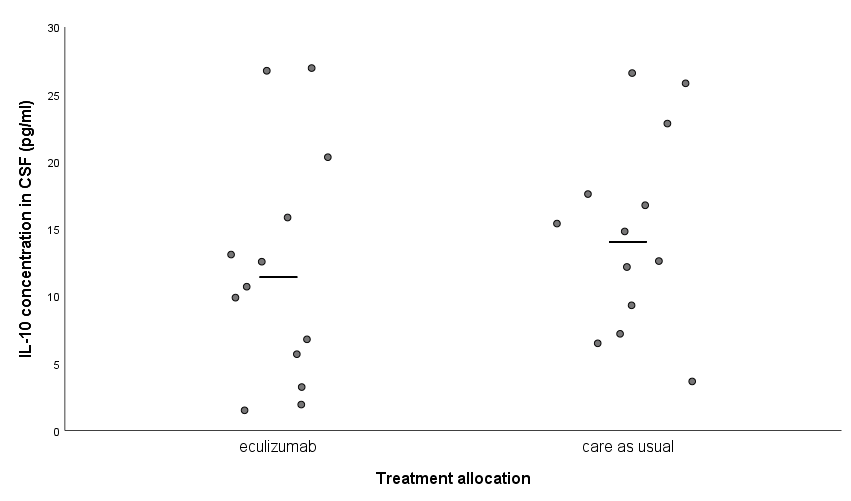


Points in the graph represent individual patient data. The horizontal line represents the median concentration in each group. IL= interleukin; CSF= cerebrospinal fluid.

**Figure S4. IL-18 concentration in CSF**


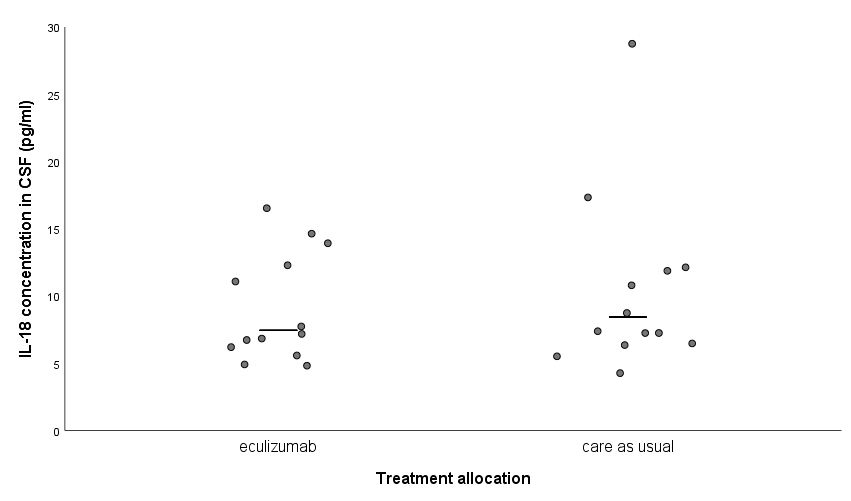


Points in the graph represent individual patient data. The horizontal line represents the median concentration in each group. IL= interleukin; CSF= cerebrospinal fluid.

**Figure S5. MCP-1 concentration in CSF**


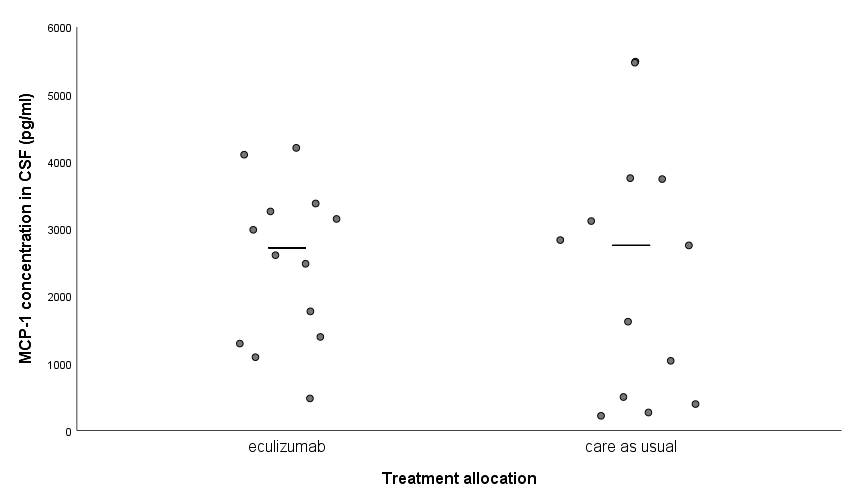


Points in the graph represent individual patient data. The horizontal line represents the median concentration in each group. MCP-1= monocyte chemoattractant protein-1; CSF= cerebrospinal fluid.

**Figure S6. sICAM concentration in CSF**


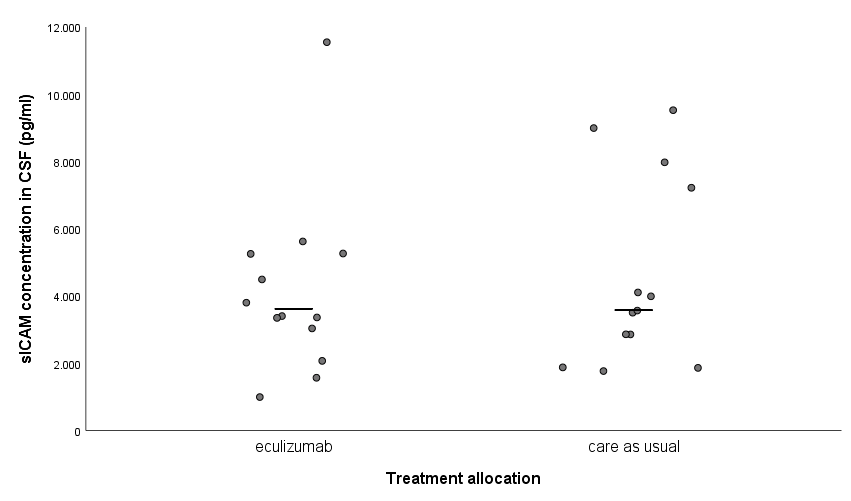


Points in the graph represent individual patient data. The horizontal line represents the median concentration in each group. sICAM= soluble intercellular adhesion molecule; CSF= cerebrospinal fluid.


**Figure S7. sVCAM concentration in CSF**


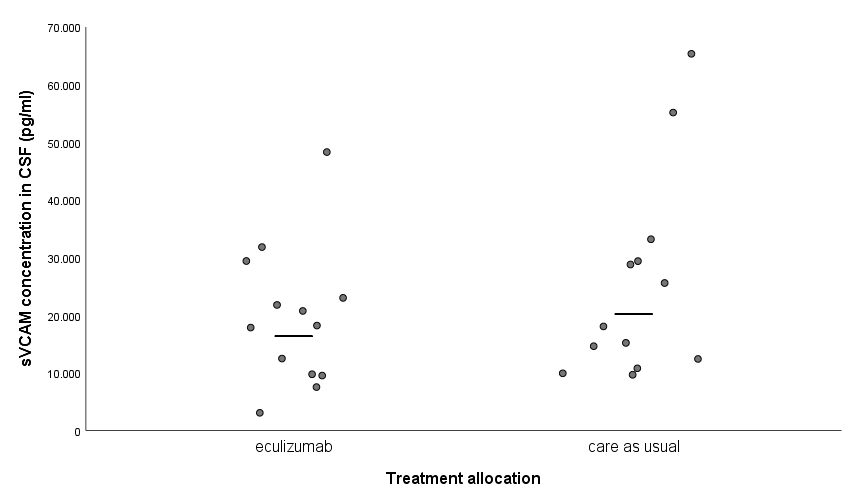


Points in the graph represent individual patient data. The horizontal line represents the median concentration in each group. sVCAM= soluble vascular cell adhesion molecule; CSF= cerebrospinal fluid.

**Figure S8. P-selectin concentration in CSF**


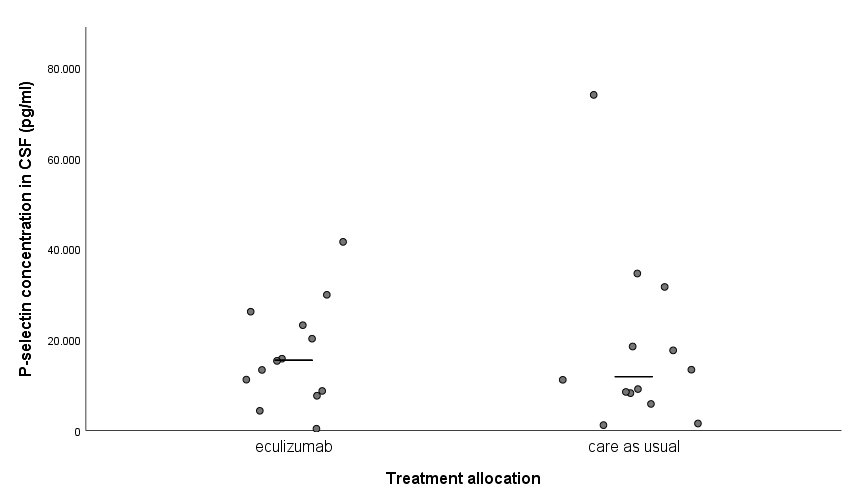


Points in the graph represent individual patient data. The horizontal line represents the median concentration in each group. CSF= cerebrospinal fluid.


**Figure S9. E-selectin concentration in CSF**


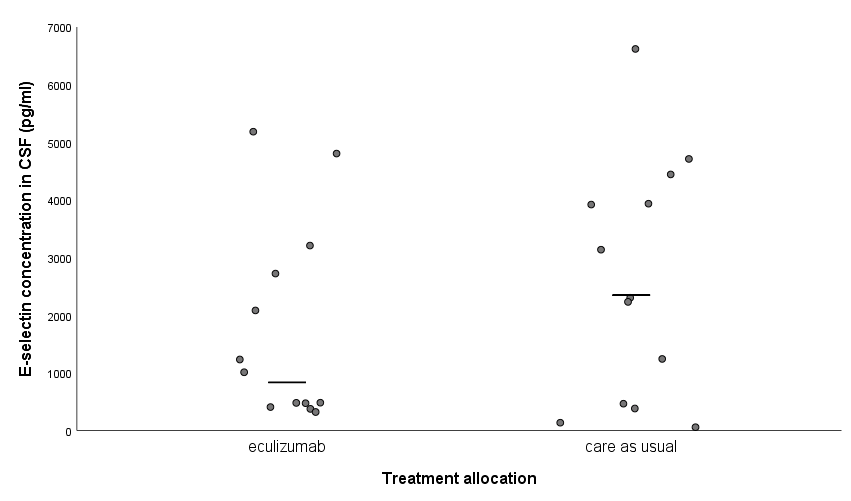


Points in the graph represent individual patient data. The horizontal line represents the median concentration in each group. CSF= cerebrospinal fluid.

**Figure S10. TNF-α concentration in CSF**


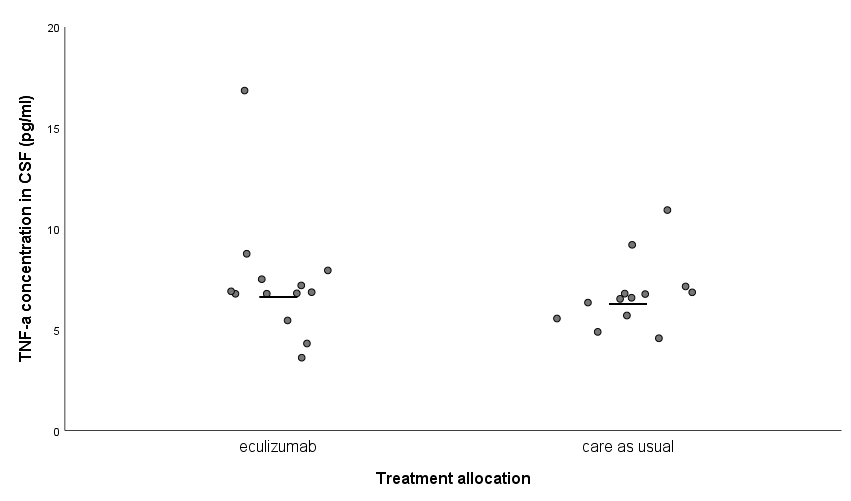


Points in the graph represent individual patient data. The horizontal line represents the median concentration in each group. TNF-a= tumor necrosis factor alpha; CSF= cerebrospinal fluid.


**Figure S11. MIF concentration in CSF**


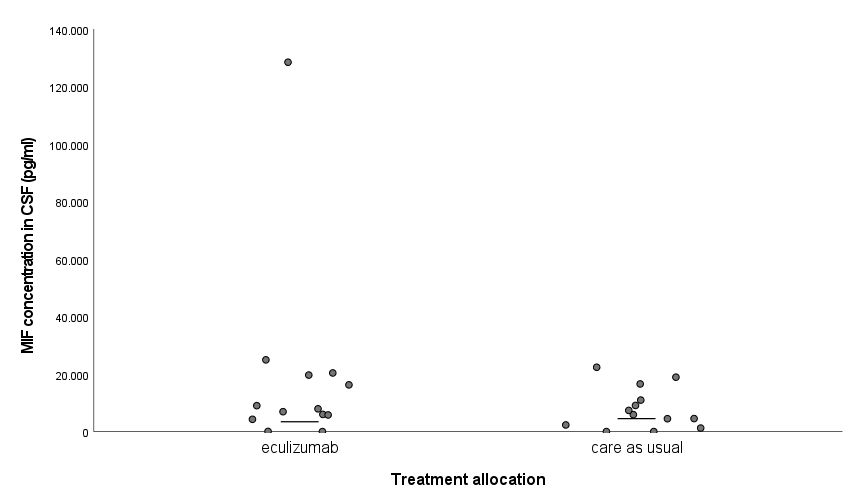


Points in the graph represent individual patient data. The horizontal line represents the median concentration in each group. MIF= macrophage migration inhibitory factor (MIF); CSF= cerebrospinal fluid.

**Figure S12. sC5b-9 concentration in CSF**


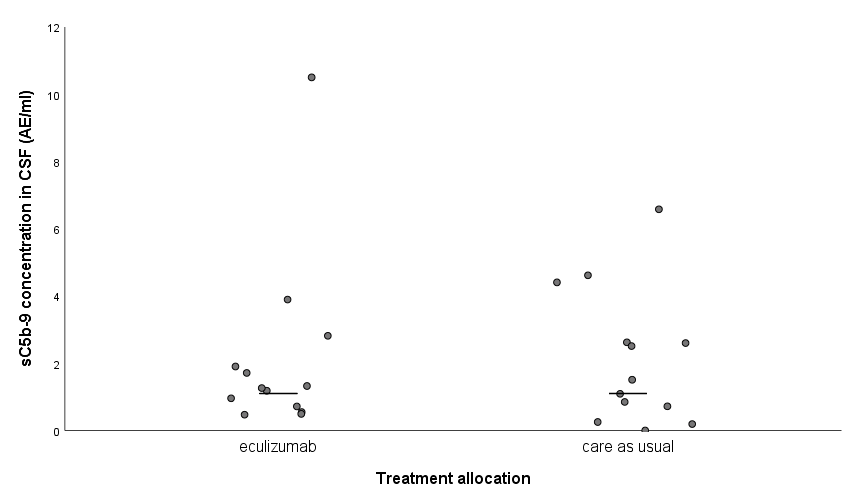


Points in the graph represent individual patient data. The horizontal line represents the median concentration in each group. sC5b-9= soluble C5b-9; CSF= cerebrospinal fluid.

Concentration CRP in CSF: no scatterplot was made as the median and IQR (25-75) were 0.5 mg/L in both the intervention and care as usual group (Table S2.)

**Figure S13. Eculizumab concentration in serum**


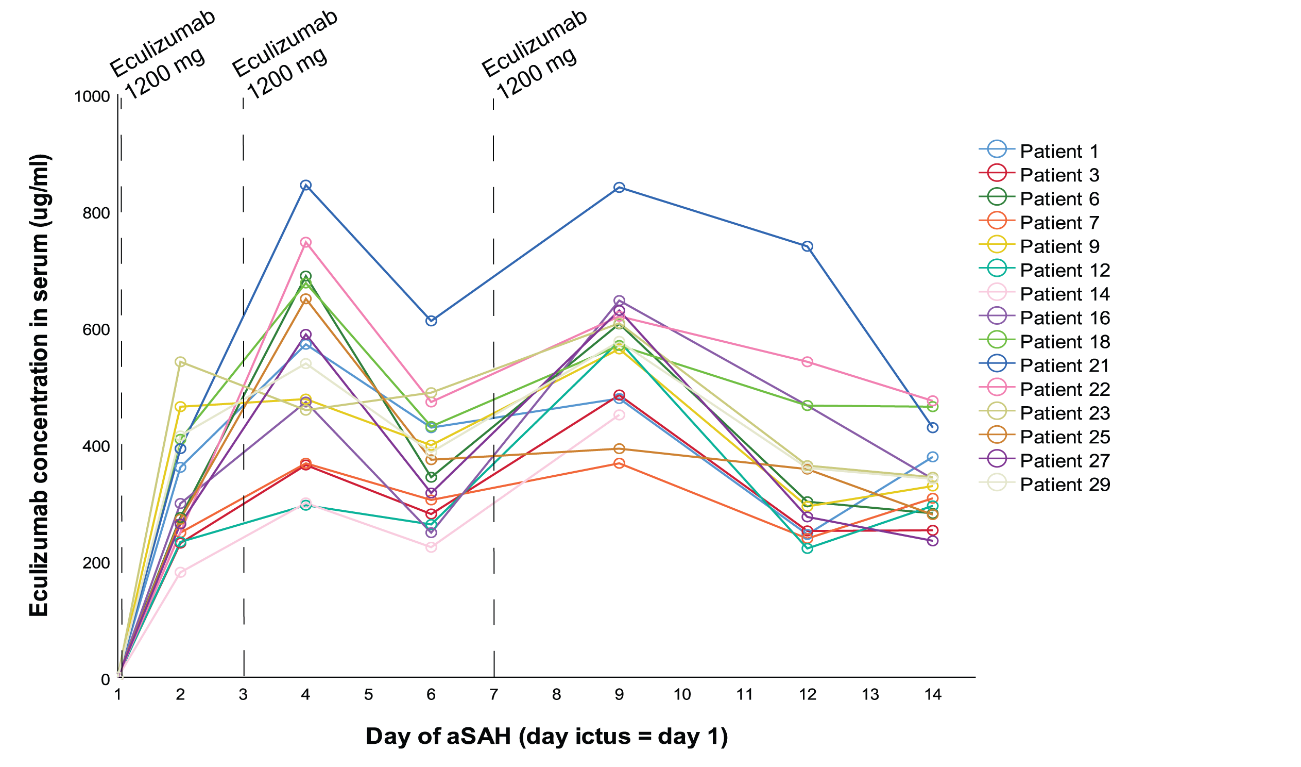


Patients allocated to eculizumab treatment received intravenous infusion with eculizumab 1200 mg at three different time points: <12h, on day 3, and day 7 after ictus. aSAH= aneurysmal subarachnoid hemorrhage.

| Day | n | Eculizumab  Mean (95% CI)/median [IQR] | n | Care as usual  Mean (95% CI)/median [IQR] | P-value |
| --- | --- | --- | --- | --- | --- |
| Day 1 | 14 | 209749.7 (146486.1-273013.2) | 16 | 168811.6 (104020.7-233602.5) | 0.34 |
| Day 2 | 14 | 3704.4 [2773.6-4745.8] | 15 | 170146.9 [42467.6-227816.8] | <0.01 |
| Day 4 | 15 | 7078.3 [5047.0-8272.9] | 16 | 179618.3 [79235.9-255255.2] | <0.01 |
| Day 6 | 15 | 9375.7 [7624.3-12719.1] | 16 | 180434.0 [92712.5-301457.9] | <0.01 |
| Day 9 | 15 | 9860.2 [7394.6-13073.5] | 16 | 152340.2 [87154.2-277837.9] | <0.01 |
| Day 12 | 14 | 10402.2 [8467.9-15325.6] | 16 | 181564.3 [88059.6-265649.7] | <0.01 |
| Day 14 | 14 | 11318.1 [7375.2-14122.8] | 11 | 161912.0 [62519.6-275922.3] | <0.01 |

**Table S4. C5a concentration in serum (pg/ml)**

CI= confidence interval; and IQR= interquartile range.

**Table S5. CH50% activity in serum**

| Day | n | Eculizumab  Mean (95% CI)/median [IQR] | n | Care as usual  Mean (95% CI)/median [IQR] | P-value |
| --- | --- | --- | --- | --- | --- |
| Day 1 | 14 | 101.5 (95.8-107.3) | 16 | 101.6 (92.3-110.9) | 0.99 |
| Day 2 | 14 | 0.4 [0.3-0.6] | 15 | 98.3 [85.6-114.1] | <0.01 |
| Day 4 | 15 | 0.4 [0.4-0.6] | 16 | 115.9 [108.1-124.7] | <0.01 |
| Day 6 | 15 | 0.8 [0.7-1.3] | 16 | 125.2 [120.2-130.6] | <0.01 |
| Day 9 | 15 | 0.7 [0.6-1.0] | 16 | 123.5 [119.7-132.8] | <0.01 |
| Day 12 | 14 | 1.0 [0.9-1.4] | 16 | 127.1 [119.4-131.2] | <0.01 |
| Day 14 | 14 | 1.2 [1.0-1.8] | 11 | 121.1 [118.2-133.5] | <0.01 |

CH50%= assay for the functional complement activity of the classical pathway; CI= confidence interval; and IQR= interquartile range.

| Day | n | Eculizumab  Median [IQR] | n | Care as usual  Median [IQR] | P-value |
| --- | --- | --- | --- | --- | --- |
| Day 1 | 14 | 53.2 [26.4-74.9] | 16 | 39.0 [21.3-96.0] | 0.98 |
| Day 2 | 14 | 0.1 [0.0-0.4] | 15 | 49 [16.0-70.6] | <0.01 |
| Day 4 | 15 | 0.1 [0.0-0.1] | 16 | 55.0 [26.8-91.2] | <0.01 |
| Day 6 | 15 | 0.4 [0.0-0.5] | 16 | 80.8 [52.4-100.2] | <0.01 |
| Day 9 | 15 | 0.2 [0.0-0.4] | 16 | 86.8 [40.7-104.7] | <0.01 |
| Day 12 | 14 | 0.4 [0.0-0.7] | 16 | 101.6 [70.3-112.6] | <0.01 |
| Day 14 | 14 | 0.4 [0.2-0.7] | 11 | 89.1 [67.7-108.5] | <0.01 |

**Table S6. AP50% activity in serum**AP50%= assay for the functional complement activity of the alternative pathway; IQR= interquartile range.

| Day | n | Eculizumab  Median [IQR] | n | Care as usual  Median [IQR] | P-value |
| --- | --- | --- | --- | --- | --- |
| Day 1 | 14 | 44.1 [6.0-81.3] | 16 | 46.8 [11.0-88.0] | 0.64 |
| Day 2 | 14 | 0.1 [0.0-0.8] | 15 | 28.7 [1.6-80.8] | <0.01 |
| Day 4 | 15 | 0.5 [0.0-0.7] | 16 | 48.8 [6.2-107.0] | <0.01 |
| Day 6 | 15 | 0.7 [0.1-2.2] | 16 | 92.8 [15.9-122.1] | <0.01 |
| Day 9 | 15 | 1.3 [0.3-2.0] | 16 | 114.4 [10.8-137.4] | <0.01 |
| Day 12 | 14 | 1.4 [0.6-2.8] | 16 | 111.0 [33.1-139.1] | <0.01 |
| Day 14 | 14 | 1.5 [0.7-2.7] | 11 | 126.5 [0.4-139.0] | 0.03 |

**Table S7. MBL% activity in serum**

MBL%= assay for the functional complement activity of the mannose-binding lectin pathway; IQR= interquartile range.

**Figure S14. Median complement activity of the classical pathway in serum**

On day 1 the mean value with 95% CI is depicted. The other values are medians with IQRs. Error bars represent 95% CI (on day 1) or IQR values (25-75th percentiles). CH50%= assay for the functional complement activity of the classical pathway; aSAH= aneurysmal subarachnoid hemorrhage; CI= confidence interval; and IQR= interquartile range.

**Figure S15. Median AP50% activity in serum**

Error bars represent IQR values (25-75^th^ percentiles). AP50%= assay for the functional complement activity of the alternative pathway; aSAH=aneurysmal subarachnoid hemorrhage.

**Figure S16. Median MBL% activity in serum**

Error bars represent IQR values (25-75^th^ percentiles). MBL%= assay for the functional complement activity of the mannose-binding lectin pathway; aSAH=aneurysmal subarachnoid hemorrhage.

**Table S8. IL-6 concentration in serum (pg/ml)**

| Day | n | Eculizumab  Median [IQR] | n | Care as usual  Median [IQR] | P-value |
| --- | --- | --- | --- | --- | --- |
| Day 1 | 14 | 22.7 [6.6-36.8] | 16 | 7.2 [4.7-39.9] | 0.69 |
| Day 2 | 14 | 14.6 [4.7-19.7] | 15 | 10.8 [2.6-24.2] | 0.72 |
| Day 4 | 15 | 7.3 [4.0-12.1] | 16 | 6.5 [2.4-13.0] | 0.75 |
| Day 6 | 15 | 3.7 [2.4-8.5] | 16 | 3.4 [2.4-15.2] | 0.73 |
| Day 9 | 15 | 2.6 [2.4-7.0] | 16 | 4.4 [2.4-10.0] | 0.24 |
| Day 12 | 14 | 2.4 [2.4-3.9] | 16 | 3.7 [3.0-6.4] | 0.02 |
| Day 14 | 14 | 2.4 [2.4-3.1] | 11 | 2.4 [2.4-18.0] | 0.21 |

IL=interleukin; IQR= interquartile range.

**Table S9. IL-10 concentration in serum (pg/ml)**

| Day | n | Eculizumab  Median [IQR] | n | Care as usual  Median [IQR] | P-value |
| --- | --- | --- | --- | --- | --- |
| Day 1 | 14 | 5.8 [1.9-18.2] | 16 | 6.2 [3.2-9.6] | 0.93 |
| Day 2 | 14 | 3.7 [1.2-9.3] | 15 | 3.0 [2.3-6.8] | 0.87 |
| Day 4 | 15 | 2.6 [2.0-5.2] | 16 | 2.4 [1.1-4.6] | 0.36 |
| Day 6 | 15 | 2.9 [1.8-3.9] | 16 | 1.2 [0.9-2.4] | 0.01 |
| Day 9 | 15 | 2.0 [1.0-4.1] | 16 | 2.3 [1.4-3.4] | 0.96 |
| Day 12 | 14 | 2.0 [0.9-3.3] | 16 | 2.1 [0.9-3.9] | 0.96 |
| Day 14 | 14 | 1.9 [1.2-2.8] | 11 | 2.2 [0.9-5.6] | 0.60 |

IL=interleukin; IQR= interquartile range.

| Day | n | Eculizumab | n | Care as usual |
| --- | --- | --- | --- | --- |
| Day 1 | 15 | Within normal range | 16 | Within normal range |
| Day 2 | 14 | Within normal range | 15 | Within normal range |
| Day 4 | 15 | Within normal range | 16 | Within normal range |
| Day 6 | 15 | Within normal range | 16 | Within normal range |
| Day 9 | 15 | Within normal range | 16 | Within normal range |
| Day 12 | 14 | Within normal range | 16 | Within normal range |
| Day 14 | 14 | Within normal range | 11 | Within normal range |

**Table S10. sC5b-9 values in serum (AE/ml)**

Median and IQR (25-75^th^ percentiles) values were <2 AE/ml in both the intervention and care as usual group.

**Table S11. CRP concentration in serum (mg/L)**

| Day | n | Eculizumab  Median [IQR] | n | Care as usual  Median [IQR] | P-value |
| --- | --- | --- | --- | --- | --- |
| Day 1 | 15 | 2.8 [1.0-4.0] | 16 | 1.4 [0.6-6.5] | 0.72 |
| Day 2 | 14 | 7.5 [3.8-33.3] | 15 | 12.0 [9.0-40.0] | 0.24 |
| Day 4 | 15 | 34.0 [19.0-46.0] | 16 | 24.5 [9.9-106.3] | 0.44 |
| Day 6 | 15 | 19.0 [15.0-34.0] | 16 | 13.5 [7.0-53.5] | 0.44 |
| Day 9 | 15 | 13.0 [7.0-20.0] | 16 | 9.5 [3.3-15.3] | 0.26 |
| Day 12 | 14 | 5.1 [3.8-12.3] | 16 | 12.0 [5.0-40.0] | 0.11 |
| Day 14 | 14 | 3.5 [2.8-6.0] | 11 | 16.0 [5.0-26.0] | 0.01 |

CRP= C-reactive protein; IQR= interquartile range.

**Figure S17. Median IL-6 concentration in serum**

Error bars represent IQR values (25-75^th^ percentiles). IL=interleukin; aSAH=aneurysmal subarachnoid hemorrhage.


F**igure S18. Median concentration IL-10 in serum**

Error bars represent IQR values (25-75^th^ percentiles). IL=interleukin; aSAH=aneurysmal subarachnoid hemorrhage.

**Figure S19. Median CRP concentration in serum**

Error bars represent IQR values (25-75^th^ percentiles). CRP= C-reactive protein; aSAH=aneurysmal subarachnoid hemorrhage.

**Figure S20. Glasgow Coma Scale per day in the care as usual group**

**
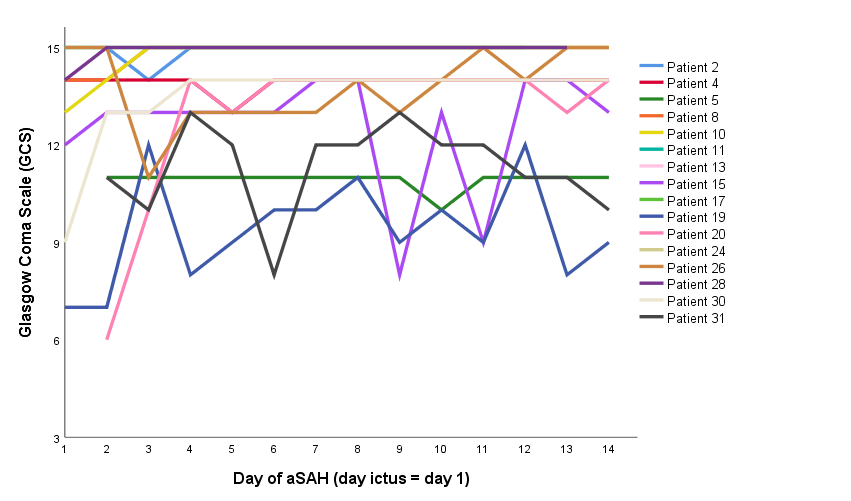
**

The Glasgow Coma Scale (GCS) score for day 1 is missing in four patients who were intubated and sedated on admission to the tertiary referral center. GCS= Glasgow Coma Scale; aSAH= aneurysmal subarachnoid hemorrhage.

**Figure S21. Glasgow Coma Scale per day in the intervention group**


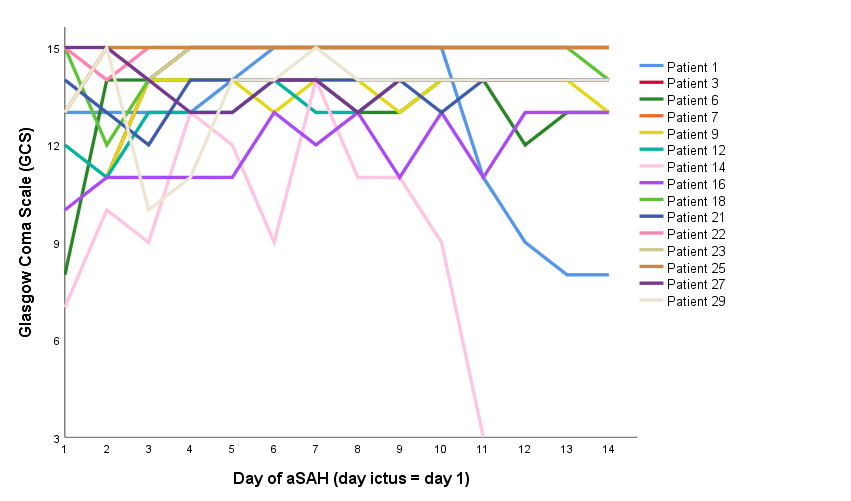


The Glasgow Coma Scale (GCS) score for day 1 is missing in two patients who were intubated and sedated on admission to the tertiary referral center. GCS= Glasgow Coma Scale; aSAH= aneurysmal subarachnoid hemorrhage.

**Figure S22. modified Rankin Scale (mRS) score at 10-15 weeks according to treatment allocation**

mRS= modified Rankin Scale.

**Table S12. C5a concentration in CSF by sample type**

| C5a concentration in CSF by sample type pg/ml (95% CI) | N | Eculizumab | N | Care as usual | P-value |
| --- | --- | --- | --- | --- | --- |
| Lumbar puncture | 5 | 276 (90-462) | 8 | 210 (96-325) | 0.43 |
| External ventricular drain | 8 | 262 (63-462) | 5 | 798 (253-1342) | <0.05 |

CSF= cerebrospinal fluid; CI= confidence interval; and IQR= interquartile range.

**Table S13. PAASH and Hijdra sum score by sample type**

|  | N | CSF samples obtained by lumbar puncture | N | CSF samples obtained from an EVD | P-value |
| --- | --- | --- | --- | --- | --- |
| Median PAASH score [IQR]^*^ | 13 | 2 [2-3] | 13 | 3 [1-5] | 0.28 |
| Median Hijdra sum score [IQR] ^†^ | 13 | 24 [21-27] | 13 | 32 [26-34] | <0.01 |

^*^ If the patient was intubated and sedated on admission to the tertiary referral center, we used the PAASH score based on the GCS score before intubation. ^†^Hijdra sum score was determined on the head computed tomography (CT) scan performed on admission to the tertiary center unless no new head CT scan was made, in which case the head CT scan of the referring center was used. PAASH= Prognosis on Admission of Aneurysmal Subarachnoid Hemorrhage; IQR= interquartile range; and EVD= external ventricular drain.

**References**

1. Steiner T, Juvela S, Unterberg A, et al. European Stroke Organization guidelines for the management of intracranial aneurysms and subarachnoid haemorrhage. *Cerebrovasc Dis* 2013; 35: 93–112.

2. Scholman RC, Giovannone B, Hiddingh S, et al. Effect of anticoagulants on 162 circulating immune related proteins in healthy subjects. *Cytokine* 2018; 106: 114–124.

3. Bergseth G, Ludviksen JK, Kirschfink M, et al. An international serum standard for application in assays to detect human complement activation products. *Mol Immunol* 2013; 56: 232–239.
