# Supplementary material for: Safety and pharmacodynamic efficacy of eculizumab in aneurysmal subarachnoid hemorrhage (CLASH): A phase 2a randomized clinical trial
Source: Eur Stroke J. 2023 Aug 22;8(4):1097–106. doi: 10.1177/23969873231194123 (PMC10683736; doi:10.1177/23969873231194123)
Supplement: sj-docx-2-eso-10.1177_23969873231194123 – Supplemental material for Safety and pharmacodynamic efficacy of eculizumab in aneurysmal subarachnoid hemorrhage (CLASH): A phase 2a randomized clinical trial [file sj-docx-2-eso-10.1177_23969873231194123.docx]

**Supplementary material 2**

**Safety and pharmacodynamic efficacy of eculizumab in aneurysmal subarachnoid hemorrhage** **(CLASH): a phase 2a, randomized, controlled trial**

Inez Koopman, MD; Reinier W.P. Tack, MD; Herman F. Wunderink, MD PhD; Anke H.W. Bruns, MD PhD; Irene van der Schaaf, MD PhD; Daniela Cianci, PhD; Kyra A. Gelderman, MD PhD; Inge M. van de Ridder, MD; Elly M. Hol, PhD; Gabriel J.E. Rinkel, MD PhD FRCP(E); Mervyn D.I. Vergouwen, MD PhD

**Table of contents**

Statistical analysis plan p. 2-22

STATISTICAL ANALYSIS PLAN

**CompLement C5 Antibodies for decreasing brain injury after aneurysmal Subarachnoid Hemorrhage**

***Safety and proof-of-concept***

**Version 2.0 Date 17-11-2021**

Based on version 11.0 dated 08-07-2019 of the protocol

|  |  | Title | Date |
| --- | --- | --- | --- |
| Written by | I. Koopman | MD | 17-11-2021 |
| Reviewed by | D. Ciancia M.D.I. Vergouwen | PhD MD, PhD | 20-11-2021 |
| Approved by | D. Ciancia M.D.I. Vergouwen | PhD MD, PhD | 20-11-2021 |

| Study title and Trial registration | CLASH study NTR: NTR6752 Eudra-CT: 2017-004307-51 |
| --- | --- |
| SAP version | V 2.0 |
| Version date | 17-11-2021 |
| Based on study protocol version | V 11.0 |
| Principal Investigator Affiliation | Dr. M.D.I. Vergouwen, MD PhD Neurologist Department of Neurology and Neurosurgery, UMC Utrecht Brain Center, University Medical Center Utrecht Room: G3-228 Phone: 088 755 0455 Email: M.D.I.Vergouwen@umcutrecht.nl |
| Investigators  Affiliations | I. Koopman, MD PhD candidate  Department of Neurology and Neurosurgery, UMC Utrecht Brain Center, University Medical Center Utrecht Matthias van Geuns building  Room: 02.15 Phone: 088-7571441 Email: I.Koopman-4@umcutrecht.nl |
| Statisticians Affiliations | Daniela Cianci, PhD Assistant Professor Biostatistics Department of Biostatistics and Research Support  Julius Center for Health Sciences and Primary Care |
| Investigator writing the SAP Affiliation | I. Koopman, MD  Department of Neurology and Neurosurgery, UMC Utrecht Brain Center, University Medical Center Utrecht  In collaboration with: Daniela Cianci, PhD Assistant Professor Biostatistics Department of Biostatistics and Research Support  Julius Center for Health Sciences and Primary Care |
| Data management | I. Koopman, MD  Department of Neurology and Neurosurgery, Brain Center Rudolf Magnus, University Medical Center Utrecht |

# **Table of Contents**

#

[Table of Contents 4](#_Toc91145761)

[Abbreviations and definitions 5](#_Toc91145762)

[1 Introduction 6](#_Toc91145763)

[1.1 Purpose and scope of the statistical analysis plan 6](#_Toc91145764)

[1.2 Description of the study 6](#_Toc91145765)

[2 Objectives 8](#_Toc91145766)

[2.1 Primary study objective 8](#_Toc91145767)

[2.2 Secondary study objectives 8](#_Toc91145768)

[3 Study design 8](#_Toc91145769)

[3.1 Sample size 8](#_Toc91145770)

[3.2 Randomization 8](#_Toc91145771)

[4 Outcome measures 8](#_Toc91145772)

[4.1 Primary outcome parameter 8](#_Toc91145773)

[4.2 Secondary outcome parameter(s) 9](#_Toc91145774)

[4.3 safety parameters 9](#_Toc91145775)

[5 Study Committees 9](#_Toc91145776)

[6 Analysis – General considerations 9](#_Toc91145777)

[6.1 Interim analysis 9](#_Toc91145778)

[6.2 Handling of missing data 9](#_Toc91145779)

[6.3 Handling multi-center 9](#_Toc91145780)

[6.4 Handling multiple testing 9](#_Toc91145781)

[6.5 Handling robustness and sensitivity testing 9](#_Toc91145782)

[6.6 Handling visit windows 10](#_Toc91145783)

[6.7 Outlier management 10](#_Toc91145784)

[6.8 Statistical computer software 10](#_Toc91145785)

[6.9 Format tables and graphs 10](#_Toc91145786)

[7 Definition of population for analysis 19](#_Toc91145787)

[7.1 Subjects flowchart 19](#_Toc91145788)

[7.2 Subject Data Sets 19](#_Toc91145789)

[8 Statistical analysis 19](#_Toc91145790)

[8.1 Descriptive statistics 19](#_Toc91145791)

[8.2 Analysis of primary outcome 19](#_Toc91145792)

[8.3 Analysis of secondary outcome 20](#_Toc91145793)

[8.4 Subgroup analysis 20](#_Toc91145794)

[9 SAFETY ANALYSIS 20](#_Toc91145795)

[10 CHANGES TO THE PROTOCOL OR PREVIOUS VERSIONS OF SAP 21](#_Toc91145796)

[11 REFERENCES 22](#_Toc91145797)

#

# **Abbreviations and definitions**

| **ANCOVA**  **AE**  **aHUS**  **CI**  **CSF**  **CT**  **DCI**  **DSMB**  **EQ-5D-5L** | **Analysis of covariance**  **Adverse Event**  **Atypical Hemolytic Uremic Syndrome**  **Confidence interval**  **Cerebrospinal fluid**  **Computed tomography**  **Delayed cerebral ischemia**  **Data Safety Monitoring Board**  **EuroQol 5-dimensions 5-levels** |
| --- | --- |
| **EudraCT**  **GCS** | **European drug regulatory affairs Clinical Trials**  **Glasgow Coma Scale** |
| **Hijdra score**  **IQR**  **ITT**  **MoCA**  **MRI**  **mRS**  **NIHSS**  **PAASH**  **PP**  **PNH** | **A score to assess the amount of blood after subarachnoid hemorrhage on computed tomography**  **Interquartile range**  **Intention-to-treat**  **Montreal Cognitive Assessment**  **Magnetic Resonance Imaging**  **modified Rankin Scale**  **National Institutes of Health Stroke Scale**  **Prognosis on Admission of Aneurysmal Subarachnoid Hemorrhage**  **Per protocol**  **Paroxysmal Nocturnal Hemoglobinuria** |
| **QoL**  **(S)AE**  **SAH**  **SD**  **SNP** | **Quality of life**  **(Serious) Adverse Event**  **Subarachnoid hemorrhage**  **Standard deviation**  **Single Nucleotide Polymorphism** |
| **SUSAR**  **UMCU** | **Suspected Unexpected Serious Adverse Reaction**  **University Medical Centre Utrecht** |
| **WFNS** | **World Federation of Neurosurgical Societies** |

# **Introduction**

## Purpose and scope of the statistical analysis plan

To describe the planned statistical analysis of the CLASH trial.

## Description of the study

Aneurysmal subarachnoid hemorrhage (SAH) is a subtype of stroke with a median age of onset of 55 years.^1^ Although SAH is less common than ischemic stroke, the loss of productive life years after SAH is similar to that after ischemic stroke, which is due to the young age of SAH patients and its poor prognosis.^2^ Important determinants of poor functional outcome after SAH are early brain injury (brain injury <72 hours after ictus) and delayed cerebral ischemia (4-14 days after the bleeding).^3,4^ No treatment exists to reduce early brain injury and the effects of current strategies (nimodipine, euvolemia) to prevent delayed cerebral ischemia are only modest.^5^ With the current treatment standards, approximately 1/3 of all patients with aneurysmal SAH dies within 90 days, 1/3 remains dependent, and 1/3 has no or minor impairments.^6^ Because of this poor prognosis, new treatment options are needed to reduce brain injury and improve prognosis.

The inflammatory response is considered to play a key role in the pathogenesis of early brain injury and delayed cerebral ischemia after aneurysmal SAH. Previous studies found that the complement cascade is activated in patients with SAH and associated with poor functional outcome.^7–12^ Several studies were performed to investigate if: 1) previously observed associations between complement activation and outcome after SAH also imply causal relationships; and 2) C5 antibodies can potentially reduce brain injury. These studies include autopsy studies, cerebrospinal fluid (CSF) studies, genetic studies, and animal studies and their results show that:^13^

1. Brains of patients who died from aneurysmal SAH had much higher complement expression than brains from controls. This difference was most pronounced in areas with brain infarction *(Figure 1)*;
2. C5a concentrations in CSF were markedly increased (>1400‐fold increase)) shortly after SAH compared with controls, and decreased over the first 14 days after ictus *(Figure 2)*;
3. Plasma levels of C5a correlated with a C5 single nucleotide polymorphism (SNP) in a Dutch cohort of 930 patients with SAH. In this cohort, the C5 SNP also correlated with functional outcome 3 months after ictus;
4. In a SAH mouse model, brain injury was >40% reduced in C5a receptor knock-out mice and in wild type mice treated with C5 antibodies compared with untreated wild type mice *(Figure 3)*.

Figure 1. Results from autopsy study.

|  | SAH, infarcted area | SAH, non-infarcted area | Control | Comparisons* | |
| --- | --- | --- | --- | --- | --- |
|  |  |  |  | Infarcted vs. non-infarcted | Infarcted vs. control |
| C1q | 0.21 (0.19-0.23) | 0.17 (0.16-0.17) | 0.14 (0.13-0.15) | p<0.001 | p<0.001 |
| C3c | 0.21 (0.20-0.24) | 0.17 (0.17-0.17) | 0.14 (0.13-0.15) | p<0.001 | p<0.001 |

Values represent median optical density values with IQR. * = analyzed with Mann-Whitney U test

Figure 2. Complement component C5a concentrations in cerebrospinal fluid (CSF) after subarachnoid hemorrhage.

**
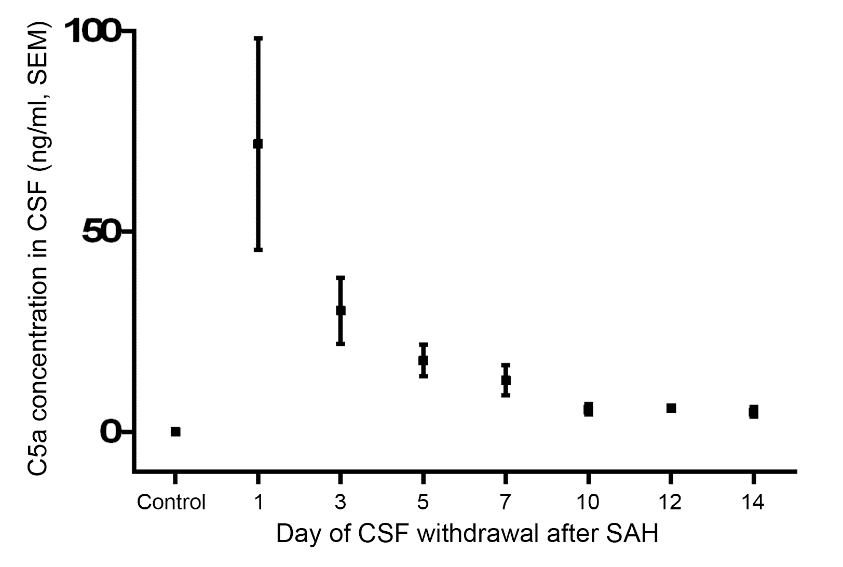
**

Figure 3. Results from animal studies


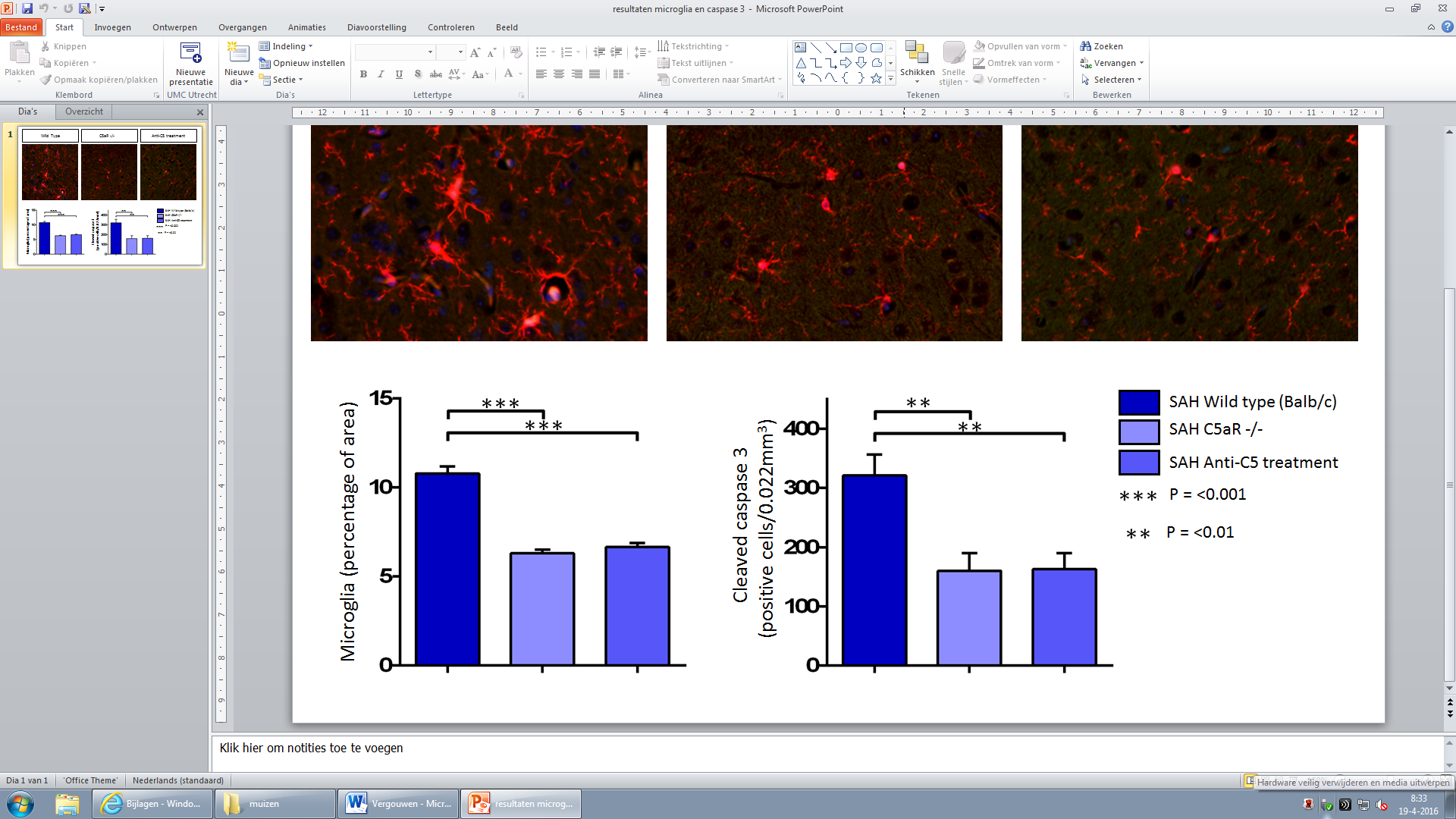


Quantified microglia activation (percentage of area) and apoptosis after subarachnoid hemorrhage in wild type mice (n=14), C5aR-/- mice (n=15) and C5a antibody treated mice (n=15).

From the aforementioned results, we concluded that complement activation is not only associated with brain injury after SAH, but that treatment with C5 antibodies also decreases brain injury in an SAH animal model. Although C5 antibodies (eculizumab) are currently available as a treatment option for patients with Paroxysmal Nocturnal Hemoglobinuria (PNH) and Atypical Hemolytic Uremic Syndrome (aHUS), eculizumab has not yet been administered to patients with aneurysmal SAH. In the current trial, we will investigate the biological effect and safety of eculizumab in patients with aneurysmal SAH.

# **Objectives**

This study aims to investigate the biological effect and safety of eculizumab in patients with aneurysmal SAH.

## Primary study objective

To assess the safety and pharmacodynamic efficacy (proof of-concept) of early and short-term eculizumab administration on C5a levels in the cerebrospinal fluid (CSF) of aSAH patients.

## Secondary study objectives

The secondary outcome measures include blood and CSF parameters of inflammation, quality of life (QoL), functional, and cognitive outcomes, and the presence and volume of cerebral infarction. QoL and functional outcome will be measured by the EuroQol 5-dimensions 5-levels (EQ-5D-5L) questionnaire and the modified Rankin Scale (mRS). Cognitive functioning will be assessed with the use of the Montreal Cognitive Assessment (MoCA).

# **Study design**

## Sample size

Based on a previous study with eculizumab in patients with neuromyelitis optica, we expect a difference in C5 concentration in CSF of 55% between groups with and without treatment with C5 antibodies.^14^ With a standard deviation (SD) of 50%, 5% type I error, and 80% power, we need 13 patients per group (www.clincalc.com/Stats/SampleSize.aspx). The group size will be increased to 20 patients per group, taking into account an assumed mortality rate of 25% and 2 patients per group who refuse a lumbar puncture in a later phase despite giving informed consent earlier.

## Randomization

A computer-generated block randomization is used to randomize patients. No stratification factors were used because all patients were included at the UMCU (University Medical Centre Utrecht).

# **Outcome measures**

## Primary outcome parameter

1) C5a concentration in CSF on day 3

## Secondary outcome parameter(s)

2) Occurrence of AEs and SAEs;
3) Blood and CSF parameters of inflammation (e.g. CRP and cytokines);
4) Concentration of eculizumab in blood and CSF;
5) Daily neurological condition measured by GCS during the first fourteen days of hospital stay;
6) Neurological condition measured by the NIHSS and WFNS score +/- 14 days after ictus;
7) Cerebral infarction on brain MRI at hospital discharge;
8) Cognition and QoL +/- 10 weeks after ictus;
9) The mRS +/- thirteen weeks after ictus.

## **safety parameters**

Occurrence of AEs and SAEs.

# Study Committees

Trial Steering Committee
I. Koopman, MD, PhD candidate

M.D.I. Vergouwen, MD PhD, neurologist

M. Bartels, MD PhD, pediatric hematologist/oncologist

J.P. Greving, PhD, clinical epidemiologist

Prof. A. Slooter, MD PhD, neurologist-intensivist

Prof. A. van der Zwan, MD PhD, neurosurgeon

Prof. G.J.E. Rinkel, MD FRCP(E), neurologist

Data & Safety Monitoring Board

Prof. Dr. J. Stam (chair)

Prof. Dr. D. van de Beek

Dr. H. F. Lingsma

# **Analysis – General considerations**

## Interim analysis

When 20 patients were included, a safety report was sent to the DSMB. The DSMB evaluated the safety report and concluded that there were no reasons to assume that patients with eculizumab had a higher risk of serious complications or side-effects compared to the control group. The DSMB found no therapeutic benefit that would deem continuation of this study unethical. The assessment of therapeutic benefit was based on the number of patients who survived (not on C5a in CSF).

## Handling of missing data

Missing data will not be imputed. We will describe which data are missing and the number of patients with available data.

## Handling multi-center

Not applicable, all patients have been enrolled at the UMCU.

## Handling multiple testing

There will be no multiple testing problem for the primary outcome, as there is only one primary outcome measure. The sample size for this study was based on the primary outcome measure and not on the secondary outcome measures. Secondary outcome measures are supportive to the primary outcome measure and can only be used to confirm the effect seen in the primary outcome measure. Multiple testing will therefore not be corrected for secondary outcome measures.

## Handling robustness and sensitivity testing

For the primary outcome measure, a per protocol (PP) analysis will be performed as primary analysis. An intention to treat (ITT) analysis will be performed as supportive analysis.

Please find details on the sensitivity analysis in section 8.2.

## Handling visit windows

We will describe the following details for each measurement with a visit window:
-when measurements were performed;
-minimum and maximum time to measurement.

A visit window of 6 to 13 weeks after ictus for the MoCA and EQ-5D-5L is considered acceptable. For the mRS score, a visit window of 10-15 weeks after ictus is considered acceptable.

## Outlier management

Outliers will be included.

## Statistical computer software

SPSS version 26.0 will be used.

## Format tables and graphs

#### Dummy Tables

Table 1. Baseline characteristics

|  | Eculizumab | Care as usual |
| --- | --- | --- |
| Total patients randomized | N (%) | N (%) |
| Age (years) | Mean/median (SD/IQR) | Mean/median (SD/IQR) |
| Female (%) | N (%) | N (%) |
| PAASH  1 (GCS 15)  2 (GCS 11-14)  3 (GCS 8-10)  4 (GCS 4-7)  5 (GCS 3) | N (%) N (%) N (%) N (%) N (%) | N (%) N (%) N (%) N (%) N (%) |
| Loss of consciousness during ictus | N (%) | N (%) |
| Hijdra sum score on admission head-CT | Median (IQR) | Median (IQR) |
| Pre-SAH mRS score 0 1 | N (%) N (%) | N (%) N (%) |
| Smoking  Current smoker Past smoker Never smoker | N (%) N (%) N (%) | N (%) N (%) N (%) |
| History of hypertension | N (%) | N (%) |
| Location of the aneurysm  Anterior  Posterior | N (%) N (%) | N (%) N (%) |
| Size of the ruptured aneurysm | Mean/median (SD/IQR) | Mean/median (SD/IQR) |
| Aneurysm treatment modality  Endovascular  Neurosurgical  No treatment | N (%) N (%) N (%) | N (%) N (%) N (%) |
| CSF withdrawal  Lumbar puncture EVD | N (%) N (%) | N (%) N (%) |

PAASH= Prognosis on Admission of Aneurysmal Subarachnoid Hemorrhage; GCS= Glasgow Coma Scale; mRS=modified Rankin Scale; SD=standard deviation; IQR=interquartile range.

Table 2. Primary and secondary outcomes

|  | N | Eculizumab | N | Care as usual | | P-value |
| --- | --- | --- | --- | --- | --- | --- |
| Primary outcome | | | | | | |
| CSF C5a concentration in pg/ml | N | Mean (95 CI)/Median (IQR) | N | Mean (95 CI)/Median (IQR) | |  |
| Secondary clinical outcomes | | | | | | |
| NIHSS score | N | Median (IQR) | N | | Median (IQR) |  |
| WFNS score | N | Median (IQR) | N | | Median (IQR) |  |
| MoCA score | N | Median (IQR) | N | | Median (IQR) |  |
| EQ-5D-5L | N | Median (IQR) | N | | Median (IQR) |  |
| EQ-VAS | N | Mean (95 CI)/Median (IQR) | N | | Mean (95 CI)/Median (IQR) |  |
| mRS score  1  2  3  4  5  6 | N | Median (IQR)  N (%)  N (%)  N (%)  N (%)  N (%)  N (%) | N | | Median (IQR)  N (%)  N (%)  N (%)  N (%)  N (%)  N (%) |  |

CSF= cerebrospinal fluid; NIHSS= National Institutes of Health Stroke Scale; WFNS= World Federation of Neurosurgical Societies; MoCA= Montreal Cognitive Assessment; EQ-5D-5L: EuroQol 5-dimensions 5-levels; EQ-VAS= EuroQol Visual Analogue Scale mRS= modified Rankin Scale; DCI= delayed cerebral ischemia; CI=confidence interval; IQR=interquartile range.

**Table 3. Secondary CSF outcomes**

|  | N | Eculizumab | N | Care as usual | P-value |
| --- | --- | --- | --- | --- | --- |
| Secondary CSF outcomes | | | | |  |
| IL-1B pg/ml | N | Mean (95 CI)/Median (IQR) | N | Mean (95 CI)/Median (IQR) |  |
| IL-6 pg/ml | N | Mean (95 CI)/Median (IQR) | N | Mean (95 CI)/Median (IQR) |  |
| IL-10 pg/ml | N | Mean (95 CI)/Median (IQR) | N | Mean (95 CI)/Median (IQR) |  |
| IL-18 pg/ml | N | Mean (95 CI)/Median (IQR) | N | Mean (95 CI)/Median (IQR) |  |
| MCP-1 pg/ml | N | Mean (95 CI)/Median (IQR) | N | Mean (95 CI)/Median (IQR) |  |
| sICAM pg/ml | N | Mean (95 CI)/Median (IQR) | N | Mean (95 CI)/Median (IQR) |  |
| sVCAM pg/ml | N | Mean (95 CI)/Median (IQR) | N | Mean (95 CI)/Median (IQR) |  |
| P-selectin pg/ml | N | Mean (95 CI)/Median (IQR) | N | Mean (95 CI)/Median (IQR) |  |
| E-selectin pg/ml | N | Mean (95 CI)/Median (IQR) | N | Mean (95 CI)/Median (IQR) |  |
| TNF-a pg/ml | N | Mean (95 CI)/Median (IQR) | N | Mean (95 CI)/Median (IQR) |  |
| MIF pg/ml | N | Mean (95 CI)/Median (IQR) | N | Mean (95 CI)/Median (IQR) |  |
| sC5b-9 AE/ml | N | Mean (95 CI)/Median (IQR) | N | Mean (95 CI)/Median (IQR) |  |
| CRP mg/L | N | Mean (95 CI)/Median (IQR) | N | Mean (95 CI)/Median (IQR) |  |
| sCD163 pg/ml | N | Mean (95 CI)/Median (IQR) | N | Mean (95 CI)/Median (IQR) |  |

CSF= cerebrospinal fluid; CI=confidence interval; IQR=interquartile range.

**Table 4. Secondary serum outcomes**

| Day | N | Eculizumab | N | Care as usual | P-value |
| --- | --- | --- | --- | --- | --- |
| Day 1 | N | Mean (95 CI)/Median (IQR) | N | Mean (95 CI)/Median (IQR) |  |
| Day 2 | N | Mean (95 CI)/Median (IQR) | N | Mean (95 CI)/Median (IQR) |  |
| Day 4 | N | Mean (95 CI)/Median (IQR) | N | Mean (95 CI)/Median (IQR) |  |
| Day 6 | N | Mean (95 CI)/Median (IQR) | N | Mean (95 CI)/Median (IQR) |  |
| Day 9 | N | Mean (95 CI)/Median (IQR) | N | Mean (95 CI)/Median (IQR) |  |
| Day 12 | N | Mean (95 CI)/Median (IQR) | N | Mean (95 CI)/Median (IQR) |  |
| Day 14 | N | Mean (95 CI)/Median (IQR) | N | Mean (95 CI)/Median (IQR) |  |

CI=confidence interval; IQR=interquartile range.

Table 5. Adverse events

|  | Eculizumab  No of patients  (%) | Care as usual  No of patients (%) |
| --- | --- | --- |
| Any AE | N (%) | N (%) |
| Most frequently reported AEs in either group  Nausea  X  X | N (%)  N (%)  N (%)  N (%) | N (%)  N (%)  N (%)  N (%) |
| Any SAE | N (%) | N (%) |
| Most frequently reported SAEs in either group  X  X  X | N (%)  N (%)  N (%) | N (%)  N (%)  N (%) |
| SAE possibly or probably related to eculizumab as determined by the investigators  X | N (%) | N (%) |
| Death | N (%) | N (%) |
| Infections during (S)AE follow-up  Meningitis, drain-related  Bacteremia  Pneumonia  Upper respiratory tract infection | N (%)  N (%)  N (%)  N (%)  N (%) | N (%)  N (%)  N (%)  N (%)  N (%) |
| Any suspected unexpected serious adverse reaction | N (%) | N (%) |

AE= adverse event; (S)AE= serious adverse event; DCI=delayed cerebral ischemia; CT=computed tomography.

#### Dummy Graphs

####


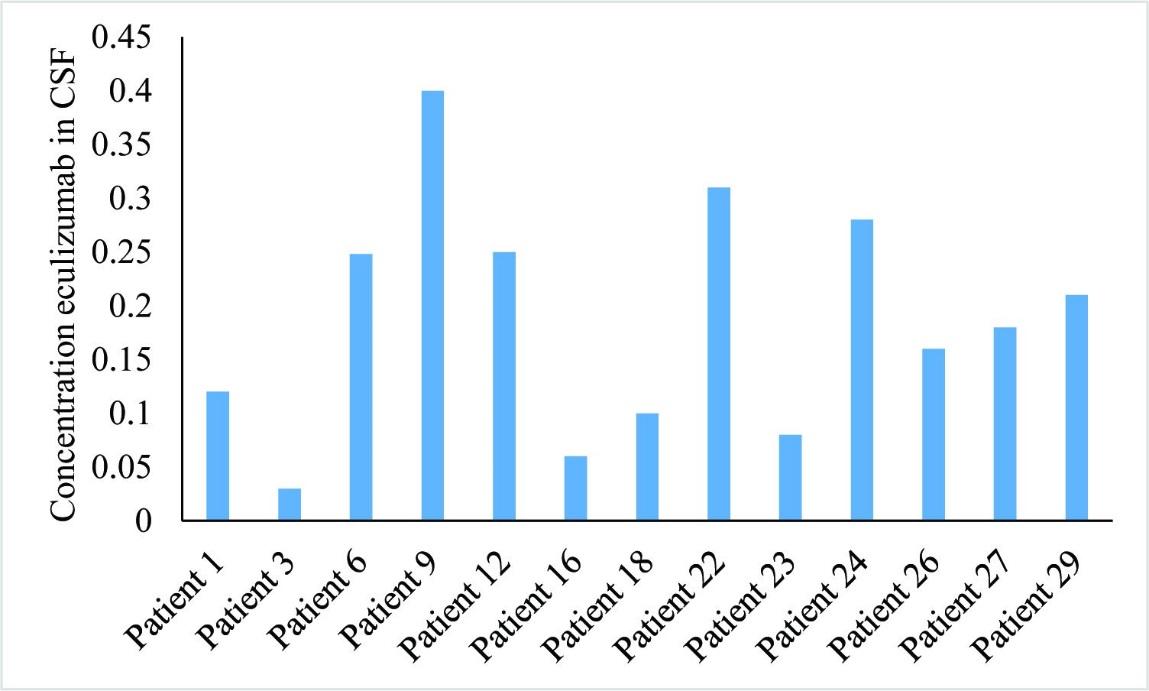


aSAH= aneurysmal subarachnoid hemorrhage; CSF= cerebrospinal fluid; mRS=modified Rankin Scale.

# **Definition of population for analysis**

## Subjects flowchart


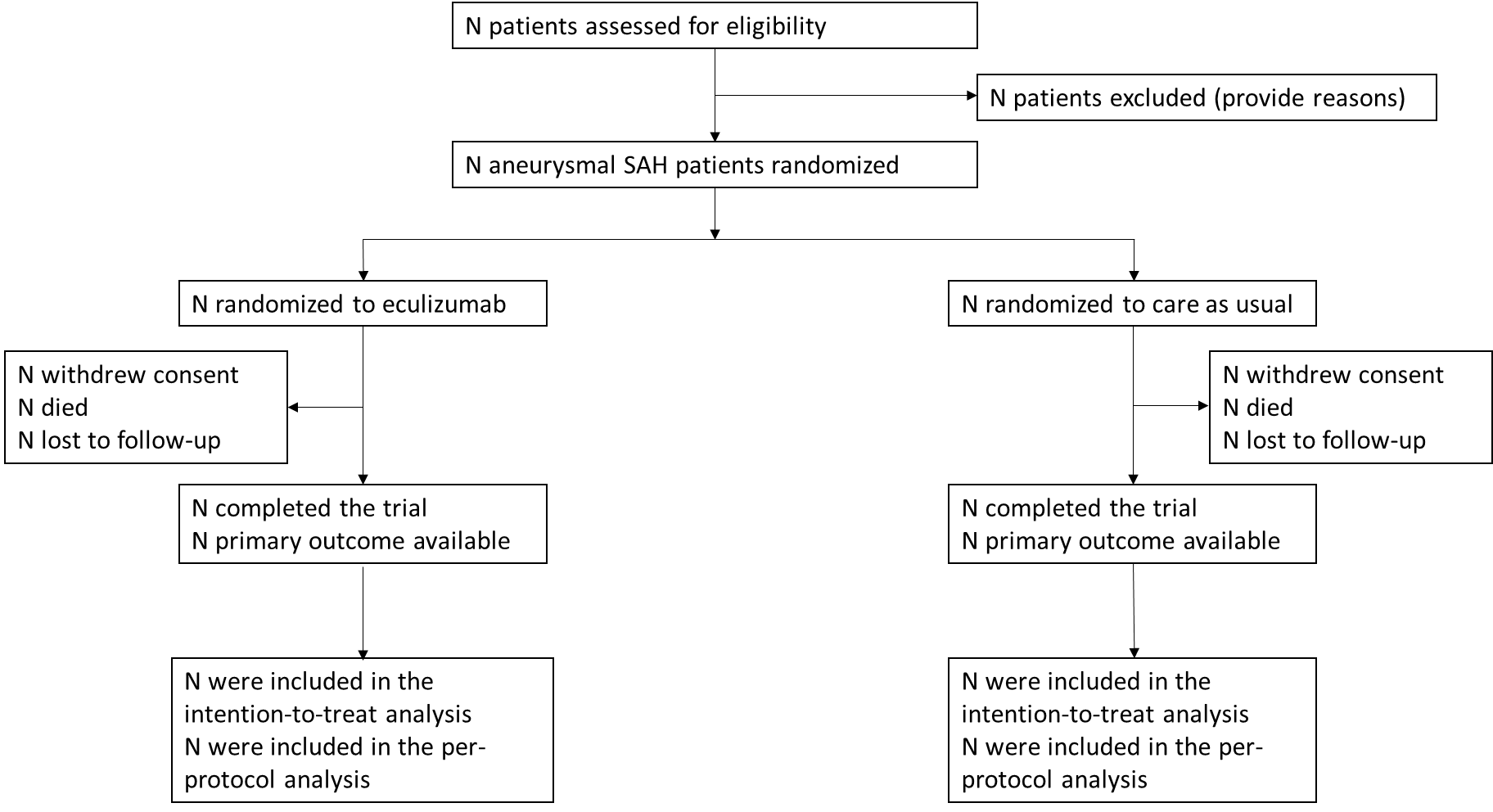


## Subject Data Sets

PP: Patients with CSF assessments who completed the study according to protocol up until CSF withdrawal.

ITT: All randomized patients with CSF assessments will be included according to treatment allocation, irrespective of whether or not they received the study drug.

A sensitivity analysis for the primary outcome will be performed. The details of the sensitivity analysis are described in section 8.2.

# **Statistical analysis**

## Descriptive statistics

Baseline characteristics will be summarized using descriptive statistics and presented according to treatment allocation (Table 1). Baseline characteristics include age, sex, PAASH score,^15^ loss of consciousness during ictus, Hijdra sum score on admission head-CT,^16^ pre-SAH mRS score, smoking status, history of hypertension, location of the aneurysm, size of the ruptured aneurysm, aneurysm treatment modality, time to randomization, time to aneurysm treatment, and CSF withdrawal. Mean with SD and medians with IQRs will be reported. Categorical data will be presented as proportions or rates. Normality of data will be explored by a Q-Q plot.

## Analysis of primary outcome

- Hypothesis

We expect the C5a concentration in the CSF to be decreased with 55% in the intervention group compared to the care as usual group.

- statistical approach

Means with corresponding 95% confidence intervals (CIs) or medians with interquartile ranges (IQRs) will be calculated for the intervention and control group. An independent t-test will be performed in case of a parametric distribution and a Mann-Whitney U-test in case of a non-parametric distribution. PP will be performed as primary analysis and ITT as supportive analysis.

- model assumptions check

Normality of data will be checked with Q-Q plots.

- sensitivity analysis

A sensitivity analysis will be performed for the PAASH score^15^ and Hijdra score.^16^ Both will be incorporated in an analysis of covariance (ANCOVA) with the PAASH and Hijdra score as covariates, intervention as the independent variable, and C5a concentration as the dependent variable. If convergence is not possible with a model that includes both the PAASH and Hijdra score, 2 separate models will be constructed: one with the PAASH score and one with the Hijdra score. If C5a concentration in CSF has a nonparametric distribution, an ANCOVA with rank transformation will be used.

## Analysis of secondary outcome

- hypothesis

Inflammatory parameters in serum and CSF are decreased in the intervention group compared to the care as usual group. NIHSS and WFNS are lower in the intervention group than in the care as usual group. In addition, MoCA, EQ-5D-5L, and mRS scores are lower in the intervention group compared to the care as usual group.

- statistical approach

Inflammatory parameters in serum (7 time points per patient): A repeated measurement model with treatment and time as independent variables and outcome will be constructed. If such a model cannot be constructed due to technical difficulties, means with 95% CI or medians with IQRs and independent t-tests or Mann-Whitney U-tests (depending on the distribution of the data) will be performed for each time point and measurement. The course of the parameters will also be presented graphically.

Inflammatory parameters in CSF (1 time point per patient): Means with 95% CI or medians with IQRs will be presented. An independent t-test or Mann-Whitney U-test will be performed for each parameter (depending on the distribution of the data).

Number of ischemic lesions on CT/MRI (1 time point per patient):
An independent t-test or Mann-Whitney U-test is performed (depending on the distribution of the data).

NIHSS score (1 time point per patient):
Medians with IQRs will be calculated and a Mann-Whitney U-test will be performed.

WFNS score (1 time point per patient):
Medians with IQRs will be calculated and a Mann-Whitney U-test will be performed.

MoCA (1 time point per patient):
Medians with IQRs will be calculated and a Mann-Whitney U-test will be performed.

EQ-5D-5L and EQ-VAS (1 timepoint per patient):
Crosswalk index values will be calculated for the EQ-5D-5L (<https://euroqol.org/eq-5d-instruments/eq-5d-5l-about/valuation-standard-value-sets/crosswalk-index-value-calculator/>). Means with 95% CI or medians with IQRs will be calculated and an independent t-test or Mann-Whitney U-test will be performed depending on the distribution of the data.

mRS score (1 time point per patient):
Medians with IQRs will be calculated and a Mann-Whitney U-test will be performed.

- model assumptions check

Normality of data will be checked with Q-Q plots.

- sensitivity analysis

No sensitivity analysis will be performed for the secondary outcome measures.

## Subgroup analysis

Not applicable.

# **SAFETY ANALYSIS**

AEs and SAEs will be categorized and presented as tabulated incidence rates (Table 4). Listings of AEs and SAEs will also be provided.

# **CHANGES TO THE PROTOCOL OR PREVIOUS VERSIONS OF SAP**

| Number | Changes made in SAP compared to research protocol |
| --- | --- |
| 1 | No stratification factors will be used. Erasmus MC did not include any patients. All patients are included at one center (UMCU). |
| 2 | WFNS and mRS scores will be analyzed with a Mann-Whitney U-test instead of a proportional odds model. |
|  |  |

# **REFERENCES**

1. Nieuwkamp DJ, Setz LE, Algra A, et al. Changes in case fatality of aneurysmal subarachnoid haemorrhage over time, according to age, sex, and region: a meta-analysis. *Lancet Neurol* 2009; 8: 635–42.

2. Johnston SC, Selvin S, Gress DR. The burden, trends, and demographics of mortality from subarachnoid hemorrhage. *Neurology* 1998; 50: 1413–8.

3. Rosengart AJ, Schultheiss KE, Tolentino J, et al. Prognostic factors for outcome in patients with aneurysmal subarachnoid hemorrhage. *Stroke* 2007; 38: 2315–2321.

4. Broderick JP, Brott TG, Duldner JE, et al. Initial and recurrent bleeding are the major causes of death following subarachnoid hemorrhage. *Stroke* 1994; 25: 1342–1347.

5. Macdonald RL. Delayed neurological deterioration after subarachnoid haemorrhage. *Nat Rev Neurol* 2013; 10: 44–58.

6. Vergouwen MDI, Jong-Tjien-Fa A V, Algra A, et al. Time trends in causes of death after aneurysmal subarachnoid hemorrhage: A hospital-based study. *Neurology* 2016; 86: 59–63.

7. Zanier ER, Zangari R, Munthe-Fog L, et al. Ficolin-3-mediated lectin complement pathway activation in patients with subarachnoid hemorrhage. *Neurology* 2014; 82: 126–34.

8. Mack WJ, Ducruet AF, Hickman ZL, et al. Early plasma complement C3a levels correlate with functional outcome after aneurysmal subarachnoid hemorrhage. *Neurosurgery* 2007; 61: 255–261.

9. Kasuya H, Shimizu T. Activated complement components C3a and C4a in cerebrospinal fluid and plasma following subarachnoid hemorrhage. *J Neurosurg* 1989; 71: 741–6.

10. Pellettieri L, Nilsson B, Carlsson CA, et al. Serum immunocomplexes in patients with subarachnoid hemorrhage. *Neurosurgery* 1986; 19: 767–71.

11. Yanamoto H, Kataoka H, Nakajo Y, et al. The role of the host defense system in the development of cerebral vasospasm: analogies between atherosclerosis and subarachnoid hemorrhage. *Eur Neurol* 2012; 68: 329–43.

12. Cai J-Y, Sun J, Yu Z-Q. Serum mannose-binding lectin levels after aneurysmal subarachnoid hemorrhage. *Acta Neurol Scand* 2016; 134: 360–367.

13. van Dijk BJ, Meijers JCM, Kloek AT, et al. Complement C5 Contributes to Brain Injury After Subarachnoid Hemorrhage. *Transl Stroke Res* 2020; 11: 678–688.

14. Pittock SJ, Lennon VA, McKeon A, et al. Eculizumab in AQP4-IgG-positive relapsing neuromyelitis optica spectrum disorders: An open-label pilot study. *Lancet Neurol* 2013; 12: 554–562.

15. Takagi K, Tamura A, Nakagomi T, et al. How should a subarachnoid hemorrhage grading scale be determined? A combinatorial approach based solely on the Glasgow Coma Scale. *J Neurosurg* 1999; 90: 680–687.

16. Hijdra A, Brouwers PJ, Vermeulen M, et al. Grading the amount of blood on computed tomograms after subarachnoid hemorrhage. *Stroke* 1990; 21: 1156–1161.
