# Supplementary material for: Safety and pharmacodynamic efficacy of eculizumab in aneurysmal subarachnoid hemorrhage (CLASH): A phase 2a randomized clinical trial
Source: Eur Stroke J. 2023 Aug 22;8(4):1097–106. doi: 10.1177/23969873231194123 (PMC10683736; doi:10.1177/23969873231194123)
Supplement: sj-pdf-3-eso-10.1177_23969873231194123 – Supplemental material for Safety and pharmacodynamic efficacy of eculizumab in aneurysmal subarachnoid hemorrhage (CLASH): A phase 2a randomized clinical trial [file sj-pdf-3-eso-10.1177_23969873231194123.pdf]

# CompLement C5 Antibodies for decreasing brain injury after aneurysmal Subarachnoid Hemorrhage

***safety and proof-of-concept***

# CLASH

**(July 2019)**

Version number: 11.0, date 08-07-2019

**PROTOCOL TITLE** *„**C**omp**L**ement C5 **A**ntibodies for decreasing brain injury after aneurysmal **S**ubarachnoid **H**emorrhage - safety and proof-of-concept”.*

| | |
|---|---|
| **Protocol ID** | NL63723.041.17 |
| **Short title** | CLASH |
| **EudraCT number** | 2017-004307-51 |
| **Version** | 11 |
| **Date** | July 8, 2019 |
| **Sub Investigator** | I. Koopman, MD<br>PhD candidate<br>Department of Neurology and Neurosurgery, Brain Center Rudolf Magnus, University Medical Center Utrecht. Matthias van Geuns building<br>Room: 02.15<br>Phone: 088-7571441<br>Email: I.Koopman-4@umcutrecht.nl |
| **Coordinating investigator/project leader** | Dr. M.D.I. Vergouwen, MD PhD<br>Neurologist<br>Department of Neurology and Neurosurgery, Brain Center Rudolf Magnus, University Medical Center Utrecht<br>Room: G3-228<br>Phone: 088 755 0455<br>Email: M.D.I.Vergouwen@umcutrecht.nl |
| **Principal investigator(s)**<br>***UMC Utrecht*** | Dr. M.D.I. Vergouwen, MD PhD<br>Neurologist<br>Department of Neurology and Neurosurgery, Brain Center Rudolf Magnus, University Medical Center Utrecht<br>Room: G3-228<br>Phone: 088 755 0455<br>Email: M.D.I.Vergouwen@umcutrecht.nl |
| ***Erasmus MC*** | Dr. M. van der Jagt, MD PhD |

| | |
|---|---|
| | Neurointensivist<br>Department of Intensive Care Medicine, Erasmus MC, Rotterdam<br>Room: H-611<br>Phone: 010-7030478<br>Email: m.vanderjagt@erasmusmc.nl |
| **Trial steering committee** | I. Koopman, MD, PhD candidate<br>M.D.I. Vergouwen, MD PhD, neurologist<br>M. van der Jagt, MD PhD, neurologist-intensivist<br>M. Bartels, MD PhD, pediatric hematologist/oncologist<br>J.P. Greving, PhD, clinical epidemiologist<br>Prof. A. Slooter, MD PhD, neurologist-intensivist<br>Prof. A. van der Zwan, MD PhD, neurosurgeon<br>Prof. G.J.E. Rinkel, MD FRCP(E), head of department and neurologist |
| **Sponsor** | University Medical Center Utrecht |
| **Subsidising party** | ZonMW (project number 95105015)<br>Alexion Pharmaceuticals (#100237) |
| **Independent expert (s)** | Prof. Dr. L.J. Kappelle,<br>Medical head of the cerebrovascular department/ neurologist<br>Department of Neurology and Neurosurgery, Brain Center Rudolf Magnus, University Medical Center Utrecht<br>Room: G.03.230<br>Phone: 088 75 579 75<br>Email: l.kappelle@umcutrecht.nl |
| **Data & Safety Monitoring Board** | Prof. Dr. J. Stam (chair)<br>Prof. Dr. D. van de Beek<br>Dr. H. F. Lingsma |
| **Laboratory sites** | Laboratory department<br>University Medical Center Utrecht<br>Heidelberglaan 100<br>3584 CX Utrecht |

| | |
|---|---|
| | The Netherlands<br>Room: G.03.330 (AZU, third floor)<br>Phone: 088 75 588 26/ 088 75 71771 (clinical trials)<br>Email: StudiesLKCH@umcutrecht.nl<br><br>Laboratory of Translational Immunology<br><br>University Medical Center Utrecht<br><br>Heidelberglaan 100<br><br>3584 CX Utrecht<br><br>The Netherlands<br><br>Laboratory department<br>Erasmus Medical Center<br>'s-Gravendijkwal 230<br>3015 CE Rotterdam<br>Netherlands<br><br>Sanquin<br>Plesmanlaan 125<br>1066 CX Amsterdam<br>The Netherlands |
| **Pharmacy** | Dr. B. Romberg<br>Pharmacist/clinical pharmacologist<br>Department Pharmacy, University Medical Center Utrecht<br>Division of Laboratory and Pharmacy<br>Room: D00.4.42<br>Phone: +31 (0)88 75 590 65<br>Email: B.Romberg@umcutrecht.nl |

## PROTOCOL SIGNATURE SHEET

Version number: 11.0, date 08-07-2019

**PROTOCOL SIGNATURE SHEET**

| Name | Signature | Date |
|---|---|---|
| Head of department:<br>Prof. dr. G.J.E. Rinkel, neurologist | [illegible] | 8 juli '19 |
| Project leader:<br>Dr. M.D.I. Vergouwen, neurologist | [illegible] | 8/7/19 |

## TABLE OF CONTENTS

1. INTRODUCTION AND RATIONALE ....12
2. OBJECTIVES ....14
3. STUDY DESIGN ....16
4. STUDY POPULATION ....22
 4.1 Population ....22
 4.2 Inclusion criteria ....22
 4.3 Exclusion criteria ....23
 4.4 Sample size calculation ....23
5. TREATMENT OF SUBJECTS ....24
 5.1 Investigational product/treatment ....24
 5.2 Use of co-intervention ....24
 5.3 Escape medication ....24
6. INVESTIGATIONAL PRODUCT ....25
 6.1 Name and description of investigational product ....25
 6.2 Summary of findings from non-clinical studies ....26
 6.3 Summary of findings from clinical studies ....26
 6.4 Summary of known and potential risks and benefits ....28
 6.5 Description and justification of route of administration and dosage ....29
 6.6 Dosages, dosage modifications and method of administration ....29
 6.7 Preparation and labelling of Investigational Medicinal Product ....29
 6.8 Drug accountability ....30
7. NON-INVESTIGATIONAL PRODUCT ....31
 7.1 Name and description of non-investigational products ....31
 7.2 Dosages, dosage modifications and method of administration ....31
 7.3 Preparation and labelling of Non Investigational Medicinal Product ....31
8. METHODS ....32
 8.1 Study parameters/endpoints ....32
  8.1.1 Main study parameter/endpoint ....32
  8.1.2 Other study parameters ....32
 8.2 Randomization, blinding and treatment allocation ....32
 8.3 Study procedures ....33
 8.4 Withdrawal of individual subjects ....38
  8.4.1 Specific criteria for withdrawal ....38
 8.5 Replacement of individual subjects after withdrawal ....39
 8.6 Follow-up of subjects withdrawn from treatment ....39
 8.7 Premature termination of the study ....39
9. SAFETY REPORTING ....41
 9.1 Temporary halt for reasons of subject safety ....41
 9.2 AEs, SAEs and SUSARs ....41
  9.2.1 Adverse events (AEs) ....41
  9.2.2 Serious adverse events (SAEs) ....41
  9.2.3 Recording and reporting of AEs and SAEs ....42

9.2.4 Suspected unexpected serious adverse reactions (SUSARs) ....43
9.3 Annual safety report ....44
9.4 Follow-up of adverse events....44
9.5 Data Safety Monitoring Board (DSMB) ....45
10. STATISTICAL ANALYSIS....46
10.1 Primary study parameters ....46
10.2 Secondary study parameters....46
10.3 Interim analysis ....47
11. ETHICAL CONSIDERATIONS....48
11.1 Regulation statement ....48
11.2 Recruitment and consent....48
11.3 Objection by minors or incapacitated subjects....50
11.4 Benefits and risks assessment, group relatedness ....51
11.5 Compensation for injury ....53
12. ADMINISTRATIVE ASPECTS, MONITORING AND PUBLICATION ....54
12.1 Handling and storage of data and documents ....54
12.2 Monitoring and Quality Assurance....55
12.3 Amendments....55
12.4 Annual progress report....55
12.5 Temporary halt and (prematurely) end of study report ....56
12.6 Public disclosure and publication policy....56
13. STRUCTURED RISK ANALYSIS....57
13.1 Potential issues of concern....57
13.2 Synthesis ....59
14. REFERENCES ....62
Appendix 1: Algorithm for treatment of anaphylaxis (in Dutch)....66
Appendix 2: Drug Accountability Form....67
Appendix 3: NIHSS score (in Dutch)....68
Appendix 4: WFNS score (in Dutch) ....75
Appendix 5: MoCA score (in Dutch)....76
Appendix 6: EQ-5D-5L questionnaire (in Dutch) ....77
Appendix 7: Questionnaire on AEs and SAEs (in Dutch) ....79
Appendix 8: mRS score (in Dutch)....81
Appendix 9: AE report form ....82
Appendix 10: SAE report form ....83

## LIST OF ABBREVIATIONS AND RELEVANT DEFINITIONS

| | |
|---|---|
| ABR | ABR form, General Assessment and Registration form, is the application form that is required for submission to the accredited Ethics Committee (In Dutch, ABR = Algemene Beoordeling en Registratie) |
| ADC | Apparent Diffusion Coefficient |
| AE | Adverse Event |
| aHUS | Atypical Hemolytic Uremic Syndrome |
| AR | Adverse Reaction |
| CA | Competent Authority |
| CCMO | Central Committee on Research Involving Human Subjects; in Dutch: Centrale Commissie Mensgebonden Onderzoek |
| CRP | C-reactive protein |
| CT | Computed tomography |
| CSF | Cerebrospinal fluid |
| CV | Curriculum Vitae |
| DSMB | Data Safety Monitoring Board |
| DWI | Diffusion Weighted Imaging |
| EMA | European Medicine Agency |
| EQ-5D-5L | EuroQol 5-dimensions 5-levels |
| Erasmus MC | Erasmus Medical Center |
| EU | European Union |
| EudraCT | European drug regulatory affairs Clinical Trials |
| FDA | Food and Drug Administration |
| FLAIR | Fluid-Attenuated Inversion Recovery |
| GCP | Good Clinical Practice |
| GCS | Glasgow Coma Scale |
| IB | Investigator’s Brochure |
| IC | Informed Consent |
| IMP | Investigational Medicinal Product |
| IMPD | Investigational Medicinal Product Dossier |
| ICU | Intensive Care Unit |
| MAC | Membrane attack complex |
| MCU | Medium Care Unit |
| HCU | High Care Unit |

| | |
|---|---|
| **Hijdra score** | **A score to assess the amount of blood after subarachnoid haemorrhage on computed tomography** |
| **MedDRA** | **Medical Dictionary for Regulatory Activities** |
| **METC** | **Medical research ethics committee (MREC); in Dutch: medisch ethische toetsing commissie (METC)** |
| **MoCA** | **Montreal Cognitive Assessment** |
| **MRI** | **Magnetic Resonance Imaging** |
| **mRS** | **modified Rankin Scale** |
| **NIHSS** | **National Institutes of Health Stroke Scale** |
| **PNH** | **Paroxysmal Nocturnal Hemoglobinuria** |
| **PROBE** | **Prospective Randomized Open Blinded End-point** |
| **QoL** | **Quality of life** |
| **(S)AE** | **(Serious) Adverse Event** |
| **SAH** | **Subarachnoid haemorrhage** |
| **SNP** | **Single nucleotide polymorphism** |
| **SPC** | **Summary of Product Characteristics (in Dutch: officiële productinformatie IB1-tekst)** |
| **Sponsor** | **The sponsor is the party that commissions the organisation or performance of the research, for example a pharmaceutical company, academic hospital, scientific organisation or investigator. A party that provides funding for a study but does not commission it is not regarded as the sponsor, but referred to as a subsidising party.** |
| **SUSAR** | **Suspected Unexpected Serious Adverse Reaction** |
| **UMCU** | **University Medical Center Utrecht** |
| **Wbp** | **Personal Data Protection Act (in Dutch: Wet Bescherming Persoonsgegevens)** |
| **WFNS** | **World Federation of Neurosurgical Societies** |
| **WMO** | **Medical Research Involving Human Subjects Act (in Dutch: Wet Medisch-wetenschappelijk Onderzoek met Mensen** |

**SUMMARY**

**Rationale:** Complement activation is associated with brain injury after aneurysmal subarachnoid hemorrhage (SAH). Treatment with eculizumab (complement factor C5 antibodies) may be a novel treatment option to decrease brain injury and improve prognosis in patients with aneurysmal SAH.

**Objective**: To investigate the biological effect (proof-of-concept) and safety of eculizumab in patients with aneurysmal SAH.

**Study design:** This study will be a bi-center, open-label, randomized phase II trial with blinded outcome assessment (Prospective Randomized Open Blinded End-point, PROBE) in patients with aneurysmal SAH.

**Study population:** In this study, 40 patients with aneurysmal SAH admitted to the University Medical Center Utrecht (UMCU) or Erasmus Medical Center Rotterdam (Erasmus MC) will be included (20 patients in the intervention group and 20 patients in the control group). Patients with aneurysmal SAH are eligible if admitted to one of the participating hospitals within ≤11.5 hours of ictus and with a World Federation of Neurosurgical Societies (WFNS) score of 1-5 on admission are eligible.

**Intervention:** Patients will receive either intravenous infusion with eculizumab (C5 antibodies) in addition to standard treatment for aneurysmal SAH (intervention group) or standard treatment for aneurysmal SAH (control group). Eculizumab will be administered within 12 hours after ictus and on day 3 and day 7 at a dose of 1200 mg for each infusion.

**Main study parameters/endpoints:** The primary outcome measure is C5a concentration in cerebrospinal fluid (CSF) 48-72 hours after ictus. Secondary outcome measures include the occurrence of adverse and serious adverse events (AEs and SAEs), blood and CSF parameters of inflammation, quality of life (QoL), functional and cognitive outcomes, and the presence and volume of cerebral infarction. Blinded outcome assessment will be performed for C5a, modified Rankin Score (mRS) and infections.

**Nature and extent of the burden and risks associated with participation, benefit and group relatedness:**

Eculizumab has been approved for treatment of patients with Paroxysmal Nocturnal Hemoglobinuria (PNH) and Atypical Hemolytic Uremic Syndrome (aHUS). Recently, refractory generalized myasthenia gravis in patients who are anti-acetylcholine receptor (AChR) antibody-positive has been approved as a new indication by the EMA. The most common AE reported during treatment with eculizumab is headache (mostly in the initial phase). Among meningococcal infections, the most common SAE was meningococcal sepsis. Due to its working mechanism, patients who are treated with eculizumab are at increased risk of infections, specifically infections with the meningococcus (*Neisseria meningitidis*) bacteria. To address the increased risk of meningococcal infection, patients in

the intervention group will receive prophylactic treatment with ciprofloxacin during the first four weeks after ictus. In addition, throat and rectal swaps will be performed weekly in the intervention group during in-hospital stay to test for yeast or fungus carriership/colonization and multi-drug resistance (BRMO, bijzonder resistente micro-organismen). Patients in the intervention group with a central line or an external lumbar or ventricular drain and a positive yeast or fungal culture will also receive antifungal prophylaxis. If the multi-drug resistance culture is positive, the microbiologist will be consulted and prophylactic treatment with ciprofloxacin will be switched to a different prophylactic antibiotic that sufficiently covers the increased risk of infection. Patients in the intervention group will be provided with safety instructions and a patient safety card before discharge. These patients will be asked to carry the patient safety card on their person until three months after the last infusion with the eculizumab. Complications of CSF withdrawal from an external ventricular or lumbar drain include a possible increased risk of infection. For a lumbar puncture, possible risks consist of: 1) post-lumbar puncture headache; 2) back pain; and 3) radicular pain or numbness. Very rare complications of a lumbar puncture include: infection, bleeding, and abducens palsy. Withdrawal of blood poses a very minor risk of possible infection at the injection site. There is no increased risk associated for any of the other examinations performed (e.g. daily neurological examination, brain MRI without gadolinium, cognitive-, QoL-, and mRS testing). Since early brain injury (brain injury <72 hours) is possibly reversible, treatment with eculizumab is started as soon as possible but not later than 12 hours. Potential benefits include a decrease in brain injury and hereby a better prognosis.

# 1. INTRODUCTION AND RATIONALE

Aneurysmal subarachnoid hemorrhage (SAH) is a subtype of stroke with a median age of onset of 55 years.[1] Although SAH is less common than ischemic stroke, the loss of productive life years after SAH is similar to that after ischemic stroke, which is due to the young age of SAH patients and its poor prognosis.[2] Important determinants of poor functional outcome after SAH are early brain injury (brain injury <72 hours after ictus) and delayed cerebral ischemia (4-14 days after the bleeding).[3,4] No treatment exists to reduce early brain injury and the effects of current strategies (nimodipine, euvolemia) to prevent delayed cerebral ischemia are only modest.[5] With the current treatment standards, approximately 1/3 of all patients with aneurysmal SAH dies within 90 days, 1/3 remains dependent, and 1/3 has no or minor impairments.[6] Because of this poor prognosis, new treatment options are needed to reduce brain injury and improve prognosis.

The inflammatory response is considered to play a key role in the pathogenesis of early brain injury and delayed cerebral ischemia after aneurysmal SAH. Previous studies found that the complement cascade is activated in patients with SAH and associated with poor functional outcome.[7–12] Several studies were performed to investigate if: 1) previously observed associations between complement activation and outcome after SAH also imply causal relationships; and 2) C5 antibodies can potentially reduce brain injury. These studies include autopsy studies, cerebrospinal fluid (CSF) studies, genetic studies, and animal studies and their results show that:[13]

1) Brains of patients who died from aneurysmal SAH had much higher complement expression than brains from controls. This difference was most pronounced in areas with brain infarction *(Figure 1)*;
2) C5a concentrations in CSF were markedly increased (>1400-fold increase)) shortly after SAH compared with controls, and decreased over the first 14 days after ictus *(Figure 2)*;
3) Plasma levels of C5a correlated with a C5 single nucleotide polymorphism (SNP) in a Dutch cohort of 930 patients with SAH. In this cohort, the C5 SNP also correlated with functional outcome 3 months after ictus;
4) In a SAH mouse model, brain injury was >40% reduced in C5a receptor knock-out mice and in wildtype mice treated with C5 antibodies compared with untreated wildtype mice *(Figure 3)*.

**Figure 1. Results from autopsy study.**

| | SAH, infarcted area | SAH, non-infarcted area | Control | Comparisons* | |
|---|---|---|---|---|---|
| | | | | Infarcted vs. non-infarcted | Infarcted vs. control |
| C1q | 0.21 (0.19-0.23) | 0.17 (0.16-0.17) | 0.14 (0.13-0.15) | $p<0.001$ | $p<0.001$ |
| C3c | 0.21 (0.20-0.24) | 0.17 (0.17-0.17) | 0.14 (0.13-0.15) | $p<0.001$ | $p<0.001$ |

Values represent median optical density values with IQR. * = analyzed with Mann-Whitney U test

**Figure 2. Complement component C5a concentrations in cerebrospinal fluid (CSF) after subarachnoid hemorrhage.**

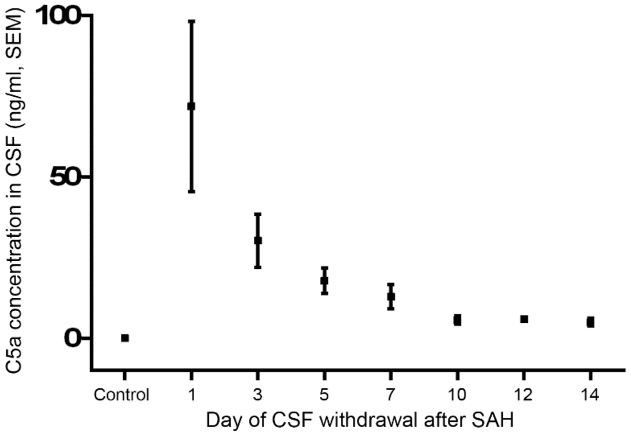

**Figure 3. Results from animal studies**

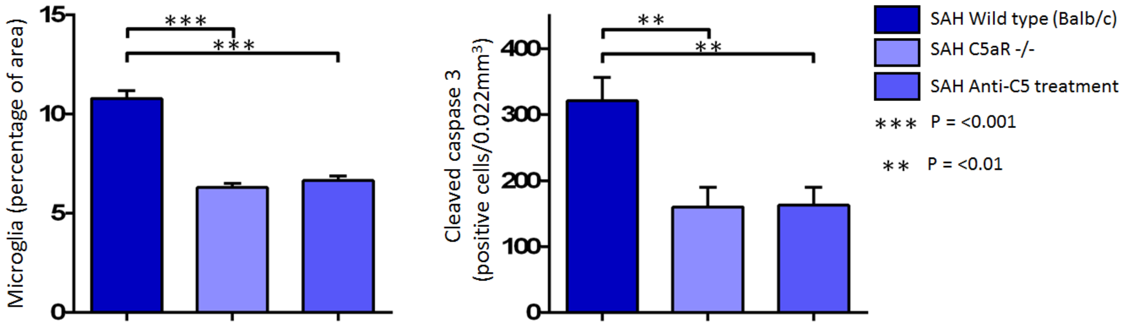


Quantified microglia activation (percentage of area) and apoptosis after subarachnoid hemorrhage in wild type mice (n=14), C5aR-/- mice (n=15) and C5a antibody treated mice (n=15).

From the aforementioned results, we concluded that complement activation is not only associated with brain injury after SAH, but that treatment with C5 antibodies also decreases brain injury in an SAH animal model. Although C5 antibodies (eculizumab) are currently available as a treatment option for patients with Paroxysmal Nocturnal Hemoglobinuria (PNH) and Atypical Hemolytic Uremic Syndrome (aHUS), eculizumab has not yet been administered to patients with aneurysmal SAH. In the current trial, we will investigate the biological effect and safety of eculizumab in patients with aneurysmal SAH.

## 2. OBJECTIVES

This study aims to investigate the biological effect and safety of eculizumab in patients with aneurysmal SAH.

Primary objective and outcome measure

To assess the effect of eculizumab on the C5a concentration in the CSF of patients with aneurysmal SAH. The primary outcome measure will be C5a concentration in CSF. CSF will be obtained between 48-72 hours after ictus before study drug administration on day 3.

Secondary objectives and outcome measures

To assess the safety of eculizumab in patients with aneurysmal SAH and to assess the effect of eculizumab on parameters of inflammation, quality of life (QoL), imaging, functional and cognitive outcomes. The secondary outcome measures are the occurrence of adverse events (AEs) and serious adverse events (SAEs), blood and CSF parameters of inflammation, quality of life (QoL), functional, and cognitive outcomes, and the presence and volume of cerebral infarction. AEs and SAEs are categorized according to the terms of the

Medical Dictionary for Regulatory Activities (MedDRA). AEs and SAEs will be reported up to four weeks after ictus. QoL and functional outcome will be measured by the EQ-5D-5L questionnaire and the mRS. Cognitive functioning will be assessed with the use of the Montreal Cognitive Assessment (MoCA).

## 3. STUDY DESIGN

This will be a bi-center, open-label, randomized, phase II clinical trial with blinded outcome assessment (PROBE). Informed consent will be obtained from the patient or a legally acceptable surrogate. If the patient was incapacitated on admission, the patient will be asked for informed consent as soon as the patient is capable of giving informed consent (see section 11.2 *"recruitment and consent"*). If patients are eligible and consent is obtained, randomization starts. Depending on the outcome of randomization ('eculizumab' or 'care as usual'), either eculizumab will be administered in addition to standard SAH care or only standard SAH care will be given. The intervention group will receive three intravenous eculizumab infusions at different time points *(Figure 4.)* Previous studies show that the C5a concentration in CSF is extremely elevated in the first two weeks after aneurysmal SAH.[13] Therefore, it was decided to repeat drug administration at day 3 and day 7 to prevent wash-out of eculizumab. The eculizumab dose will be 1200 mg per infusion, similar to the dose used in patients with aHUS. Patients in the intervention group will receive prophylactic treatment with ciprofloxacin during the first four weeks after ictus. Throat and rectal swaps will be performed weekly in the intervention group during in-hospital stay to test for yeast or fungal carriership/colonization and multi-drug resistance (BRMO, bijzonder resistente micro-organismen). Patients in the intervention group with a central line or an external lumbar or ventricular drain and a positive yeast or fungal culture will also receive antifungal prophylaxis (*Figure 5.).* Throat and rectal swaps are performed weekly in all patients in the intervention group to make sure that the culture results are available in case the patient requires a central line or drain at a later moment. If the multi-drug resistance culture is positive, the microbiologist will be consulted and prophylactic treatment with ciprofloxacin will be switched to a different prophylactic antibiotic that sufficiently covers the increased risk of infection.

These patients will also be provided with safety instructions and a patient safety card before discharge. Patients in the intervention group will be asked to carry the patient safety card on their person until three months after the last infusion with the study drug. Patient in the control group will **not** receive prophylactic antibiotics, safety instructions or a safety card. Most of the study procedures will be performed during hospital stay, since aneurysmal SAH patients are usually admitted to the hospital for at least 14 days.

During hospital stay the following patient characteristics will be collected: 1) date and time of ictus; 2) date and time of admission; 3) site of inclusion; 4) date and time of inclusion; 5) date and time of study drug administration (three times eculizumab); 6) date and time of CSF collection; 7) age at inclusion; 8) sex; 9) duration of loss of consciousness during ictus; 10) clinical condition on admission (WFNS score); 11) mRS score before SAH; 12) daily

Glasgow Coma Score (GCS) during the first 14 days after ictus; 13) WFNS- and National Institutes of Health Stroke Scale (NIHSS) score 14 days after ictus 14) smoking status; 15) history of hypertension; 16) aneurysm size and location; 17) blood volume on admission head computed tomography (CT) according to the Hijdra score; 18) aneurysm treatment modality (endovascular or microneurosurgical clipping); 19) date and time of aneurysm treatment; 20) occurrence of rebleeding; 21) date and time of rebleeding; 22) occurrence of delayed cerebral ischemia; 23) date and time of delayed cerebral ischemia; 24) bacterial infections of various causes (e.g. pneumonia, urinary tract infection and meningitis/ventriculitis); 25) date and time of infection; 26) the presence and volume of cerebral infarction on brain magnetic resonance imaging (MRI). Time will be denoted in hours and minutes. Rebleeding will be defined as 1) a sudden clinical deterioration with increased hemorrhage found either on the CT-scan compared with previous CT imaging or at autopsy; or 2) or acute hemodynamic instability with fresh blood in the lumbar or ventricular drain.

Clinical deterioration due to delayed cerebral ischemia will be defined as a clinical deterioration (new focal deficits, decreased GCS of ≥2 points or both) persisting 1 hour or longer in which other causes have been excluded.[14] These other causes include hydrocephalus, rebleeding, seizures, hypoxia, hypotension, fever, effect of sedatives and hyponatremia. Cerebral infarction due to delayed cerebral ischemia is defined as the presence of cerebral infarction on head CT or MRI obtained within 6 weeks after ictus or proven at autopsy.[14] Cerebral infarction will not be regarded as delayed cerebral ischemia when infarction was already present on CT or MRI 24-48 hours after aneurysm treatment or if the infarction is possibly caused by ventricular drainage. Infections will be defined according to the previously established international criteria from the Centers for Disease Control and Prevention.[15]

The head CT on admission will be evaluated by the local investigator to determine the Hijdra score. The head CT performed on admission at the UMCU or Erasmus MC will be used, unless there is *only* a head CT-scan from the referring hospital available. The WFNS score will be determined based on clinical data collected as part of standard clinical practice.

Various measurements will be performed during hospital stay and clinical outpatient follow-up (see section 8.3 *“study procedures”* and *Figure 6*). Blood will be withdrawn on admission and on day 2, 4, 6, 9, 12, and 14. CSF will be obtained a single time between 48-72 hours after ictus, if possible from an external ventricular or lumbar drain and otherwise a lumbar puncture will be performed. CSF sampling will always be performed before study drug

administration on day 3. Daily neurological examination by the GCS will be performed during the first fourteen days of hospital stay. The GCS performed in the morning (between 08:00-12:00) will be used in case there are multiple GCS measurements. On day 14 after ictus, a neurological examination will be performed to assess the NIHSS and WFNS scores. At discharge when the patient is in a stable condition, a brain MRI will be performed to evaluate the extent and total volume of cerebral infarction. Four weeks after ictus (+/- one week) a questionnaire on possible AEs or SAEs will be sent to the patient or treating physician unless the required follow-up of AEs and SAEs was already performed in the hospital. Ten weeks after ictus (+/- two weeks), cognitive functioning and QoL will be assessed by the Montreal Cognitive Assessment (MoCA) and EQ-5D-5L. At thirteen weeks after ictus (+/- two weeks) mRs will be determined during telephone consultation. We expect that the majority of our patients will be admitted to the hospital for longer than 14 days. In that case, possible AEs and SAEs will be monitored during in-hospital stay and if necessary in combination with the questionnaire for the remaining days after discharge.

<u>Study duration</u>

The estimated study duration is two and a half years (patient inclusion 2 years).

<u>Study setting</u>

This study will be conducted at two tertiary referral hospitals: the University Medical Center Utrecht (UMCU) and the Erasmus University Medical Center (Erasmus MC). At the UMCU, patients are admitted for at least 24 hours to the intensive care unit (ICU) or to the medium care unit (MCU). At the Erasmus MC, patients are admitted to the intensive care unit (ICU) or to the neurological/neurosurgical high care unit (HCU). Patients will stay on the neurological/neurosurgical HCU for the first 3 days. The first infusion of eculizumab can be administered on either the emergency department, the neurological/neurosurgical MCU, neurological/neurosurgical HCU , or the ICU. During hospital stay, patients can be transferred to the neurology or neurosurgery ward.

**Figure 4. Study design CLASH trial**

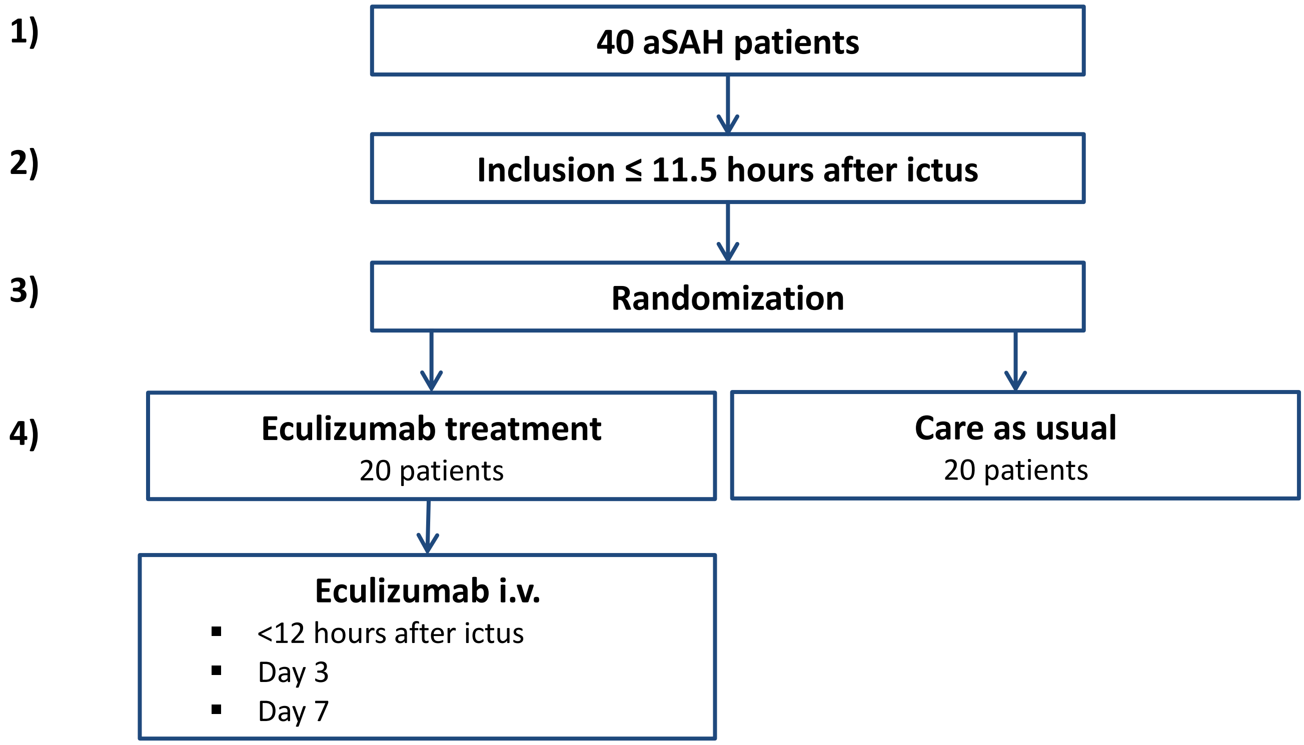


1) In total, 40 patients will be included in this study. We expect a 50/50 inclusion rate at the UMCU and Erasmus MC. More information about the inclusion rate can be found on page 21.
2) Patients with a WFNS score of 1-5 on admission are eligible for this study. Written informed consent will be obtained from the patient or a legally acceptable surrogate. If the patient was incapacitated on admission, the patients will be asked for informed consent as soon as they are capable of giving informed consent.
3) If informed consent is obtained, patients will be allocated to the intervention or control group by randomization.
4) In case of allocation to the 'eculizumab treatment' (intervention group), the first intravenous infusion of eculizumab is administered as soon as possible but at the latest within 12 hours of the ictus.
5) Measurements are performed according to the time schedule depicted in *Figure 6* for both the intervention and control group.

**Figure 5. Prophylactic treatment CLASH trial**

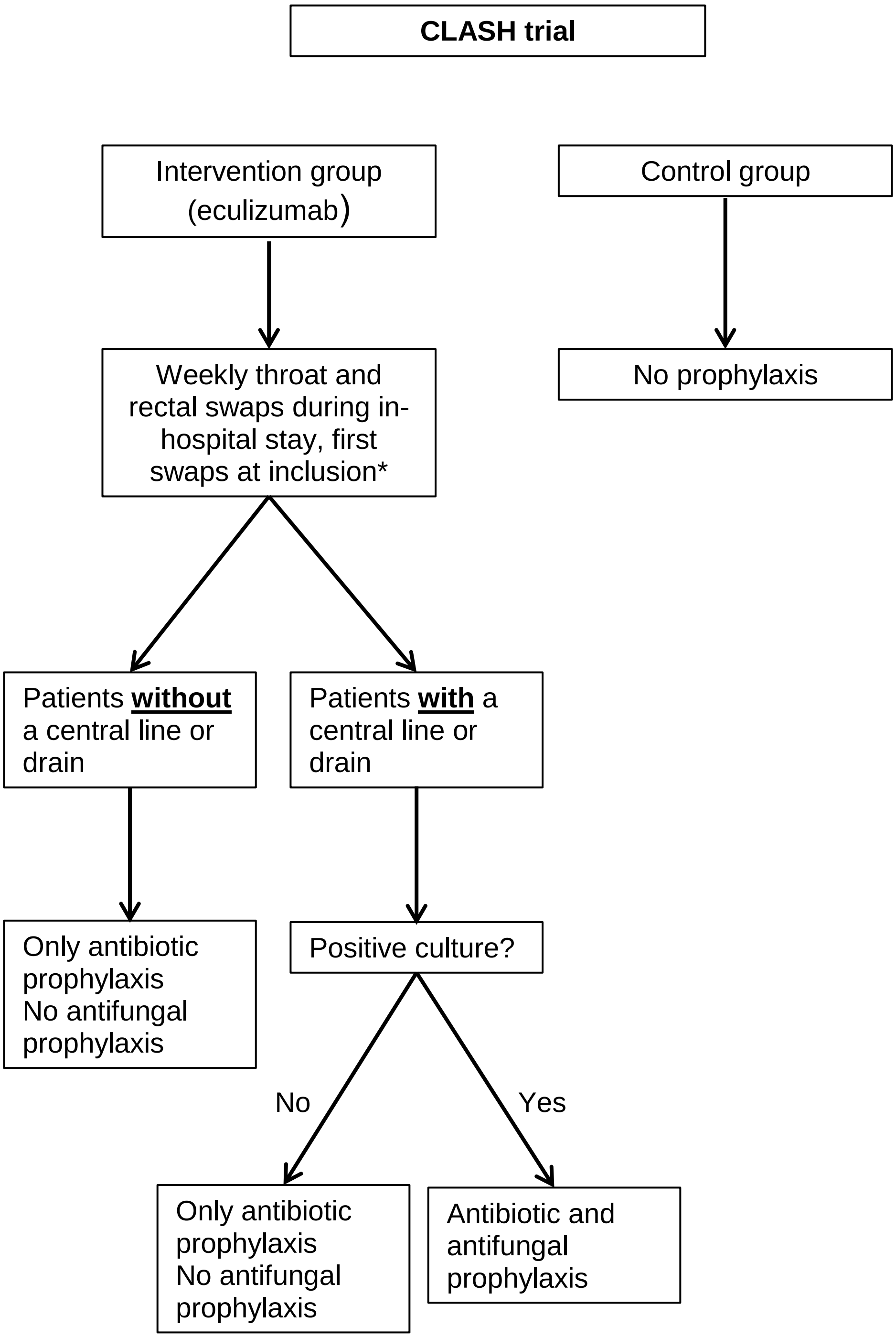


*Throat and rectal swaps will be tested for yeast carriership/colonization and multi-drug resistance (BRMO, bijzonder resistente micro-organismen). If the multi-drug resistance culture is positive, the microbiologist will be consulted and prophylactic treatment with ciprofloxacin will be switched to a different prophylactic antibiotic that sufficiently covers the increased risk of infection.

## Figure 6. Measurements CLASH trial

Day →

| Measurements | 1 | 2 | 3 | 4 | 5 | 6 | 7 | 8 | 9 | 10 | 11 | 12 | 13 | 14 | discharge | 4 weeks† | 10 weeks‡ | 13 weeks‡ |
|---|---|---|---|---|---|---|---|---|---|---|---|---|---|---|---|---|---|---|
| Blood withdrawal | ▲ | ▲ | | ▲ | | ▲ | | | ▲ | | | ▲ | | ▲ | | | | |
| CSF sample | | | ▲* | | | | | | | | | | | | | | | |
| Neurological examination | ▲ | ▲ | ▲ | ▲ | ▲ | ▲ | ▲ | ▲ | ▲ | ▲ | ▲ | ▲ | ▲ | ▲ | | | | |
| WFNS score | | | | | | | | | | | | | | ▲ | | | | |
| NIHSS score | | | | | | | | | | | | | | ▲ | | | | |
| Brain MRI | | | | | | | | | | | | | | | ▲ | | | |
| Questionnaire AEs and SAEs | | | | | | | | | | | | | | | | ▲ | | |
| MoCA | | | | | | | | | | | | | | | | | ▲ | |
| EQ-5D-5L | | | | | | | | | | | | | | | | | ▲ | |
| Modified Rankin Score | | | | | | | | | | | | | | | | | | ▲ |

*CSF will be obtained between 48 -72 hours.
† +/- one week
‡ +/- two weeks

Legends
CSF= cerebrospinal fluid; WFNS= World Federation of Neurosurgical Societies; NIHSS= National Institutes of Health Stroke Scale; MRI= Magnetic Resonance Imaging; MoCA= Montreal Cognitive Assessment; EQ-5D-5L= Standardized instrument for use as a measure of health outcome; AEs= adverse events and; SAEs= serious adverse events.

# 4. STUDY POPULATION

## 4.1 Population

In total, 40 patients will be recruited from two tertiary referral hospitals: the UMCU and the Erasmus MC. We expect that approximately half of the patients will be included at the UMCU and half of the patients at the Erasmus MC. However, inclusion rate might differ between both hospitals in which case the 50/50 distribution of participants at each hospital could slightly vary. Aneurysmal SAH has an incidence of 9 per 100.000 person years.[16] The number of annual admissions of patients with aneurysmal SAH is approximately 150 at the UMCU and 100 at the Erasmus MC. Currently, the UMCU participates in a multicenter trial which includes patients with aneurysmal SAH. This is the ULTRA trial (http://www.ultrastudie.nl; NTR3272; NL20120125 CCMO), in which patients need to be included within 24 hours after ictus. Over the last year, 40 patients from the UMCU were included in the ULTRA trial. Several medical centers who refer patients with aneurysmal SAH to the UMCU do not participate in the ULTRA trial, which leaves room for other trials. Another trial in patients with aneurysmal SAH is expected to start patient inclusion in October 2017 (ASH trial: EudraCT number 2016-005151-25). The ASH trial aims to include 40 patients within 2 years. Patients need to be included within 72 hours and patients who are on the intensive care >72 hours are excluded. The ASH trial will only include patients who have their aneurysm coiled (patients with neurosurgical treatment of the ruptured aneurysm will not be included). Since the CLASH trial has stricter inclusion criteria than the ULTRA and ASH trials, the CLASH trial will receive priority for patient inclusion. Based on a recruitment rate of 30% and the assumption that 50 aneurysmal SAH patients each year are eligible for inclusion in the trial, it will be feasible to include 20 patients during the two year inclusion period at the UMCU. To increase recruitment rate, Erasmus MC will also participate in the CLASH trial and recruit 20 patients. There are currently no clinical trials conducted with aneurysmal SAH patients at the Erasmus MC.

## 4.2 Inclusion criteria

In order to be eligible for this study, a subject must meet all of the following criteria:

1) Confirmed aneurysmal SAH;

*Definition:* The presence of subarachnoid blood on the brain CT and visualization of an aneurysm on either CT angiography, magnetic resonance (MR) angiography or digital subtraction angiography with a compatible bleeding pattern.

2) Admission to either the UMCU or Erasmus MC within 11.5 hours after ictus;

3) Age 18 years and older.

### 4.3 Exclusion criteria

A potential subject who meets any of the following criteria will be excluded from participation in this study:

1) Life expectancy < 10 days;
2) Pregnant or breast-feeding women;
3) Participation in another clinical therapeutic study;
4) History of splenectomy or asplenia (potentially increased risk of meningococcal infection);
5) Hematologic malignancy;
6) Patients receiving chemotherapy;
7) Patients who will undergo or underwent an organ transplantation;
8) Patients with myasthenia gravis, glucose-6-phosphate dehydrogenase (G6PD) deficiency, or tuberculosis;
9) Patients who are or will be treated by plasmapheresis or hemodialysis;
10) Patient with a creatinine clearance of <30 or serum creatinine levels of >169 µmol/l
11) Patients with a known hereditary complement deficiency;
12) Patients allergic to eculizumab, proteins derived from mouse products or other monoclonal antibodies;
13) Patients allergic to (prophylactic) antibiotic treatment for *Neisseria meningitidis* (quinolones or ceftriaxone (therapeutic));
14) If on admission, it is likely that the aneurysm can only be treated with extracranial-intracranial bypass surgery;
15) If based on head imaging, it will be unlikely that CSF can be obtained 48-72 hours after ictus;
16) Patients with an ongoing infection on admission which is not appropriately treated;
17) Patients who were treated >4 times with antibiotics during the last year;
18) Patients on immunosuppressive therapy.

### 4.4 Sample size calculation

Based on a previous study with eculizumab in patients with neuromyelitis optica, we expect a difference in C5 concentration in CSF of 55% between groups with and without treatment with C5 antibodies.[17] With a standard deviation of 50%, 5% type I error, and 80% power, we need 13 patients per group (www.clincalc.com/Stats/SampleSize.aspx). The group size will be increased to 20 patients per group, taking into account an assumed mortality rate of 25% and 2 patients per group who refuse a lumbar puncture in a later phase despite giving informed consent earlier.

# 5. TREATMENT OF SUBJECTS

## 5.1 Investigational product/treatment

Eculizumab (SOLIRIS®) is a humanized monoclonal antibody directed against complement factor C5.[18] Current therapeutic indications for eculizumab include treatment of patients with PNH and aHUS.[19] Recently, refractory generalized myasthenia gravis in patients who are anti-acetylcholine receptor (AChR) antibody-positive has been approved as a new indication by the EMA. Dosage regimen for adults with PNH consists of 600 mg weekly intravenous infusions in the induction phase (4 weeks) and 900 mg two-weekly intravenous infusions in the maintenance phase. For patients with aHUS, the induction phase (4 weeks) consists of 900 mg of weekly intravenous infusions followed by a maintenance phase of 1200 mg intravenous infusions every two weeks. A *“summary of product characteristics (SPC) for eculizumab”* is provided in“*D2b. SPC Eculizumab*”.

## 5.2 Use of co-intervention

Patients will receive standard treatment for aneurysmal SAH. Aneurysm treatment consists of endovascular or microneurosurgical treatment. Standard medical treatment includes nimodipine (60 mg every four hours until day 21 after ictus) and euvolemia (daily fluid intake approximately 3 L until day 14 after ictus). Female patients with childbearing potential will be asked to use effective contraceptives up to five months after the last infusion with the study drug.

## 5.3 Escape medication

| Symptoms | Escape medication |
|---|---|
| Headache | Standard pain medication after aneurysmal SAH at the UMCU (in the order specified below, and no concomitant use unless specified below):<br>1. Acetaminophen 1000 mg each dose with a maximum amount of 4000 mg a day.<br>2. Tramadol 100 mg each dose with a maximum amount of 400 mg a day.<br>3. Piritramide 10 mg each dose with a maximum amount of 40 mg a day.<br>Acetaminophen can be given concomitantly with either tramadol or piritramide. |

| | |
|---|---|
| | Standard pain medication after aneurysmal SAH at the Erasmus MC (in the order specified below, and no concomitant use unless specified below):<br>1. Acetaminophen 1000 mg each dose with a maximum amount of 4000 mg a day.<br>2. Morphine 5-10 mg each dose (subcutaneous injection) six times a day or morphine 20 mg (oral administration) two times a day.<br>Acetaminophen can be given concomitantly with morphine. |
| Nausea | 1.Metoclopramide/domperidone 10 mg each dose with a maximum amount of 30 mg a day.<br>2. Ondansetron 4 mg each dose with a maximum amount of 16 mg |
| Infection | Infections will be treated according to the UMCU guidelines for antibiotic treatment (http://umcu.swabid.nl) or the Erasmus MC guidelines for antibiotic treatment (http://erasmusmc.swabid.nl). |
| Anaphylaxis | Anaphylaxis will be treated both at the UMCU and Erasmus MC according to the treatment algorithm for anaphylaxis from “Het Acute Boekje” (appendix 1).[20] |

# 6. INVESTIGATIONAL PRODUCT

## 6.1 Name and description of investigational product

Eculizumab (SOLIRIS®) is a humanized monoclonal antibody directed against complement factor C5.[18] The usage for eculizumab in the CLASH study does not correspond with the current indications. The dose used in the CLASH study is similar to the dose used for aHUS patients.

### 6.2 Summary of findings from non-clinical studies

See investigator's brochure section "*Non-clinical studies*" on page 19.

### 6.3 Summary of findings from clinical studies

A detailed description of the findings from clinical studies can be found in the investigator's brochure section "*Effect in humans*" on pages 20-35.

<u>Safety and clinical efficacy in patients with PNH</u>

A total of twelve clinical studies have been completed and one noninterventional postmarketing study is ongoing in patients with PNH. An overview of these studies can be found in the investigator's brochure "*Appendix B*" on page 58. A summary of the TRIUMPH and SHEPHERD study, both phase III trials published in internationally renowned peer-reviewed journals is presented below.[21,22]

In patients with PNH, intravenous infusion with eculizumab decreased intravascular hemolysis by 86% and reduced the need for transfusion of packed red blood cells.[22] None of the SAEs (4 in eculizumab group and 9 in control group) was regarded as a consequence of eculizumab treatment.[22] A subsequent trial with a follow-up of 52-weeks found similar results regarding a decrease in hemolytic activity.[21] In total, 44 SAEs were reported of which none were considered as probable or definitely related to treatment with eculizumab. Six SAEs were regarded as possibly related to treatment with eculizumab. These included pyrexia (2), abdominal distension (1), viral infection (1), anxiety (1), adrenal impairment (1). The most common AEs reported include headache, nasopharyngitis and upper respiratory tract infections. Two AEs were regarded as caused by eculizumab treatment: 1) dysgeusia; and 2) mild hematoma. One patient discontinued eculizumab treatment due to an AE not caused by eculizumab treatment. This patient subsequently developed a thrombotic complication which was considered unrelated to eculizumab treatment. The patient eventually died due to complications also unrelated to eculizumab treatment. The proportion of patients with an infection in the eculizumab treatment group at 26-weeks was comparable to the placebo-treated patients of the aforementioned study (70.8% versus 77.3%).[22,19]

<u>Safety and efficacy in patients with aHUS</u>

A total of seven clinical studies have been completed and 3 noninterventional postmarketing studies are ongoing in patients with aHUS. A summary of the results of a multicenter phase II trial conducted on adolescent and adult aHUS patients is presented below.[23] For more detailed information about the results of clinical studies conducted on pediatric patients with aHUS, see the investigator's brochure "*Appendix B*" on page 62.

A trial with two substudies was conducted in patients with aHUS.[23] In both substudies, patients received regular intravenous eculizumab infusions. Results showed a decrease in platelet count and normalized hematological values comparing baseline values before and after treatment.[23] No SAEs were regarded as definitely caused by eculizumab treatment. Three SAEs were deemed as possibly due to treatment with eculizumab. These included hypertension (in a patient with a history of hypertension), peritonitis and influenza. All possible or probably SAEs recovered without discontinuation of eculizumab treatment. Patients in both substudies showed significant inhibition of complement activity maintained during the entire study period.

Safety and efficacy in patients with refractory generalized myasthenia gravis

A total of two studies have been completed in patients with refractory generalized myasthenia gravis and one extension study is ongoing.[24] A summary of the results of a phase II trial is presented below. [24] The results of the phase III trial (not yet published) can be found in the investigator's brochure "*Effect in humans*" on page 26.

Patients with refractory generalized myasthenia gravis were randomized to one of the two treatment sequences with eculizumab. Primary outcome measure was the quantitative myasthenia gravis score. The results showed a decrease on the quantitative myasthenia gravis score but this was not statically significant.[24] However, since this was a phase II study, the study was not powered to assess clinical endpoints. No SAEs were reported and the most common AEs included nausea, back pain, nasopharyngitis and headache.

Safety and efficacy in patients with neuromyelitis optica

One study has been completed and one study is ongoing in patients with neuromyelitis optica. The results of the study that has been completed and published are presented below.[17]

An open-label trial with eculizumab as a treatment for patients with aquaporin-4 (AQP4)-IgG-positive relapsing neuromyelitis showed a decreased number of relapses with eculizumab treatment. Headache was the most reported AE. One patient developed a meningococcal sepsis and sterile meningitis two months after the start of treatment with eculizumab. Treatment with eculizumab was discontinued and the patient fully recovered with the appropriate treatment. After recovery, eculizumab treatment was restarted in the aforementioned patient. One patient developed joint swelling in her fingers (a similar episode

was also reported 3 years earlier while the patient was not treated with eculizumab) and another patients had a transient ischemic attack (>50 packyear smoking history).

Safety and efficacy in sensitized renal transplant recipients
One study has been terminated and one study is ongoing in patients with an antibody-mediated rejection (AMR) after renal transplantation.
The study that has been terminated was a phase II trial to evaluate safety and efficacy of eculizumab to prevent AMR in sensitized recipients of living donor kidney. Primary outcome consisted of occurrence of biopsy proven AMR, graft loss, patient death or loss to follow-up at week 9. The primary endpoint did not reach statistical significance and leading to termination of the study.

In the aforementioned studies eculizumab was administered at a dosage of 600, 900 and 1200 mg with dosing regimen depending on the indication. All patients were vaccinated with a meningococcal vaccine two weeks in advance and received prophylactics antibiotics if treatment with eculizumab was started immediately. Patients were also instructed to carry the patent safety card on their person.

### 6.4 Summary of known and potential risks and benefits

The most common reported AE was headache (mostly at the initial phase). Among meningococcal infections the most common SAE was meningitis. The rate of meningococcal infection in Alexion-sponsored clinical studies is reported in the investigator's brochure on page 48. Since headache and nausea occur in most patients with aneurysmal SAH, standard care in these patients consists of pharmaceutical treatment of headache and nausea. Due to its working mechanism, patients who are treated with eculizumab are at increased risk of infections, specifically infections with the meningococcus (*Neisseria meningitidis*) bacteria. Thirty percent of the patients with aneurysmal SAH have infections during hospital admission.[25] Therefore, physicians and nurses already closely monitor SAH patients for possible symptoms or signs of infections. The question remains if the risk, type and severity of infection are influenced by treatment with eculizumab in patients with aneurysmal SAH. Patients in the intervention group will receive prophylactic ciprofloxacin to reduce the chance of infection with *Neisseria meningitidis*. They will also receive antifungal prophylaxis if the patient has a central line or an external lumbar or ventricular drain and a positive yeast or fungal culture. In addition, these patients will receive safety instructions and a patient safety card before discharge. A detailed list of all AEs and SAEs reported can be found in *D2b. SPC Eculizumab*". Potential benefits from treatment with eculizumab include a

decrease in brain injury (from early brain injury and delayed cerebral ischemia) and hereby an improved prognosis.

### 6.5 Description and justification of route of administration and dosage

In previously conducted studies, intravenous administration of 1200 mg was considered safe and this dose is approved by the FDA and EMA as maintenance therapy for patients with aHUS.[19,23,26] The study drug will be administered by intravenous infusion via an infusion pump in 35 minutes. The route of administration and dosage correspond with the current guidelines for patients with aHUS. The high dose and repeat drug administration on day 3 and day 7 was chosen to prevent a wash-out effect because C5a levels are extremely elevated (>800-fold) in the first two weeks after SAH.[13]

### 6.6 Dosages, dosage modifications and method of administration

Eculizumab will be administered at a dose of 1200 mg for each infusion (<12 hours of ictus, day 3 and day 7).

### 6.7 Preparation and labelling of Investigational Medicinal Product

The investigational product will be provided by Alexion pharmaceuticals in vials of 30 ml with either 300 mg (10mg/ml) of eculizumab. The following instructions apply for the preparation of the eculizumab (in concordance with the manufacturer's instructions):

<u>Instructions for dilution</u>

1. Withdraw the total amount of 4 vials of eculizumab with a sterile syringe.
2. Transfer the amount into an infusion bag.
3. Dilute the solution with 120 ml NaCl 0,9%. The solution should be clear and colorless.
4. Gently agitate the infusion bag containing the diluted solution to ensure thorough mixing of the product and diluent.
5. The diluted solution should be allowed to warm to room temperature prior to administration by exposure to ambient air.

After dilution, the product should be used immediately. However, chemical and physical stability of eculizumab has been shown for 24 hours if stored in a refrigerator between 2°C and 8°C. Eculizumab will be prepared by a nurse at the emergency department, ICU, neurological/neurosurgical HCU, neurological/neurosurgical MCU, or neurology/neurosurgery ward.

### 6.8 Drug accountability

Eculizumab will be provided by Alexion pharmaceuticals. The pharmacist will perform digital drug accountability on stock level (e.g. registration of incoming eculizumab). Alexion will perform the labelling of eculizumab . The pharmacist will deliver the eculizumab vials to the emergency medicine department, ICU, neurological/neurosurgical MCU, or neurology/neurosurgery ward (UMCU) and the emergency medicine department, ICU, neurological/neurosurgical HCU, or neurology/neurosurgery ward (Erasmus MC). The eculizumab vials will be stored in a refrigerator at 2°C – 8°C either at the ICU, neurological/neurosurgical HCU, neurological/neurosurgical MCU, or neurology/neurosurgery ward. The study drug will be stored at a locked area with restricted access, separately from other medication and out of reach and sight of children. Accountability logs will be used for drug accountability at the emergency department, ICU, neurological/neurosurgical HCU, neurological/neurosurgical MCU, or neurology/neurosurgery ward (see appendix 2). The accountability logs will be filled out by either the treating physician, investigator or nurse. The investigator will regularly check the supplies of the eculizumab to verify the correctness of the drug accountability logs. At the end of the trial all unused study drug medication will be destroyed in accordance with Alexion's instructions with certification of such destruction provided to Alexion.

# 7. NON-INVESTIGATIONAL PRODUCT

## 7.1 Name and description of non-investigational products

Ciprofloxacin will be used as prophylactic antibiotic for the intervention group to reduce the risk of meningococcal infection. Fluconazole will be used as a prophylactic antifungal therapy for patients who have a central line or an external lumbar or ventricular drain and a positive yeast or fungal culture.

## 7.2 Dosages, dosage modifications and method of administration

Prophylactic ciprofloxacin will be administered in a dose of 750 mg two times a day for four weeks after ictus to the intervention group. If renal function is decreased with a creatinine clearance of 30-60 ml/min/1,73 $m^2$ or serum creatinine levels of 124-168 µmol/l, the dosage will be adjusted to 500 mg two times a day for four weeks. In case oral administration is not possible, ciprofloxacin can be administered intravenously.  Ciprofloxacin (two times 750 mg a day) is recommended as a prophylactic antibiotic in the guidelines for paroxysmal nocturnal hemoglobinuria for patients with PNH.

If prophylactic fluconazole therapy is necessary, an induction dose of 400 mg is administered followed by a maintenance dose of 200 mg daily for four weeks after ictus. If renal function is decreased below ≤ 50 ml/min, the maintenance dose is decreased to 100 mg daily. In case oral administration is not possible, fluconazole can be administered intravenously.

## 7.3 Preparation and labelling of Non Investigational Medicinal Product

In case ciprofloxacin or fluconazole will be  administered as infusion, both will be prepared according to the manufacturer’s instructions. Labelling will be performed by the pharmacy.

# 8. METHODS

## 8.1 Study parameters/endpoints

### 8.1.1 Main study parameter/endpoint

1) C5a concentration in CSF 48-72 hours after ictus;

### 8.1.2 Other study parameters

2) Occurrence of AEs and SAEs;
3) Blood and CSF parameters of inflammation (e.g. CRP and cytokines);
4) Concentration of eculizumab in blood and CSF;
5) Daily neurological condition measured by GCS during the first fourteen days of hospital stay;
6) Neurological condition measured by the NIHSS and WFNS score 14 days after ictus;
7) Cerebral infarction on brain MRI at hospital discharge;

*Definition:* Cerebral infarction due to delayed cerebral ischemia is defined as the presence of cerebral infarction on brain CT or MRI obtained within 6 weeks after ictus or proven at autopsy. Cerebral infarction will not be regarded as delayed cerebral ischemia when infarction was already present on CT or MRI 24-48 hours after aneurysm treatment or when the infarction is possibly caused by ventricular drainage.[14]

8) Cognition and QoL ten weeks (+/- two weeks) after ictus;
9) The mRS thirteen weeks (+/- two weeks) after ictus.

## 8.2 Randomization, blinding and treatment allocation

<u>8.2.1 Randomization</u>

After informed consent from the patient or a legally acceptable surrogate is obtained, the treating physician or investigator can start randomization through an online tool. Randomization will be stratified according to study site. Subsequently, the patient will be allocated to one of the following two groups: 1) eculizumab treatment in addition to standard aneurysmal SAH care (intervention group); or 2) standard aneurysmal SAH care (control group). The Julius Center will develop a digital randomization list and design an online tool for randomisation (accessible via a website). The eculizumab vials will be labelled with MedID numbers according to a list provided by the Julius Center. Completion of the randomization procedure results in the generation of ‘eculizumab’ or ‘care as usual’. In case of eculizumab, a MedID number can be entered when the allocation is ‘eculizumab’ in order to track which vials are given to which patient. The Julius Center will provide a list with the MedID numbers to Alexion who will be responsible for labelling of the medication.

8.2.2 Blinding

This is an open-label study with blinded outcome assessment (PROBE). The following outcomes will be blinded: C5a measurements in the CSF, mRS score, and infections. The laboratory analyst and person who will perform the mRS score will be blinded to treatment allocation. An expert panel will be established to determine if an infection occurred during the follow-up period. The expert panel will be blinded to treatment allocation and base their decision on patient-reports, clinical history and if available laboratory or radiological assessments.

8.2.3 Treatment allocation

Patients presented to the UMCU or Erasmus MC with symptoms indicative of a subarachnoid hemorrhage will undergo a head CT and head CT angiography (either at the UMCU, Erasmus MC, or at the referring hospital). Patients or legally acceptable surrogates from eligible patients admitted to the UMCU or Erasmus MC within 11.5 hours after ictus are asked for informed consent. Upon obtaining informed consent, the treating physician or investigator will perform online randomization with either ‘eculizumab’ or ‘care as usual’ as an outcome. In case randomization results in ‘eculizumab’, the treating physician instructs the nurse to prepare the eculizumab vials with the correct MedID and start administration of eculizumab (intravenous infusion) at the emergency department, ICU, neurological/neurosurgical HCU, or neurological/neurosurgical MCU. Infusion with eculizumab has to be started as soon as possible but at the latest within 12 hours after ictus. The treating physician or investigator will ask for informed consent (see section 11.2 *“recruitment and consent”* for more details on the consent procedure).

### 8.3 Study procedures

SAH treatment is based on the SAH treatment protocol, which includes standard prevention and treatment of complications after aneurysmal SAH. Similar protocols are used in the UMCU and Erasmus MC. The subarachnoid hemorrhage treatment protocols from the UMCU and Erasmus MC protocols are based on recommendations from the Dutch guidelines on aneurysmal SAH, the guidelines from the American Heart Association/American Stroke Association, and the European Stroke Organisation.[27,28]

Treatment protocol

*Treatment procedures that are part of standard SAH treatment:*

Patients with aneurysmal SAH receive nimodipine therapy (60 mg six times a day) for three weeks. Patients will be kept euvolemic (daily fluid intake approximately 3 L) for two weeks.

Patients admitted to the ICU receive selective digestive decontamination (SDD) and selective oropharyngeal decontamination (SOD) for the first four days.

*Treatment procedures that are extra for this study:*
All patients in the intervention group will have to undergo weekly throat and rectal swaps during in-hospital stay to test for yeast carriership/colonization and multi-drug resistance (BRMO, bijzonder resistente micro-organismen). Patients in the intervention group will receive prophylactic treatment with ciprofloxacin during the first four weeks after ictus. Patients in the intervention group with a central line or an external lumbar or ventricular drain and a positive yeast or fungal culture will also receive antifungal prophylaxis. If the multi-drug resistance culture is positive, the microbiologist will be consulted and prophylactic treatment with ciprofloxacin will be switched to a different prophylactic antibiotic that sufficiently covers the increased risk of infection In case the patient in the intervention group is admitted to the ICU and received SDD/SOD, prophylactic antibiotics will be started after SDD/SOD is discontinued and administered up until the first four weeks after ictus. Patients in the intervention group will receive three eculizumab infusions at different time points (≤ 12 hours of ictus, day 3, and day 7) in addition to standard SAH treatment. Patients in the control group will receive standard SAH treatment and will **not** receive prophylactic antibiotics/antifungal therapy, safety instructions or a safety card. Infusion of eculizumab occurs through an already present peripheral venous catheter (conform standard SAH protocol). The first eculizumab infusion will be either at the emergency department, ICU, neurological/neurosurgical HCU, or neurological/neurosurgical MCU where continuous monitoring is present. Depending on the clinical situation, the subsequent eculizumab infusions will be either at the ICU, neurological/neurosurgical HCU, neurological/neurosurgical MCU, or neurological/neurosurgical ward. Patients will be on either the ICU, HCU, or MCU for at least the first 24 hours after admission and most likely for a longer period of time since patients with a WFNS score 1-5 are included in this study. All patients will be under close monitoring at least for the first 24 hours after the first infusion. If an anaphylactic shock would occur in the intervention group, this will be noted immediately and direct actions can be taken. Previous studies, however, did not describe any patients experiencing an anaphylactic shock.[19,26] Current practice at the UMCU in patients with PNH and aHUS who receive their first eculizumab infusion is that the patients are observed for an hour after which they are allowed to go home. This observation time is not considered necessary during subsequent infusions in patients with PNH. Those patients can go home after the infusion with eculizumab if they do not experience any side-effects.

<u>Blood samples</u>

*Measurements that are part of standard SAH treatment:*
Blood samples are withdrawn by venipuncture during the first ten days to monitor sodium and potassium levels and if necessary other laboratory measurements are also monitored.

*Measurements that are extra for this study:*
Blood samples will be collected on admission and on day 2, 4, 6, 9, 12, and 14 after ictus. Since blood withdrawal for the first ten days is part of standard SAH care, the extra burden for participants will be the amount of blood that needs to be withdrawn and two times extra blood withdrawal on days 12 and 14. Standard SAH care entails that one blood collection tube of 3.5 ml is withdrawn for sodium and potassium levels during the first ten days. For this study, two extra blood collection tubes need to be withdrawn on admission and on days 2, 4, 6 and 9. Thus, in total three collection tubes (each 3.5 ml) are withdrawn on the aforementioned time points: 1) one for sodium, potassium (standard SAH care) and C-reactive protein (CRP) measurements; 2) one for complement pathway measurements and cytokine measurements; and 3) one for C5b-9 and eculizumab concentration measurements. Three collection tubes with blood (each 3.5 ml) need to be withdrawn on days 12 and 14 for the following measurements: 1) CRP; 2) complement pathway- and cytokines measurements; 3) C5b-9 and eculizumab concentration. Processing and storage methods of the samples depend on the analysis that has to be performed. C5b-9 and eculizumab measurements will be kept on ice, centrifuged and frozen at -80 °C for batch analysis. The risks of venipuncture include pain, a bruise at the point where the blood is withdrawn, redness and swelling of the vein, infection, and a rare risk of fainting.

<u>CSF samples</u>
*Measurements that are part of standard SAH treatment:*
In case a patient develops a clinically symptomatic hydrocephalus, the patient will be treated by CSF drainage as part of standard SAH care. Depending on several factors, the clinical team will decide to either perform CSF drainage by a (repeated) lumbar puncture or placement of an external ventricular or lumbar drain. An external ventricular drain is chosen if the third or fourth ventricle or the cerebral aqueduct is filled with blood due to the SAH.

*Measurements that are extra for this study:*
In case an external ventricular or lumbar drain is present, CSF will be obtained from the drain 48-72 hours after ictus. This procedure will be performed by the investigator. Prior to the start of the study, the investigator will receive a short training from the neurosurgical resident/neurosurgeon on how to perform CSF sampling from a drain. One CSF collection tube needs to be filled with CSF for the following measurements: C5a, C5b-9, cytokines,

CRP, and eculizumab concentration measurements. The CSF collection tube has to be filled with 8 ml of CSF. CSF samples for cytokine and eculizumab measurements will be kept on ice, centrifuged and frozen at −80 °C for batch analysis. The local laboratory site will store all CSF samples until study inclusion is finished. Subsequently, samples from the Erasmus MC will be transported to the UMCU. At the UMCU, all samples will be analyzed at once. In case it is unlikely at admission that the patient will be able to undergo CSF sampling, the patient is not included in this study (see section 4.3 *"Exclusion criteria"*). Obtaining a CSF sample from an external ventricular or lumbar drain can potentially increase the risk of infection (secondary meningitis or ventriculitis).[29] If a patient does not have an external ventricular or lumbar drain, a lumbar puncture will be done for the purpose of this trial. For sedated and mechanically ventilated patients the depth of the sedation can be temporarily increased to reduce the burden of a lumbar puncture for the patient. If a lumbar puncture is performed, there is a risk of: 1) post-lumbar puncture headache (40% risk); 2) back pain (30% risk); and 3) minor neurologic symptoms such as radicular pain or numbness (13% risk).[30–32] Very rare complications of a lumbar puncture include infection (local infection, meningitis, discitis and, spinal epidural abscess), bleeding (spinal epidural or subdural hematoma and, intracranial bleeding), and abducens palsy.[33–37]

<u>Neurological examinations</u>

*Neurological evaluations that are part of standard SAH treatment:*

Daily neurological examination (Glasgow coma scale, focal neurological deficits) by the treating physician is part of standard SAH care. Before discharge, a complete neurological examination is performed by the treating physician as part of standard SAH care.

*Neurological evaluations that are extra for this study:*

On day 14 the WFNS and NIHSS score will be completed (see appendix 3 and 4 *"NIHSS score and WFNS score"*). Since a complete neurological examination is performed before discharge as part of standard SAH care, we do not expect that the WFNS score will be an extra burden for the patient. The NIHSS score consists of a complete neurological examination and two additional questions (month and age; blink and squeeze hands). The two additional questions will only pose a very minor burden for the patient.

<u>Brain MRI</u>

*Imaging procedures that are part of standard SAH treatment:*

A brain MRI at discharge is not part of standard SAH treatment.

*Imaging procedures that are extra for this study:*
A brain MRI scan will be performed at hospital discharge, when the patient is in a stable condition to evaluate the extent of ischemic brain injury. The patient needs to be able to safely undergo MRI scanning (e.g. the patient does not have any contraindications for MRI scanning). Before the MRI scan is performed, the investigator or nurse will discuss the MRI safety questions with the patient (an example of the MRI safety questionnaire can be found in E1/E2 “patient information letter”). If the patient has contra-indications for brain MRI scanning, the brain MRI scan will not be performed (this will be registered as “missing data”). In case the patient is not in a stable condition, the MRI will be postponed until the patient is in a stable condition (with a maximum delay of three weeks after clinical deterioration suggestive for cerebral ischemia). Contrast agents (such as gadolinium) will not be used. There is no extra risk related to MRI scanning in this patient population. A 3T MRI scanner (Philips Medical Systems, Best, the Netherlands) will be used in this study. The MRI protocol consist of a 3D-T1-weighted sequence, a T2-weighted sequence, a Fluid-Attenuated Inversion Recovery (FLAIR) sequence, a Diffusion Weighted Imaging (DWI) sequence, and an Apparent Diffusion Coefficient (ADC). The presence of cerebral infarction (measured by a high DWI signal) and the volume of the cerebral infarction will be analysed. The volume of the cerebral infarction will be measured through manual segmentation on either the FLAIR or DWI images by using picture Archiving and Communication System (PACS) software (Sectra AB, Linköping, Sweden). Patients with a coincidental finding on the MRI, which was not visible on head CT or CT angiography will receive the appropriate clinical follow-up. The subsequent follow-up will not be covered by the insurance for patients participating in this study but has to be covered by the patient’s own insurance.

<u>Cognitive testing and QoL.</u>
*Procedures that are part of standard SAH treatment:*
Patients visit the SAH outpatient clinic six to eight weeks after discharge. At the UMCU outpatient clinic, the patient is examined by a nurse specialist, a neuropsychologist and a rehabilitation physician. At the Erasmus MC outpatient clinic, the patient is examined by either the neurologist and neurointerventionalist or the neurosurgeon.

*Procedures that are extra for this study:*
Four weeks after ictus (+/- one week) a questionnaire on possible AEs or SAEs will be sent to the patient or treating physician unless the required follow-up of AEs and SAEs was already performed in the hospital.Ten weeks after ictus (+/- two weeks) during the outpatient clinic, patients will be asked to perform a cognitive test and fill out a questionnaire on QoL: 1) the MoCA; and 2) the EQ-5D-5L (appendix 5 and 6 *“MoCA score” and “EQ-5D-5”)*. The

MoCA is a cognitive screening test used as a screening tool for cognitive impairment. It is more sensitive than the MMSE to pick up mild cognitive impairment in patients who suffered from a subarachnoid hemorrhage.[38–40] The EQ-5D-5L is a standardized instrument to asses health care status and consists of five questions on five different levels.[41–43]

mRS score

*Procedures that are extra for this study:*

During telephone consultation 13 weeks (+/- two weeks) after ictus, the mRS score (appendix 8 "*mRS score*") will be determined.

### 8.4 Withdrawal of individual subjects

Subjects can leave the study at any time for any reason without any consequences, if they wish to do so. If a patient or legally acceptable surrogate decides to leave the study, the date and the primary reason for withdrawal will be recorded in the eCRF. If it appears after randomization that the patient did not meet the enrolment criteria, treatment will be discontinued and the patient will be withdrawn from the study. The investigator can decide to withdraw a subject from the study for urgent medical reasons including SAEs. In the latter case, the acquired data will be used for analysis (since informed consent was already obtained prior to inclusion).

#### 8.4.1 Specific criteria for withdrawal

Treatment with eculizumab will be discontinued in case of the following SAEs:

1) Anaphylactic shock after infusion of eculizumab. In case of a mild allergic reaction the infusion rate can be slowed down with a maximum infusion duration of up to two hours.
2) Patients with Neisseria meningitis, CSF culture proven.
3) Patients with meningococcal sepsis, blood culture proven.
4) Other medical reasons for which the treating physician or investigator deem it necessary to discontinue treatment.

Antibiotic treatment with ciprofloxacin will be discontinued in case of:

1) Anaphylactic shock
2) Epileptic seizures
3) Depression, psychosis, suicidal ideations/thoughts
4) Severe and persistent diarrhoea during treatment
5) Signs and symptoms of hepatic disease

6) Tendinitis and tendon rupture
7) Symptoms of neuropathy

If possible, amoxicillin will be given instead of ciprofloxacin until four weeks after ictus.

Treatment with fluconazole will be discontinued in case of:

1) Anaphylactic shock
2) Hepatitis
3) Other side-effects for which the physician/trial coordinator deems it necessary to stop prophylactic treatment

In case prophylactic treatment with fluconazole needs to be stopped, we will consult the microbiologist to discuss other prophylactic treatment options.

### 8.5 Replacement of individual subjects after withdrawal

Patients who leave the study before CSF sampling can be obtained will be replaced by another subject according to a “per protocol” analysis to allow analysis for the primary objective.

### 8.6 Follow-up of subjects withdrawn from treatment

Patients withdrawn from this study will not undergo any further treatment, sampling or imaging for this study. After withdrawal, SAEs and AEs will be monitored for four weeks after ictus. Depending on the length of stay, this will occur during in-hospital stay and/or after discharge with a questionnaire on AEs and SAEs. In case the patient or the treating physician did not return a completed questionnaire, the investigator or research nurse will phone the patient or treating physician and kindly remind him/her to return the questionnaire. After two reminders by telephone, a visit to the patient’s home, rehabilitation center or nursing home will be planned in agreement with the patient or treating physician to follow-up AEs and SAEs.

### 8.7 Premature termination of the study

In patients with aneurysmal SAH, the 90-day case-fatality is approximately 30% according to a study conducted at the UMCU.[6] In this population, disease-related SAEs and AEs occur frequently, such as rebleeding of the aneurysm, delayed cerebral ischemia, and hydrocephalus. An external DSMB will be asked to evaluate safety of the study during the

trial. An interim analysis will be performed when the first 20 patients are included. The external DSMB consists of Prof. Dr. J. Stam (chair), Prof. Dr. D. van de Beek, and Dr. H.F. Lingsma. If the DSMB concludes that treatment with eculizumab is unsafe, the study will be terminated prematurely. Criteria for early termination of this study are described in section 10.3 *"Interim analysis"*. After the DSMB recommends terminating the study, all the involved investigators have to be informed immediately. In case the main investigators and/or trial steering committee agree with the recommendation from the DSMB, the appropriate authorities (including the ethics committee and Alexion) will be informed promptly. The decision will be binding to both participating hospitals (UMCU and Erasmus MC) and their investigators. After closure of the trial, all study materials which can be returned to the sponsor under the appropriate regulations must be returned to the sponsor. See section 9.5 "*Data Safety Monitoring Board*" for the procedures when the recommendation of the DSMB is not fully implemented.

# 9. SAFETY REPORTING

## 9.1 Temporary halt for reasons of subject safety

In accordance to section 10, subsection 4, of the WMO, the sponsor will suspend the study if there is sufficient ground that continuation of the study will jeopardise subject health or safety. The sponsor will notify the accredited METC without undue delay of a temporary halt including the reason for such an action. The study will be suspended pending a further positive decision by the accredited METC. The investigator will take care that all subjects are kept informed.

## 9.2 AEs, SAEs and SUSARs

All AEs, SAEs and SUSARs will be reported between study inclusion and up until four weeks after ictus. The definitions of AEs, SAEs and SUSARs as described below will be used for the present study. The forms on which the AEs and SAEs will be filled out can be found in appendix 9, and 10 *"AE report form and SAE report form"*.

### 9.2.1 Adverse events (AEs)

AEs are defined as any undesirable experience occurring to a subject during the study, whether or not considered related to the investigational product or samples obtained during the study. All AEs reported spontaneously by the patient or observed by the investigator or his staff will be recorded up until four weeks after ictus. Adverse events will be graded according to the Common Terminology Criteria for Adverse Events v4.0 (CTCAE, v4.03: June 14, 2010):

| | |
|---|---|
| Grade 1: | Mild: signs and symptoms that can be easily tolerated. Symptoms can be ignored or disappear when the subject is distracted. |
| Grade 2: | Moderate: symptoms cause discomfort but are tolerable, they cannot be ignored and affect normal activity. |
| Grade 3: | Severe: symptoms strongly affect normal activity. |

### 9.2.2 Serious adverse events (SAEs)

A serious adverse event is any untoward medical occurrence or effect that

- results in death;
- is life threatening (at the time of the event);
- requires hospitalisation or prolongation of existing inpatients' hospitalisation;

- results in persistent or significant disability or incapacity;
- is a congenital anomaly or birth defect; or
- any other important medical event that did not result in any of the outcomes listed above due to medical or surgical intervention but could have been based upon appropriate judgement by the investigator.

An elective hospital admission will not be considered as an SAE.

### 9.2.3 Recording and reporting of AEs and SAEs

All SAEs and AEs (related or unrelated to aneurysmal SAH) reported by the subject, investigator or staff will be recorded for both groups (eculizumab and control group) from study inclusion until four weeks after ictus. The following information for SAEs and AEs will be collected in the electronic case report form (eCRF):

- Subject number
- Demographic data
- Brief description of the event
- Onset date and time
- Date and time of recovery (if the event resolved)
- Current status (if event has not resolved)
- Action taken (intervention)
- Outcome of the AE
- The relationship between the event and study drug

The potential relationship between the event and the study drug will be assessed by the investigator or principal investigator at the UMCU or Erasmus MC and recorded as either ‘definite’, ‘probable’, ‘possible’, ’unlikely’, ‘not related’ or ‘not assessable’ for AEs and as ‘definite’, ‘probable’, ‘possible’, ‘unlikely’ or ‘unrelated’ for SAEs.
All SAEs will be reported through the web portal *ToetsingOnline* to the accredited METC that approved the protocol, within 7 days of first knowledge for SAEs that result in death or are life threatening followed by a period of maximum of 8 days to complete the initial preliminary report. All other SAEs will be reported within a period of maximum 15 days after the sponsor has first knowledge of the serious adverse events.

SAEs potentially related to the study drug will also be reported to Alexion’s Pharmacovigilance Department within one business day of the investigator becoming aware

of such SAE.

### 9.2.4 Suspected unexpected serious adverse reactions (SUSARs)

Adverse reactions are all untoward and unintended responses to an investigational product related to any dose administered.

Unexpected adverse reactions are SUSARs if the following three conditions are met:

1. the event must be serious (see chapter 9.2.2);
2. there must be a certain degree of probability that the event is a harmful and an undesirable reaction to the medicinal product under investigation, regardless of the administered dose;
3. the adverse reaction must be unexpected, that is to say, the nature and severity of the adverse reaction are not in agreement with the product information as recorded in:
   - Summary of Product Characteristics (SPC) for an authorised medicinal product;
   - Investigator’s Brochure for an unauthorised medicinal product.

In case, the relationship between the SAE and study drug is classified either as ‘definitely’, ‘probable, ‘possible’ or ‘not assessable’ and the SAE is unexpected it will be reported as a SUSAR.

The sponsor will report expedited all  SUSARs through the web portal *ToetsingOnline* to the METC.

The expedited reporting of SUSARs through the web portal Eudravigilance or ToetsingOnline is sufficient as notification to the competent authority.

The expedited reporting will occur not later than 15 days after the sponsor has first knowledge of the adverse reactions. For fatal or life threatening cases the term will be maximal 7 days for a preliminary report with another 8 days for completion of the report.

The aforementioned definitions and regulations on AEs, SAEs and SUSARs also apply to the Erasmus MC. The trial coordinator at the Erasmus MC will be Dr. M. van der Jagt, who will inform the trial coordinator or principal investigator at the UMCU within one business day of

SAEs and SUSARs in order to report the SAEs and SUSARs in time to the appropriate authorities.

### 9.3 Annual safety report

In addition to the expedited reporting of SUSARs, the sponsor will submit, once a year throughout the clinical trial, a safety report to the accredited METC and competent authority.

This safety report consists of:

- a list of all suspected (unexpected or expected) serious adverse reactions, along with an aggregated summary table of all reported serious adverse reactions, ordered by organ system, per study;
- a report concerning the safety of the subjects, consisting of a complete safety analysis and an evaluation of the balance between the efficacy and the harmfulness of the medicine under investigation.

### 9.4 Follow-up of adverse events

A questionnaire on AEs and SAEs (appendix 7 *"Questionnaire on AEs and SAEs"*) will be sent to the home address of the patient four weeks after ictus (+/- 1 week) unless the required follow-up of AEs and SAEs was already performed in the hospital. In case the patient is not able to fill out the questionnaire, it will be sent to the treating physician either from the rehabilitation center or the nursing home. The patient or the treating physician will be asked to fill out whether he/she or the patient experienced AEs or SAEs within four weeks after ictus. If the questionnaire was not returned by the patient or treating investigator, the procedures as specified in section 8.6 will be followed. The AEs and SAEs follow-up of four weeks was chosen since the half-life of eculizumab is 11 days and the last infusion with eculizumab is on day 7.[44] In case of a CSF culture proven drain associated infection in patients who are treated with eculizumab, the SAE follow-up will be extended to 3 months after ictus.

All AEs will be followed until they have abated, or until a stable situation has been reached. Depending on the event, follow up may require additional tests or medical procedures as indicated, and/or referral to the general physician or a medical specialist.
SAEs need to be reported until end of study within the Netherlands, as defined in the protocol

### 9.5 Data Safety Monitoring Board (DSMB)

The DSMB is an independent committee that will monitor the safety of eculizumab. The committee consists of Prof. Dr. J. Stam (chair), Prof. Dr. D. van de Beek, and Dr. H.F. Lingsma. The DSMB will use the template charter developed by the DAMOCLES study group to structure and organize work-flow.[45] Safety will be examined by ongoing monitoring of SAEs and SUSARs and by an interim analysis. The following SAEs will be reported within one business day to the DSMB chairman: 1) anaphylactic shock; 2) meningococcal sepsis, blood culture proven; 3) Neisseria meningitis, CSF culture proven; 4) CSF culture proven drain associated infection; 5) malignant melanoma, myelodysplastic syndrome; and 6) death. Listings of infections will be reported every two months to the DSMB chairman. Listings of all SAEs and SUSARs will be provided every half year to the DSMB chairman (in addition to the line list provided to the METC). Interim analysis will be performed when 20 patients are enrolled (see section 10.3 *'Interim analysis'*). The advice(s) of the DSMB will only be sent to the sponsor of the study. Should the sponsor decide not to fully implement the advice of the DSMB, the sponsor will send the advice to the reviewing METC, including a note to substantiate why (part of) the advice of the DSMB will not be followed.

# 10. STATISTICAL ANALYSIS

The mean and standard deviation will be reported for continuous data with a parametric distribution and the median with its interquartile range for continuous data with a non-parametric distribution. Categorical data will be presented as proportions or rates. The primary analysis will be a “per protocol analysis” performed for all outcome measures. A “per protocol analysis” was selected since the primary outcome measurement is biological effectivity determined by the C5a concentration in CSF. In addition to the “per protocol analysis”, an “intention-to-treat analysis” will also be performed to compare results of both analyses. Statistical significance is defined by a two-sided p-value < 0.05.

## 10.1 Primary study parameters

C5a concentration in CSF 48-72 hours after ictus

Assuming a parametric distribution, the mean differences and the corresponding 95% confidence intervals will be calculated for both the intervention and control group and an independent t-test will be performed. In case of a non-parametric distribution, the Mann-Whitney U-test will be applied. Multivariable linear regression analysis will be applied to adjust the GCS score.

Multiple testing

In this study there is one primary outcome measure, so there will be no multiple testing problem.

## 10.2 Secondary study parameters

Continuous variables

Mean differences with corresponding 95% confidence intervals will be computed for continuous variables with a parametric distribution and an independent t-test will be applied. In case of non-parametric distribution the Mann-Whitney U-test will be applied. Multivariable linear regression analysis will be performed to adjust for GCS. Repeated measurements will be analysed with a linear mixed model.

Categorical variables

Possible-, probable- or definitely-related AEs and SAEs will be categorized according to the terms of the MedDRA classification and presented as tabulated incidence rates. A proportional odds model will be used to assess the effect of eculizumab on WFNS and mRS score.[46] Categorical data will be analysed with the Chi-square or Fisher’s exact test. Multivariable logistic regression analysis will be applied to adjust for GCS.

### 10.3 Interim analysis

A DSMB is established to assess safety of the study. The DSMB will perform an interim analysis on when half of the patients are included (n=20). If there is evidence of severe harm based on SAE reporting, outcome, or case fatality, the DSMB can recommend the steering committee to terminate the study.

# 11. ETHICAL CONSIDERATIONS

## 11.1 Regulation statement

The study will be conducted according to the principles of the Declaration of Helsinki *(64th WMA General Assembly, Brazil, October 2013, www.wma.net)* and in accordance with the Medical Research Involving Human Subjects Act (WMO) and the Good Clinical Practice *(ICH harmonised guideline on Good Clinical Practice, November 2016)*.

## 11.2 Recruitment and consent

An aneurysmal SAH is a life-threatening medical emergency. To potentially reduce early brain injury (<72 hours after ictus) eculizumab is administered as soon as possible but not later than 12 hours after ictus. Acute treatment is essential to investigate the effect of eculizumab on early brain injury. Patients included in our study will, in most cases, not be capable to give informed consent. By postponing treatment, there may be no effect of eculizumab on early brain injury. A patient or legally acceptable surrogate (depending on whether the patient is capable of giving informed consent) will be asked for full informed consent. Because treatment with the study drug needs to be initiated within 12 hours after ictus, the patient or legally acceptable surrogate will have limited time to consider (the patient's) participation in the CLASH study. Time to consider participation will vary depending on when the patient is admitted to the hospital. Informed consent will be asked at either the emergency department, ICU, neurological/neurosurgical HCU, or neurological/neurosurgical MCU (if possible in a separate room) by the investigator or treating physician. The patient or legally acceptable surrogate will be provided with information on the rationale of the study, the procedures, the duration of the study, its potential benefits and risks, and any discomfort the study entails. The information letter and informed consent papers will be given to the patient or legally acceptable surrogate. The investigator or treating physician will emphasize that the choice for participation or non-participation will not affect the quality of care and that they can decide to refuse participation or withdraw from the study at any time, also when the medication is already administered. If written informed consent is obtained, randomization will occur. Depending on allocation, treatment with eculizumab in addition to standard SAH care or only standard SAH care is given. The clinical condition of the patient will be checked daily by the investigator or treating physician to determine whether the patient is capable of giving informed consent (if the patient was incapacitated on admission). In case the patient is capable of giving informed consent, the patient will be asked for written informed consent. In addition, informed consent will be obtained for the use of the patient's data. In case the patient is not able to write, oral consent can be given in the presence of a witness. The witness needs to sign the informed consent form and write down the date of informed

consent obtainment. The informed consent procedure will be described in the electronic patient system. In case the patient is not able to read, an independent witness needs to be present during the informed consent procedure. After the informed consent procedure is read aloud and oral consent is obtained, the patient needs to sign the informed consent form and write down the date of informed consent obtainment. In addition, the independent witness needs to sign the informed consent and write down the date of informed consent obtainment. By signing, the independent witness declares that the information is explained accurately and that the patient understands the information and voluntary agrees to participate. The informed consent procedure will also be described in the electronic patient system. The opinion of the patient is leading in the eventual decision on participation in this study. If the legally acceptable surrogate has agreed to the patient's participation but the patient, at a later stage, does not want to participate in the study, the patient will be excluded from the study and treatment will be discontinued. The patient will be asked for informed consent to use the acquired data. In case the patient dies before informed consent could be obtained but informed consent has been given by the legally acceptable surrogate, the acquired data will be used for analysis. A detailed overview of the recruitment procedure for patients who are incapacitated on admission is given in *Figure 7*.

**Figure 7. Recruitment and consent procedure**

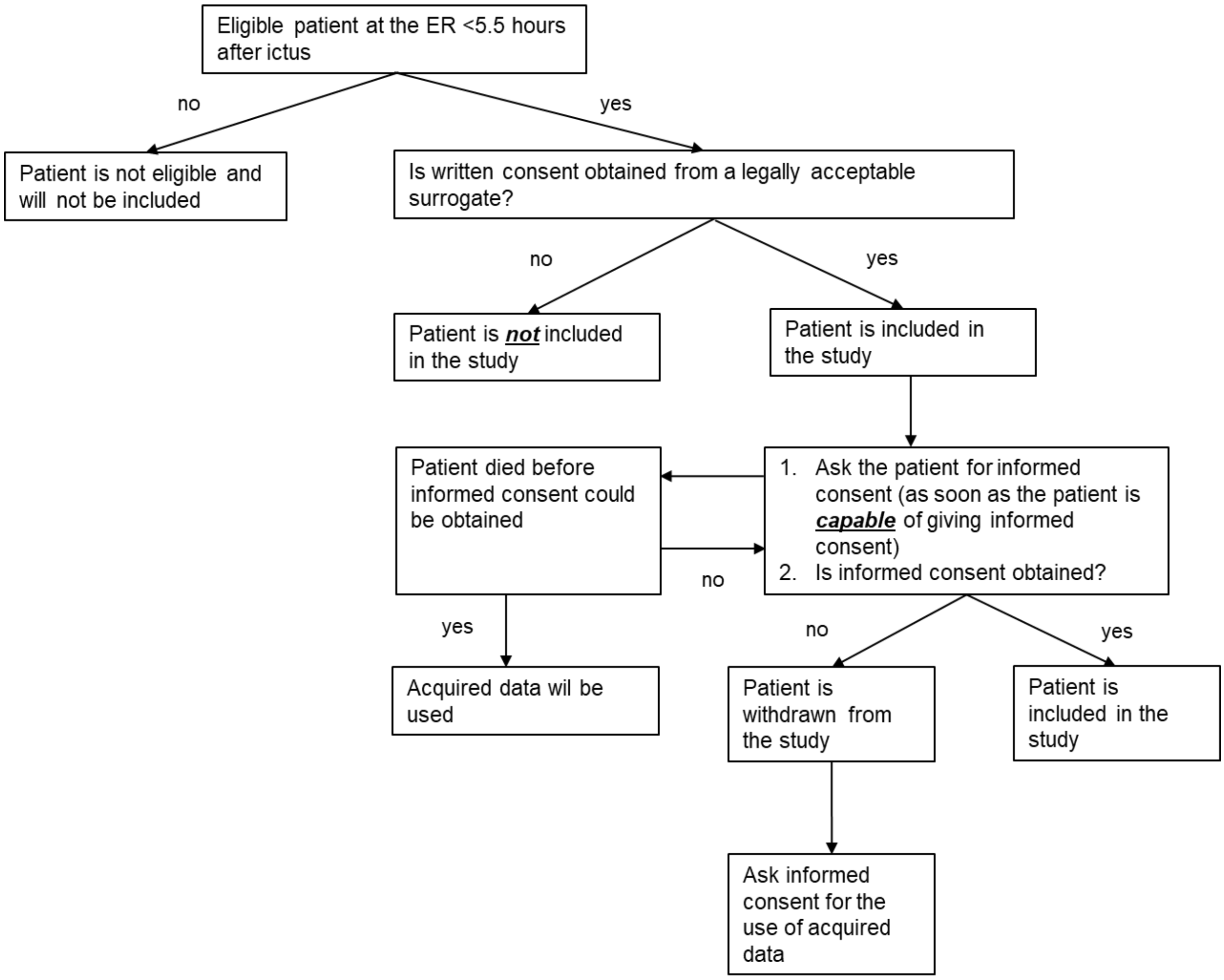


Copies of the informed consent form will be given to the patient and/or legally acceptable surrogate and the original will be maintained with the patient's records. The general practitioner will be informed of the patient's participation in this study after inclusion. In addition, the patient discharge letter will also mention the patient's participation in the CLASH study and the phone number of the investigator in case questions arise. If new safety information results in significant changes in the risk/benefit assessment, the patient information and informed consent form will be reviewed again and updated if necessary. All patients already included in the study will be informed of the new information and asked if they want to continue with the study.

### 11.3 Objection by minors or incapacitated subjects

Minors will not be included in this study. Incapacitated adults will be included in this study (WFNS score 1-5). On admission, a legally acceptable surrogate will be asked for informed consent (in case the patient is incapacitated). As described in section 11.2 "*Recruitment and consent*", daily evaluation of the clinical condition will take place by the treating physician or

investigator and informed consent from the patient will be obtained when the patient is considered capable of giving informed consent (if the patient was incapacitated on admission). The latter decision will be made by either the treating physician or the investigator.

### 11.4 Benefits and risks assessment, group relatedness

Potential adverse effects of treatment with eculizumab include among others headache, nausea and increased risk of infections. Patients can experience similar adverse events (headache, nausea, infections) after aneurysmal SAH. Standard SAH care includes symptomatic treatment for headache and nausea (and if necessary antibiotics to treat an infection). Serious adverse effects include severe infection and sepsis, in specific increased susceptibility to infection with the meningococcus bacteria (*Neisseria meningitidis*) and an anaphylactic reaction. To reduce the risk of infection with the meningococcus bacteria, patients in the intervention group will receive prophylactic treatment with ciprofloxacin during the first four weeks after ictus. Patients in the intervention group with a central line or an external lumbar or ventricular drain and a positive yeast culture will also receive antifungal prophylaxis to decrease the risk of infection with a fungus. Multi-drug resistance and yeast carriership/colonization will be closely monitored by throat and rectal swaps. If the multi-drug resistance culture is positive, the microbiologist will be consulted and prophylactic treatment with ciprofloxacin will be switched to a different prophylactic antibiotic that sufficiently covers the increased risk of infection. In addition, safety instructions and a patient safety card will be provided to the patients in the intervention group before discharge. These patients will be asked to carry the patient safety card until four weeks after ictus on their person. Anaphylactic reactions have not been observed in the published literature on trials with eculizumab treatment in patients with PNH or aHUS.[17,21–24] The first infusion with eculizumab will be either at the emergency department, ICU, neurological/neurosurgical HCU, or neurological/neurosurgical MCU where continuous monitoring is present. If an anaphylactic shock would occur, this will be noted immediately and direct actions can be taken. Current practice at the UMCU in patients with PNH and aHUS, who receive their first eculizumab infusion is that the patients are observed for an hour after which they go home. This observation time is not considered necessary during subsequent infusions in patients with PNH and aHUS. Those patients can go home after the infusion with eculizumab if they do not experience any side-effects. Therefore, we do not foresee any risks of giving subsequent eculizumab infusions at the neurological/neurosurgical ward.

A potential risk of long-term antibiotic use is antibiotic resistance. However, multi-drug resistance will be closely monitored and prophylactic antibiotics will be changed if necessary.In addition, we exclude patients with a high risk of antibiotic resistance (patients who have received antibiotic treatment >4 times during the last year will be excluded). Potential side-effects of ciprofloxacin ( 1 out of 10 patients) are: nausea, diarrhea. Very rare (<1/10,000) but more serious side-effects are haemolytic anemia, agranulocytosis, pancytopenia, bone marrow depression, anaphylaxis, mental/mood changes, migraine or coordination disorders, visual color distortions, vasculitis, pancreatitis, liver necrosis, petechiae and muscle weakness. Potential side effects of fluconazole are (1 out of 10 patients): headache, abdominal pain, diarrhea, nausea or vomiting, rash, and increased alkaline phosphatase, aspartate transaminase, and/or alanine transaminase. Rare (≥1/10,000 to <1/1,000) but more serious side-effects are among others: agranulocytosis, leukopenia, thrombocytopenia, neutropenia, anaphylaxis, torsades de pointes, hepatic failure, hepatocellular necrosis and hepatitis. A complete list of all potential side-effects can be found in the E1/E2 '*patient information letter'*. Patients with a known allergy to quinolones (including ciprofloxacin), a creatinine clearance of <30 or serum creatinine levels of >169 µmol/l, patients who use tizanidine and patients with myasthenia gravis, glucose-6-phosphate dehydrogenase (G6PD) deficiency, or tuberculosis are excluded from participation in this study (4.3 '*Exclusion criteria*'). Treatment with ciprofloxacin and/or fluconazole will be discontinued and switched  if necessary (8.4.1 '*Specific criteria for withdrawal').*The medical staff is instructed to be alert on possible medication interactions of ciprofloxacin and fluconazole and to be cautious with the use of ciprofloxacin in patients with 1) congenital long QT syndrome; 2) concomitant use of drugs that are known to prolong the QT interval (e.g. Class IA and III anti-arrhythmics, tricyclic antidepressants, macrolides, antipsychotics); 3) uncorrected electrolyte imbalance (e.g. hypokalaemia, hypomagnesaemia); 4) cardiac disease (e.g. heart failure, myocardial infarction, bradycardia); and 5) patients with diabetes mellitus. Ciprofloxacin and fluconazole can be administered concomitantly according to the microbiologist and internist-infectiologist. The risk associated with CSF sampling from an external ventricular or external lumbar drain includes a potentially increased risk of infection.[29] The risks associated with a lumbar puncture include among others post-lumbar puncture headache. [30–32] Very rare complications include infection, bleeding and abducens palsy. [33–37] A lumbar puncture is a procedure that is part of standard diagnostic or therapeutic work-up for some neurological disorders and is therefore often performed at the neurology/neurosurgery department. Minor risks are associated with blood withdrawal and include bruising, redness and swelling, and a very small chance of infection and fainting.

There are no additional risks associated with neurological examinations (standard SAH care) and MRI scanning for which no contrast agent will be used.
The MoCA, ED-5Q-5L, questionnaire on AEs and SAEs , and mRS score will not pose additional risks during the outpatient visit or telephone consultation.

Aneurysmal SAH patients receiving standard clinical care have a 30% case-fatality rate and another 30% of the patients remains dependent.[47] Important determinants of case-fatality and disability are early brain injury and delayed cerebral ischemia .[48] No treatment exists to reduce early brain injury and the effect of current treatment strategies to prevent delayed cerebral ischemia are only modest. Eculizumab could potentially decrease early brain injury and delayed cerebral ischemia and thereby improve the prognosis of the patient.

After careful examination of the risks and benefits, we expect that patients can benefit from treatment with eculizumab because its potential positive effect on brain injury. It is expected that the overall effect on neurological outcome outweighs the potential side-effects.

### 11.5 Compensation for injury

The sponsor/investigator has a liability insurance which is in accordance with article 7 of the WMO.

The sponsor has an insurance which is in accordance with the legal requirements in the Netherlands (Article 7 WMO). This insurance provides cover for damage to research subjects through injury or death caused by the study.
The insurance applies to the damage that becomes apparent during the study or within 4 years after the end of the study.

# 12. ADMINISTRATIVE ASPECTS, MONITORING AND PUBLICATION

## 12.1 Handling and storage of data and documents

All individual patient data obtained during this study will be handled according to the Dutch Personal Protection Act *(De Wet Bescherming Persoonsgegevens, WBP).*

The Julius Center will construct a specific and secured database using Research Online. Access will only be provided to authorized staff. Each patient will receive a personal identification number. Subject data will be registered in Research Online using the personal identification number. Data will therefore be stored pseudonymously in Research Online. Only the investigators and the treating physicians will be able to decode the pseudonymous data. After inclusion is completed and all follow-up assessments are done, the database will be locked. The trial coordinator at the Erasmus MC will also be able to login to Research Online to add enrolled subjects. The following information will among others be in the Investigator Site File (ISF): 1) Informed consent forms; 2) reports of AEs and SAEs 3) MoCA; 4) ED-Q5-5L; 5) questionnaires on AEs and SAEs; and 6) clinical trial agreement between Erasmus MC and UMCU. Upon completion of the study, all paper study files, which can be returned to the sponsor under the appropriate regulations, will be archived at the neurology department of the UMCU. Data (paper and electronic) will be kept in storage for 15 years.

Individual subject data stored in Research Online includes: 1) subject and personal identification number; 2) date and time of ictus; 3) date and time of admission; 4) site of inclusion; 5) date and time of inclusion; 6) date and time of study drug administration (three times eculizumab); 7) date and time of CSF collection; 8) age at inclusion; 9) sex; 10) duration of loss of consciousness during ictus; 11) clinical condition on admission (WFNS score); 12) daily Glasgow Coma Score (GCS) during the first 14 days after ictus; 13) WFNS- and National Institutes of Health Stroke Scale (NIHSS) score 14 days after ictus; 14) smoking status; 15) history of hypertension; 16) aneurysm size and location; 17) blood volume on admission head computed tomography (CT) according to the Hijdra score; 18) aneurysm treatment modality (endovascular or microneurosurgical clipping); 19) date and time of aneurysm treatment; 20) occurrence of rebleeding; 21) date and time of rebleeding; 22) occurrence of delayed cerebral ischemia; 23) date and time of delayed cerebral ischemia; 24) bacterial infections of various causes (e.g. pneumonia, urinary tract infection and meningitis/ventriculitis); 25) date and time of infection; 26) concentration of C5a in CSF; 27) blood and CSF parameters of inflammation (e.g. CRP, cytokines, C5b-9); 28) concentration of eculizumab in blood and CSF; 29) GCS for the first 14 days; 30) WFNS and NIHSS score at day 14; 31) date of MRI scan; 32) the presence and volume of cerebral infarction on brain magnetic resonance imaging (MRI); 33) occurrence of AE and SAE up to

four weeks after ictus; 34) MoCA score; 35) ED-5Q-5L score; and 36) mRs score. Time will be denoted in hours and minutes.

### 12.2 Monitoring and Quality Assurance

A risk assessment was made using the guidelines from the Dutch Federation of Medical Hospitals (NFU; Nederlandse Federatie van Universitaire Medische Centra). The risk was estimated at 'moderate' since eculizumab is currently already used as treatment for patients with PNH and aHUS and accepted by FDA and EMA onto the American and European market. In the CLASH trial we will use the same dose as the dose approved for patients with aHUS. Various safety measurements will be applied in this study which include prophylactic antibiotics, close monitoring, safety instructions and a patient safety card. Also, in previous Alexion-sponsored clinical studies in which patients received long-term treatment, the rate of meningococcal infection was still very low: 2/195 (1%) in patients with PNH and 3/130 (2.3%) in patients with aHUS (data from Investigator's brochure page 48). In accordance with the NFU guidelines, external monitoring will be provided by Julius Clinical. Monitoring activities will be based on a 'moderate risk' classification and includes moderate-intensive monitoring according to the NFU guidelines. Details can be found in the monitoring plan K6 "*Monitoring plan*".

### 12.3 Amendments

A 'substantial amendment' is defined as an amendment to the terms of the METC application, or to the protocol or any other supporting documentation, that is likely to affect to a significant degree:

- the safety or physical or mental integrity of the subjects of the trial;
- the scientific value of the trial;
- the conduct or management of the trial; or
- the quality or safety of any intervention used in the trial.

All substantial amendments will be notified to the METC and to the competent authority.

Non-substantial amendments will not be notified to the accredited METC and the competent authority, but will be recorded and filed by the sponsor.

### 12.4 Annual progress report

The sponsor/investigator will submit a summary of the progress of the trial to the accredited METC once a year. Information will be provided on the date of inclusion of the first subject,

numbers of subjects included and numbers of subjects that have completed the trial, serious adverse events/ serious adverse reactions, other problems, and amendments.

### 12.5 Temporary halt and (prematurely) end of study report

The sponsor will notify the accredited METC and the competent authority of the end of the study within a period of 90 days. The end of the study is defined as the last patient's last visit. The sponsor will notify the METC immediately of a temporary halt of the study, including the reason of such an action.

In case the study is ended prematurely, the sponsor will notify the accredited METC and the competent authority within 15 days, including the reason(s) for the premature termination.

Within one year after the end of the study, the investigator/sponsor will submit a final study report with the results of the study, including any publications/abstracts of the study, to the accredited METC and the Competent Authority.

### 12.6 Public disclosure and publication policy

The CCMO (Centrale Commissie Mensgebonden Onderzoek) statement containing the basic principles on the public disclosure and publication policy will be followed (www.CCMO.nl). The results of this study will be published within 12 months after the end of the study. The study will be registered prospectively in the EudraCT database, where also the results will be registered. Furthermore, the study will be registered at the Netherlands Trial Register (NTR).

# 13. STRUCTURED RISK ANALYSIS

## 13.1 Potential issues of concern

a. Level of knowledge about mechanism of action

Eculizumab is a complement inhibitor that specifically and with high affinity binds to the complement protein C5, thereby inhibiting its cleavage to C5a and C5b.[22] C5a is a potent anaphylactic chemotactic mediator which activates a pro-inflammatory response. C5b also has a proinflammatory effect and assembles the terminal complement complex C5b-9 which can induce cell lysis.[49]

b. Previous exposure of human beings with the test product(s) and/or products with a similar biological mechanism

In 2007, eculizumab was approved by the FDA and EMA for treatment of patients with PNH. Four years later, eculizumab was approved by the aforementioned authorities for treatment of patients with aHUS (pediatric and adult patients). Recently, refractory generalized myasthenia gravis in patients who are anti-acetylcholine receptor (AChR) antibody-positive has been approved as a new indication by the EMA.
Multiple clinical trials have been conducted with eculizumab. The most commonly reported side-effect is headache. The most common serious side effect among meningococcal infections was meningococcal sepsis (see the *'Summary of product characteristics'* for a list of side-effects).

c. Can the primary or secondary mechanism be induced in animals and/or in *ex-vivo* human cell material?

Previous studies in animals and on human cell material found the following results:

1) In patients with SAH, C5a concentration in CSF was markedly increased (>800-fold) 1 day after SAH compared with controls, and decreased over the first 14 days after ictus.
2) Brains of patients who died from aneurysmal SAH had much higher complement expression than brains from controls. This difference was most pronounced in areas with brain infarction.
3) In a SAH mouse model, brain injury was >40% reduced in C5a receptor knock-out mice and in wildtype mice treated with C5 antibodies compared with untreated wildtype mice.[13]

d. Selectivity of the mechanism to target tissue in animals and/or human beings

The following information is based on two report from the EMA on eculizumab.[19,26]

Eculizumab is a humanized specie-specific monoclonal antibody that binds to the C5 complement protein and inhibits cleavage of C5 into C5a and C5b. Eculizumab demonstrated no cross-reactivity against any other mammalian species (also not for chimpanzee, baboon and rhesus). Since eculizumab is a humanized specie-specific antibody, animal studies with eculizumab are not possible. Animal studies therefore use a surrogate for eculizumab: the anti-mouse C5 antibody.

<u>e. Analysis of potential effect</u>

In a SAH mouse model, brain injury was >40% reduced in wildtype mice treated with C5 antibodies compared with untreated wildtype mice.[13] A similar reduction in brain injury was found in C5aR knockout mice compared with wildtype mice.

<u>f. Pharmacokinetic considerations</u>

The half-life of eculizumab after intravenous infusion was 272 hours (standard deviation of 82 hours) with drug distribution primarily confined to the vascular space.[22,44] Previous studies show that the C5a concentration in CSF is >800 times elevated at day 1 after SAH, with a gradual decrease in the first two weeks after aneurysmal SAH.[13] After two weeks, C5a levels were still not normalized. Therefore, a dose of 1200 mg and repeat drug administration on day 3 and day 7 was chosen to prevent wash-out of eculizumab. The dose of 1200 mg eculizumab is approved as maintenance therapy in patients with aHUS.

<u>g. Study population</u>

This study includes patients with aneurysmal SAH who are admitted within 11.5 hours after ictus. Patients with a WFNS score of 1-5 on admission are eligible for inclusion. Inclusion and exclusion criteria are specified in section 4.2 and 4.3 "*Inclusion criteria and exclusion criteria*". Pregnant or breast feeding women will be excluded from this study. Women with childbearing potential are eligible for inclusion, but should use adequate contraception for at least five months after eculizumab treatment. In our experience, we do not remember any patient getting pregnant within 5 months after SAH (probably related to the impact of the disease).

<u>h. Interaction with other products</u>

No official interaction studies have been performed. The website drugbank.ca reports the following medications to possibly interact with eculizumab: Possible interactions with other products include Belimumab, Denosumab, Fingolimod, Leflunomide, Natalizumab, Pimecrolimus, Roflumilast, Tacrolimus, Sipuleucel-T, Tofacitinib, and Trastuzumab.[44]

Patients who are using any of the aforementioned medicines are not eligible for inclusion in the CLASH trial.

i. Predictability of effect

In a previous study with eculizumab in patients with neuromyelitis optica, a 55% difference in CSF free C5 concentration was found after six weeks between the intervention and control group (eculizumab or no treatment). Our primary outcome will be C5a concentration in CSF after 48-72 hours. We expect that a decrease in C5a concentration will result in a decrease in brain injury, resulting in improved functional outcome.

j. Can effects be managed?

No antidote or antagonist is available for eculizumab. Patients will receive the first infusion of eculizumab on the emergency medicine department, ICU, neurological/neurosurgical HCU, or neurological/neurosurgical MCU and will be continuously monitored. Patients with an MC or ICU indication will also be continuously monitored during subsequent infusions with eculizumab. Routine SAH treatment on the neurological/neurosurgical HCU neurological/neurosurgical MCU or ICU includes GCS evaluation every two hours and temperature measurements every 12 hours at least for the first 24 hours and if necessary monitoring of inflammatory parameters. The medical staff is instructed to be alert on a possible infections, specifically on meningococcal meningitis and sepsis. In case the patient complains of any symptoms indicative of (meningitis) infection, further diagnostics will be completed and if necessary the appropriate antibiotics will be administered. Patients on the neurological/neurosurgical HCU, neurological/neurosurgical MCU or ICU with a decreased level of consciousness will be closely monitored according to standard SAH care also after 24 hours (unless the treating physicians deems it safe to decrease the frequency of monitoring). It is expected that if an infection develops, that this will be noted promptly due to the intensive monitoring and that antibiotics can be rapidly administered.

### 13.2 Synthesis

*Current indications*

Eculizumab is approved for treatment of patients with PNH and aHUS. Recently, refractory generalized myasthenia gravis in patients who are anti-acetylcholine receptor (AChR) antibody-positive has been approved as a new indication by the EMA. Eculizumab has been used in clinical practice since 2007 and is generally considered safe. It is currently used at the UMCU for children with PNH and adults with aHUS.

*Risks & measurements taken to reduce risks*

The most common side effect is headache and among the meningococcal infections the most SAE is meningococcal sepsis. Due to its working mechanism, patients who are treated with eculizumab are at increased risk of infections, specifically infections with the meningococcus (*Neisseria meningitidis*) bacteria. To reduce the risk of infection with the meningococcus bacteria, patients in the intervention group will receive four weeks of prophylactic ciprofloxacin. Patients in the intervention group with a central line or an external lumbar or ventricular drain and a positive yeast culture will also receive antifungal prophylaxis to decrease the risk of infection with a fungus. Multi-drug resistance and yeast carriership/colonization will be closely monitored by throat and rectal swaps. Patients with a known allergy to ciprofloxacin are excluded from participation in this study (4.3 '*Exclusion criteria*'). Immunocompromised patients or patients at high risk of antibiotic resistance are also excluded (4.3 '*Exclusion criteria*'). Patients in the intervention group will receive their first infusion of eculizumab at either the emergency department, neurological/neurosurgical HCU, neurological/neurosurgical MCU or ICU where continuous monitoring will be present. Medical staff will be informed about the potential increased risk of infection, specifically of meningococcal sepsis and asked to be alert on any indicative symptoms or signs. Frequent GCS evaluation, temperature monitoring and if necessary inflammatory parameters will allow for prompt detection of meningitis or meningococcal sepsis in patients with a decreased consciousness. If a CSF culture proven Neisseria meningitis or blood culture proven meningococcal sepsis develops, the appropriate antibiotics will be started immediately and treatment with eculizumab will be halted. If an allergic reaction occurs, depending on the severity, infusion will be slowed or halted. Patients with increased risk of AEs will be excluded (see section 4.3 *'Exclusion criteria'*). CSF sampling poses a possible infection risk. This risk will be reduced by working in sterile conditions when CSF sampling is performed. The other measurements performed (e.g. blood, brain MRI, neurological examination and questionnaires) pose a minimal risk to the patient and many are part of standard SAH care. Patients in the intervention group will receive safety instructions and a patient safety card before discharge. These patients will be asked to carry the patient safety card on their person until four weeks after ictus. To monitor the safety of the CLASH trial, an external DSMB will be established.

*Benefits*

Results from studies in a SAH mouse model seem promising: >40% decrease in brain injury was found in mice treated with C5 antibodies and also in C5aR knockout mice.[13] A study performed in patients with neuromyelitis optica found a decrease of 55% in C5 concentration after six weeks in the group treated with eculizumab compared to the control group.[17] By

decreasing the concentration of potent inflammatory mediators such as C5a and C5b, we expect a decrease in brain injury and thereby we expect to improve functional outcome.

*Conclusion*

Eculizumab is generally considered safe but serious side effects have been described. This study applies various measures to reduce risk associated with eculizumab treatment including prophylactic antibiotics, close monitoring, safety instructions, and a patient safety card. Also, incidence of meningococcal meningitis and sepsis have been very low in previous studies. Potential benefit include a decrease in brain injury and therefore a better prognosis. Taking together the side effects and potential benefits, it is expected that the overall benefits on neurological outcome outweigh the side effects and that these risks are therefore acceptable for the participants of the CLASH study.

## 14. REFERENCES

1. Nieuwkamp DJ, Setz LE, Algra A, Linn FHH, de Rooij NK, Rinkel GJE. Changes in case fatality of aneurysmal subarachnoid haemorrhage over time, according to age, sex, and region: a meta-analysis. *Lancet. Neurol.* 2009;8:635–42.
2. Johnston SC, Selvin S, Gress DR. The burden, trends, and demographics of mortality from subarachnoid hemorrhage. *Neurology*. 1998;50:1413–8.
3. Rosengart AJ, Schultheiss KE, Tolentino J, Macdonald RL. Prognostic Factors for Outcome in Patients With Aneurysmal Subarachnoid Hemorrhage. *Stroke*. 2007;38:2315–2321.
4. Broderick JP, Brott TG, Duldner JE, Tomsick T, Leach A. Initial and recurrent bleeding are the major causes of death following subarachnoid hemorrhage. *Stroke*. 1994;25:1342–1347.
5. Macdonald RL. Delayed neurological deterioration after subarachnoid haemorrhage. *Nat. Rev. Neurol.* 2013;10:44–58.
6. Vergouwen MD, Jong-Tjien-Fa A, Algra A, Rinkel GJ. Time trends in causes of death after aneurysmal subarachnoid hemorrhage: A hospital-based study. *Neurology*. 2015;86:59–63.
7. Zanier ER, Zangari R, Munthe-Fog L, Hein E, Zoerle T, Conte V, et al. Ficolin-3-mediated lectin complement pathway activation in patients with subarachnoid hemorrhage. *Neurology*. 2014;82:126–34.
8. Mack WJ, Ducruet AF, Hickman ZL, Garrett MC, Albert EJ, Kellner CP, et al. Early plasma complement C3a levels correlate with functional outcome after aneurysmal subarachnoid hemorrhage. *Neurosurgery*. 2007;61:255-60–1.
9. Kasuya H, Shimizu T. Activated complement components C3a and C4a in cerebrospinal fluid and plasma following subarachnoid hemorrhage. *J. Neurosurg.* 1989;71:741–6.
10. Pellettieri L, Nilsson B, Carlsson CA, Nilsson U. Serum immunocomplexes in patients with subarachnoid hemorrhage. *Neurosurgery*. 1986;19:767–71.
11. Yanamoto H, Kataoka H, Nakajo Y, Iihara K. The role of the host defense system in the development of cerebral vasospasm: analogies between atherosclerosis and subarachnoid hemorrhage. *Eur. Neurol.* 2012;68:329–43.
12. Cai J-Y, Sun J, Yu Z-Q. Serum mannose-binding lectin levels after aneurysmal subarachnoid hemorrhage. *Acta Neurol. Scand.* 2016;134:360–367.
13. van Dijk BJ, Meijers JCM, Kloek AT, Knaup VL, Rinkel GJE, Morgan P, et al. Complement component C5 contributes to brain injury after aneurysmal subarachnoid hemorrhage. *Unplublished Manuscr.*

14. Vergouwen MDI, Vermeulen M, van Gijn J, Rinkel GJE, Wijdicks EF, Muizelaar JP, et al. Definition of delayed cerebral ischemia after aneurysmal subarachnoid hemorrhage as an outcome event in clinical trials and observational studies: proposal of a multidisciplinary research group. *Stroke.* 2010;41:2391–2395.
15. Horan TC, Andrus M, Dudeck MA. CDC/NHSN surveillance definition of health care-associated infection and criteria for specific types of infections in the acute care setting. *Am. J. Infect. Control*. 2008;36:309–32.
16. de Rooij NK, Linn FHH, van der Plas JA, Algra A, Rinkel GJE. Incidence of Subarachnoid Hemorrhage: a systematic review with emphasis on region, age, genderand time trends. *Stroke*. 2007;27:1365–72.
17. Pittock SJ, Lennon VA, McKeon A, Mandrekar J, Weinshenker BG, Lucchinetti CF, et al. Eculizumab in AQP4-IgG-positive relapsing neuromyelitis optica spectrum disorders: An open-label pilot study. *Lancet Neurol.* 2013;12:554–562.
18. Rother RP, Rollins SA, Mojcik CF, Brodsky RA, Bell L. Discovery and development of the complement inhibitor eculizumab for the treatment of paroxysmal nocturnal hemoglobinuria. *Nat. Biotechnol.* 2007;25:1256–64.
19. European Medicines Agency, Soliris-EMEA/H/C/00791-II/0090/2009. Soliris : EPAR - Product Information. 2009;1–44. http://www.ema.europa.eu/docs/en_GB/document_library/EPAR_-_Product_Information/human/000791/WC500054208.pdf, July 2017.
20. Hofstee HMA, van Maaren MS, Postema HE, Tjwa DJT. Het Acute Boekje. 2017. https://www.hetacuteboekje.nl/hoofdstuk/anafylaxie, September 2017.
21. Brodsky RA, Young NS, Antonioli E, Risitano AM, Schrezenmeier H, Schubert J, et al. Multicenter phase 3 study of the complement inhibitor eculizumab for the treatment of patients with paroxysmal nocturnal hemoglobinuria. *Blood*. 2008;111:1840–1847.
22. Hillmen P, Young NS, Schubert J, Brodsky RA, Socié G, Muus P, et al. The Complement Inhibitor Eculizumab in Paroxysmal Nocturnal Hemoglobinuria. *N. Engl. J. Med.* 2006;355:1233–1243.
23. Legendre CM, Licht C, Muus P, Greenbaum LA, Babu S, Bedrosian C, et al. Terminal Complement Inhibitor Eculizumab in Atypical Hemolytic–Uremic Syndrome. *N. Engl. J. Med.* 2013;368:2169–2181.
24. Howard JF, Barohn RJ, Cutter GR, Freimer M, Juel VC, Mozaffar T, et al. A randomized, double-blind, placebo-controlled phase II study of eculizumab in patients with refractory generalized myasthenia gravis. *Muscle Nerve*. 2013;48:76–84.
25. Laban KG, Rinkel GJE, Vergouwen MDI. Nosocomial Infections after Aneurysmal Subarachnoid Hemorrhage: Time Course and Causative Pathogens. *Int. J. Stroke*. 2015;10:763–766.

26. European Medicines Agency, EMA/00791/2007. Soliris : EPAR - Scientific Discussion. 2007;1–41. http://www.ema.europa.eu/docs/en_GB/document_library/EPAR_-_Scientific_Discussion/human/000791/WC500054212.pdf, July 2017.
27. Connolly ES, Rabinstein AA, Carhuapoma JR, Derdeyn CP, Dion J, Higashida RT, et al. Guidelines for the management of aneurysmal subarachnoid hemorrhage: a guideline for healthcare professionals from the American Heart Association/american Stroke Association. *Stroke.* 2012;43:1711–37.
28. Steiner T, Juvela S, Unterberg A, Jung C, Forsting M, Rinkel GJ. European Stroke Organization guidelines for the management of intracranial aneurysms and subarachnoid haemorrhage. *Cerebrovasc. Dis.* 2013;35:93–112.
29. Hoefnagel D, Dammers R, Ter Laak-Poort MP, Avezaat CJJ. Risk factors for infections related to external ventricular drainage. *Acta Neurochir. (Wien).* 2008;150:209–14; discussion 214.
30. Kleyweg RP, Hertzberger LI, Carbaat PA. Less headache following lumbar puncture with the use of an atraumatic needle; double-blind randomized study. *Ned. Tijdschr. Geneeskd.* 1995;139:232–4.
31. Ruff RL, Dougherty JH. Complications of lumbar puncture followed by anticoagulation. *Stroke*. 12:879–81.
32. Evans RW. Complications of lumbar puncture. *Neurol. Clin.* 1998;16:83–105.
33. Samdani A, Garonzik IM, Zahos P. Subdural hematoma after diagnostic lumbar puncture. *Am. J. Emerg. Med.* 2004;22:316–7.
34. Adler MD, Comi AE, Walker AR. Acute hemorrhagic complication of diagnostic lumbar puncture. *Pediatr. Emerg. Care*. 2001;17:184–8.
35. Pumberger M, Memtsoudis SG, Stundner O, Herzog R, Boettner F, Gausden E, et al. An Analysis of the Safety of Epidural and Spinal Neuraxial Anesthesia in More Than 100,000 Consecutive Major Lower Extremity Joint Replacements. *Reg. Anesth. Pain Med.* 2013;38:515–519.
36. Niedermüller U, Trinka E, Bauer G. Abducens palsy after lumbar puncture. *Clin. Neurol. Neurosurg.* 2002;104:61–3.
37. Baer ET. Post-dural puncture bacterial meningitis. *Anesthesiology*. 2006;105:381–93.
38. Nasreddine ZS, Phillips NA, Bédirian V, Charbonneau S, Whitehead V, Collin I, et al. The Montreal Cognitive Assessment, MoCA: A Brief Screening Tool For Mild Cognitive Impairment. *J. Am. Geriatr. Soc.* 2005;53:695–699.
39. Schweizer TA, Al-Khindi T, Macdonald RL. Mini-Mental State Examination versus Montreal Cognitive Assessment: Rapid assessment tools for cognitive and functional outcome after aneurysmal subarachnoid hemorrhage. *J. Neurol. Sci.* 2012;316:137–

140.

40. Wong GKC, Lam SW, Ngai K, Wong A, Siu D, Poon WS, et al. Cognitive domain deficits in patients with aneurysmal subarachnoid haemorrhage at 1 year. *J. Neurol. Neurosurg. Psychiatry*. 2013;84:1054–1058.
41. Janssen MF, Pickard AS, Golicki D, Gudex C, Niewada M, Scalone L, et al. Measurement properties of the EQ-5D-5L compared to the EQ-5D-3L across eight patient groups: a multi-country study. *Qual. Life Res.* 2013;22:1717–27.
42. Herdman M, Gudex C, Lloyd A, Janssen M, Kind P, Parkin D, et al. Development and preliminary testing of the new five-level version of EQ-5D (EQ-5D-5L). *Qual. Life Res.* 2011;20:1727–36.
43. Dorman PJ, Slattery J, Farrell B, Dennis MS. A randomised comparison of the EuroQol and Short Form-36 after stroke. *BMJ*. 1997;315:461.
44. Law V, Knox C, Djoumbou Y, Jewison T, Guo AC, Liu Y, et al. DrugBank 4.0: shedding new light on drug metabolism. *Nucleic Acids Res.* 2014;42:D1091–D1097.
45. DAMOCLES Study Group, NHS Health Technology Assessment Programme P, Manns B, Ghali W, Quan H, Guyatt G. A proposed charter for clinical trial data monitoring committees: helping them to do their job well. *Lancet (London, England)*. 2002;365:711–22.
46. McHugh GS, Butcher I, Steyerberg EW, Marmarou A, Lu J, Lingsma HF, et al. A simulation study evaluating approaches to the analysis of ordinal outcome data in randomized controlled trials in traumatic brain injury: results from the IMPACT Project. *Clin. Trials*. 2010;7:44–57.
47. Roos YB, de Haan RJ, Beenen LFM, Groen RJM, Albrecht KW, Vermeulen M. Complications and outcome in patients with aneurysmal subarachnoid haemorrhage: a prospective hospital based cohort study in The Netherlands. *J. Neurol. Neurosurg. & Psychiatry*. 2000;68:337-341.
48. Schmidt JM, Wartenberg KE, Fernandez A, Claassen J, Rincon F, Ostapkovich ND, et al. Frequency and clinical impact of asymptomatic cerebral infarction due to vasospasm after subarachnoid hemorrhage. *J. Neurosurg.* 2008;109:1052–1059.
49. Dobrina A, Pausa M, Fischetti F, Bulla R, Vecile E, Ferrero E, et al. Cytolytically inactive terminal complement complex causes transendothelial migration of polymorphonuclear leukocytes in vitro and in vivo. *Blood*. 2002;99:185–92.

## Appendix 1: Algorithm for treatment of anaphylaxis (in Dutch)

- Lage bloeddruk (systolisch < 90 mmHg of 30% systolische afname) < 2uur na blootstelling aan waarschijnlijk allergeen

Of:

- Acuut ontstaan van 2 of meer van de items hieronder:
  - Gegeneraliseerde jeuk, urticaria, erytheem of angio-oedeem
  - Kuchen/keelschrapen, piepen, stridor, moeite met ademhalen
  - Lage bloeddruk (systolisch < 90 mmHg of 30% systolische afname)
  - Misselijkheid, braken, buikkrampen of diarree

**Ja** →

Anafylaxie of ernstige (allergische) reactie

N.B.: Belemmerde luchtweg door acuut angio-oedeem van tong of larynx behoeft een andere behandeling (zie beleid)

**Nee** →

Milde (allergische) reactie of allergische reactie onwaarschijnlijk (zie beleid)

<u>Stap 1</u>

-Verwijder of stop toediening allergeen

-Beoordeel patiënt volgens A,B,C methode

-Vraag hulp, indien nodig, voor ademweg en/of hemodynamiek (bv. crashteam)

<u>Stap 2</u>

-Dien 0,5 mg adrenaline intramusculair (i.m.) toe (laterale zijde bovenbeen)

-Bij (verdenking) bronchospasme: salbutamol 5 mg vernevelen

-Geef 2 mg clemastine (Tavegil) i.m. of i.v.

-Geef 8 mg dexamethason (of equivalente dosis glucocorticoïd) i.v.

<u>Stap 3</u> *(indien geen vlot herstel binnen 5 minuten)*

-verander positie:

- Bij hypotensie: leg patiënt plat neer
- Bij belemmerde ademhaling: rechtop
- Bij misselijkheid: hoofd opzij of voorover

-Dien nogmaals adrenaline 0,5 mg i.m. toe

-Bij hypotensie: geef fluid challenge 500 ml NaCl 0,9% in 15 min en herhaal zo nodig

<u>Stap 4</u> *(indien geen herstel)*

-Vervolgbehandeling MC/IC

-Bij gebruik beta-blokker: overweeg toediening glucagon 1 mg i.v. in 5 min. gevolgd door 5-15 microg/min i.v.

-Start adrenaline perfusor 1 microg/min i.v. en titreer tot hemodynamisch stabiel (max 15 microg/min)

<u>Indien herstel op SEH:</u>

-Beoordeel patiënt regelmatig volgens A,B,C methode

-Duur observatie:

- Altijd minimaal 4 uur
- Minimaal 12 uur bij initiële hypotensie, als klachten onvoldoende over zijn na 4 uur, als er geen of inadequate medische hulp is thuis

-Zie nazorg onder paragraaf beleid

## Appendix 2: Drug Accountability Form

UMC Utrecht

Executive Board

### Drug Accountability Form

| | | | |
|---|---|---|---|
| Study name | CLASH study | Protocol number (METC/ABR) | |
| PI name | Dr. M.D.I. Vergouwen | Sponsor/Verrichter | UMC Utrecht |
| Site name + number | | Name of drug + strength | Eculizumab, 1200 mg |

| Subject ID | Units[1] | # Lot/Batch Number | Amount Dispensed | Date Dispensed | Dispensed by (signature[2]) | Amount Returned | Date Returned | Returned to (signature[2]) |
|---|---|---|---|---|---|---|---|---|
| | | | | | | | | |
| | | | | | | | | |
| | | | | | | | | |
| | | | | | | | | |
| | | | | | | | | |
| | | | | | | | | |

[1] *Example: tablets, bottles, vials etc.*
[2] *Should be authorized on the Authorization Form*

## Appendix 3: NIHSS score (in Dutch)

| **Item:** | **Omschrijving:** | **Score:** |
|---|---|---|
| **1a Bewustzijn.** | 0 Alert.<br>1 Niet alert, maar wekbaar met een geringe stimulus.<br>2 Niet alert, moet herhaaldelijk gestimuleerd worden.<br>3 Coma (niet wekbaar). | … |
| **1b Vraag naar maand en leeftijd.** | 0 Beantwoordt beide correct.<br>1 Beantwoordt één van beide correct.<br>2 Beantwoordt niet of beide incorrect. | … |
| **1c Vraag de ogen te sluiten en vuist te maken.** | 0 Voert beide opdrachten correct uit.<br>1 Voert één van beide opdrachten correct uit.<br>2 Voert geen van beide opdrachten correct uit. | … |
| **2 Blikrichting/Oogbewegingen** | 0 Normaal.<br>1 Kijkt bij voorkeur naar één kant.<br>2 Dwangstand. | … |
| **3 Gezichtsvelden.** | 0 Lijken intact.<br>1 Gedeeltelijke gezichtsveld uitval/extinctie.<br>2 Complete halfzijdige gezichtsveld uitval.<br>3 Blind (bilaterale hemianopsie/corticale blindheid). | … |
| **4 Aangezichtsverlamming**<br>**(vraag wenkbrauwen op te trekken, ogen te sluiten en tanden te laten zien).** | 0 Normaal/symmetrisch.<br>1 Lichte asymmetrie, verstreken nasolabiaal-plooi. | … |

| | | |
|---|---|---|
| | 2 Totale of subtotale verlamming onderste gelaatshelft.<br>3 Complete verlamming van oog en mondspieren (een of beide zijden). | |
| **5a Motorische functie.**<br>**• Rechterarm.** | 0 Kan normaal arm uitstrekken (gedurende 10 sec).<br>1 Arm zakt uit (“drift”).<br>2 Enige beweging tegen de zwaartekracht.<br>3 Geen beweging tegen de zwaartekracht (wel aanspanning spieren).<br>4 Geen beweging.<br>9 Niet te testen (geef oorzaak aan; b.v. amputatie) . | … |
| **5b Motorische functie.**<br>**• Linkerarm.** | 0 Kan normaal arm uitstrekken (gedurende 10 sec).<br>1 Arm zakt uit (“drift”).<br>2 Enige beweging tegen de zwaartekracht.<br>3 Geen beweging tegen de zwaartekracht (wel aanspanning spieren).<br>4 Geen beweging.<br>9 Niet te testen (geef oorzaak aan; b.v. amputatie). | … |
| **6a Motorische functie**<br>**• Rechterbeen** | 0 Kan normaal been optillen (30° gedurende 5 sec).<br>1 Been zakt uit (“drift”).<br>2 Enige beweging tegen de zwaartekracht.<br>3 Geen beweging tegen de | … |

| | | |
|---|---|---|
| | zwaartekracht (wel aanspanning spieren).<br>4 Geen beweging.<br>9 Niet te testen (geef oorzaak aan; b.v. amputatie). | |
| **6b Motorische functie.**<br>**• Linkerbeen.** | 0 Kan normaal been optillen (30° gedurende 5 sec).<br>1 Been zakt uit (“drift”).<br>2 Enige beweging tegen de zwaartekracht.<br>3 Geen beweging tegen de zwaartekracht (wel aanspanning spieren).<br>4 Geen beweging.<br>5 Niet te testen (geef oorzaak aan; b.v. amputatie). | … |
| **7 Ataxie.** | 0 Niet aanwezig.<br>1 Aanwezig in één arm of één been .<br>2 Aanwezig in twee of meer ledematen. | … |
| **8 Sensibiliteit**<br>**(pijnzin links en rechts vergelijken).** | 0 Normaal.<br>1 Verminderd.<br>2 Afwezig. | … |
| **9 Taal**<br>**(laat een plaatje beschrijven en voorwerpen benoemen, zinnen nazeggen)** | 0 Geen afasie.<br>1 Lichte tot matig ernstige afasie.<br>2 Ernstige afasie.<br>3 Mutistisch of globale afasie. | … |
| **10 Spraakstoornis/Dysarthrie**<br><br>**(woorden laten oplezen)** | 0 Normale articulatie.<br>1 Onduidelijke spraak.<br>2 Ernstige dysarthrie/anarthrie.<br>9 Niet te testen (tube). | … |
| **11 Extinctie en Inattentie**<br>**(visuele en tactiele prikkels tege- lijk links en** | 0 Normaal (niet aanwezig).<br>1 Inattentie of extinctie voor | … |

| | | |
|---|---|---|
| **rechts aanbieden)** | één soort prikkel.<br>2 Ernstige hemi-inattentie voor beide prikkels. | |
| | **Totaalscore:** | … |

**Uitleg NIHSS score:**

**<u>1a Bewustzijn:</u>**

De onderzoeker moet een reactie kiezen, ook als een respons bemoeilijkt wordt door obstakels zoals een endotracheale tube, taal barrière, letsel aan mond-keel holte en/of luchtwegen, of verband aldaar. Drie punten worden alleen toegekend als de patiënt niet beweegt (anders dan reflexmatig) in reactie op pijnprikkels.

**<u>1b Vragen:</u>**

Vraag de patiënt naar de maand en zijn/haar leeftijd, het antwoord moet correct zijn – er worden geen punten gegeven als het antwoord in de buurt zit. Patiënten met afasie en patiënten met een gedaald bewustzijn, die de vragen niet begrijpen, scoren 2 punten. Patiënten die niet kunnen praten vanwege een endotracheale tube, letsel aan mond-keel holte en/of luchtwegen, ernstige dysarthrie door welke oorzaak dan ook, taalbarrière, of ieder ander probleem **niet** secundair aan afasie scoren 1 punt. Het is belangrijk dat alleen het initiële antwoord gescoord wordt en dat de onderzoeker de patiënt niet helpt middels verbale of non-verbale hints.

**<u>1c Opdrachten:</u>**

De patiënt wordt gevraagd de ogen te openen en te sluiten en dan te knijpen en los te laten met de niet-paretische hand. Vervang de opdracht door een andere enkelvoudige opdracht als de handen niet gebruikt kunnen worden. Er worden punten gescoord als er een eenduidige poging wordt gedaan, maar niet wordt afgemaakt door zwakte. Als de patiënt niet reageert op de opdracht, moet de opdracht voorgedaan worden (pantomime) en het resultaat gescoord worden (voert geen, één of twee opdrachten uit). Patiënten met een trauma, amputatie of andere fysieke beperkingen moeten passende enkelvoudige opdrachten krijgen. Alleen de eerste poging wordt gescoord.

**<u>2 Blikrichting/Oogbewegingen:</u>**

Alleen de horizontale oogbewegingen worden getest. Willekeurige of reflexmatige (oculocephale) oogbewegingen worden gescoord, maar calorische prikkels worden niet verricht. Als de patiënt een dwangstand van de ogen heeft, die wordt opgeheven bij

willekeurige of reflexmatige activiteit, is de score 1 punt. Als de patiënt een geïsoleerde, perifere parese heeft van de hersenzenuw III, IV of VI, is de score 1 punt. Oogbewegingen zijn te testen bij afasie patiënten. Patiënten met oculair trauma, verband, preëxistente blindheid of andere stoornissen van het gezichtsvermogen of de gezichtsvelden moeten getest worden met reflexmatige bewegingen en er moet een keuze gemaakt worden door de onderzoeker. Het verkrijgen van oogcontact en dan naar de andere zijde van de patiënt gaan kan de aanwezigheid van een partiële blikparese al dan niet aantonen.

**3 Gezichtsvelden:**

Gezichtsvelden (onderste en bovenste kwadrant) worden getest met confrontatie, door vingers te laten tellen of met de dreigreflex.. Patiënten moeten aangemoedigd worden recht vooruit te kijken, maar als de patiënt naar de zijde kijkt van de bewegende vingers kan dit als normaal gescoord worden. Als er een unilaterale blindheid of enucleatie (bv. bij cataract) is, worden de gezichtsvelden in het andere oog gescoord. 1 punt wordt alleen gescoord als er een duidelijke asymmetrie is, inclusief kwadrantanopsie. Indien een patiënt blind is, ongeacht de oorzaak, worden 3 punten gescoord. Als er bij dubbelzijdige, gelijktijdige stimulatie extinctie is, wordt 1 punt gescoord en dit resultaat wordt gebruikt bij het antwoord op vraag 11.

**4 Aangezichtsverlamming:**

Vraag de patiënt, of maak gebruik van pantomime, om de patiënt de tanden te laten zien, de wenkbrauwen op te tillen en de ogen te sluiten. Bij een patiënt die matig alert is of de opdracht niet begrijpt scoort u de symmetrie van de grimas met een pijnprikkel. Indien het gelaat verbonden is, of de patiënt heeft een endotracheale tube, tape of andere fysieke barrière voor het gelaat die de beoordeling belemmert, dan moet deze zoveel mogelijk worden verwijderd.

**5 & 6 Motorisch functie armen en benen:**

De extremiteit moet in de juiste positie worden gepositioneerd: de armen (met de handpalmen naar beneden) in zittende of staande positie 90 graden vooruit, of 45 graden in liggende positie, en de benen 30 graden, gestrekt (altijd liggend). ‘Uitzakken’ wordt gescoord als de armen binnen 10 en de benen binnen 5 seconden uitzakken. De afasiepatiënt wordt gestimuleerd door middel van een aanmoedigende stem en pantomime, maar niet met een pijnprikkel. Elke extremiteit wordt om de beurt getest, te beginnen met de niet-paretische arm. Alleen in het geval van een amputatie, of arthrodese in schouder of heup mag een ‘9’ gescoord worden, de onderzoeker moet dan altijd een verklaring opschrijven.

**7 Ataxie van de ledematen**

Dit item is bedoeld om een unilateraal cerebellair syndroom aan te tonen. Test met de ogen open. Zorg dat er bij gezichtsvelduitval, in het intacte gezichtsveld getest wordt. De top- neus proef en de knie-hak proef moeten beiderzijds worden uitgevoerd. Ataxie wordt alleen gescoord als deze niet verklaard wordt door een parese. Ataxie wordt niet gescoord indien een patiënt de opdracht niet begrijpt of een paralyse heeft. Alleen in het geval van een amputatie of arthrodese kan een '9' gescoord worden; de onderzoeker moet in dat geval een verklaring opschrijven. Indien de patiënt blind is, test men de ataxie aan de armen door de patiënt met de arm vanuit gestrekte positie de vinger op de neus te laten zetten.

**8 Sensibiliteit**

Het aangeven van gevoel, het grimasseren op een speldenprik, of terugtrekken op pijnprikkel bij een afasie patiënt en patiënten met een gedaald bewustzijn. Alleen sensibiliteitsverlies als gevolg van de beroerte wordt als abnormaal gescoord en de onderzoeker dient zoveel mogelijk lichaamsgebieden [armen (niet handen), benen, romp en gezicht] te testen als nodig is om hemisensibiliteitsverlies op te sporen. 2 punten worden gescoord als er een ernstig of totaal sensibiliteitsverlies kan worden aangetoond. Afasie patiënten en patiënten met een gedaald bewustzijn zullen daarom meestal 0 of 1 punt scoren. Patiënten met een hersenstam lokalisatie van de beroerte, met bilateraal sensibiliteitsverlies, scoren 2 punten. Als de patiënt niet reageert en een quadriplegie heeft, scoort u 2 punten. Patiënten in coma krijgen, arbitrair, 2 punten bij dit item.

**9 Taal**

Een belangrijke indruk van het taalbegrip wordt verkregen tijdens de uitvoering van het voorafgaande onderzoek. De patiënt wordt gevraagd om te beschrijven wat er op bijgevoegde afbeelding ('koekjesdief') gebeurt, om de afgebeelde voorwerpen te benoemen en de zinnen voor te lezen. Het begrip wordt beoordeeld aan de hand van de reacties op deze testen en op alle opdrachten in het voorgaand neurologisch onderzoek. Als visusstoornissen interfereren met het testonderzoek, vraag dan of de patiënt voorwerpen kan benoemen die u bij de patiënt in de hand legt, vraag te herhalen en vraag een stukje te vertellen. De geïntubeerde patiënt moet gevraagd worden te schrijven. De patiënt in coma (vraag 1a=3) zal, arbitrair, 3 punten krijgen bij deze vraag. De onderzoeker moet een score kiezen voor de patiënt met een gedaald bewustzijn, of patiënten die beperkt meewerken. Een score van 3 punten wordt echter alleen gegeven als een patiënt mutistisch is en geen eenvoudige opdrachten uitvoert.

**10 Spraakstoornis/Dysarthrie**

Als dit van een patiënt kan worden verwacht, moet er een indruk over de spraak worden verkregen door de patiënt een woordenlijst te laten lezen of enige woorden te herhalen. Als de patiënt een ernstige afasie heeft, moet de duidelijkheid van de articulatie in de spontane taal beoordeeld worden. Alleen als de patiënt geïntubeerd is, of een andere fysieke barrière heeft om te spreken, mag de score van 9 punten gegeven worden en dient de onderzoeker een duidelijke uitleg te geven voor het toekennen van deze score. Vertel de patiënt niet waarom hij/zij getest wordt.

**11 Extinctie & inattentie**

Noodzakelijke informatie om neglect te diagnosticeren kan al zijn verkregen tijdens het voorgaand onderzoek. Als een patiënt ernstige visusstoornissen heeft waardoor tweezijdig simultaan visueel testen niet mogelijk is en het simultaan testen van de tast normaal is, wordt de score van 0 punten (=normaal) toegekend. Als de patiënt een afasie heeft maar naar beide kanten zijn/haar aandacht kan richten, wordt er ook 0 punten gescoord. De aanwezigheid van visueel ruimtelijk neglect of een anosognosie wordt ook als afwijkend beschouwd. Omdat de afwijking alleen gescoord wordt als deze aanwezig is, wordt dit item nooit als ‘niet te testen’ gescoord.

## Appendix 4: WFNS score (in Dutch)

| WFNS | GCS | Motore uitval |
| --- | --- | --- |
| I | 15 | Afwezig |
| II | 13-14 | Afwezig |
| III | 13-14 | Aanwezig |
| IV | 7-12 | Afwezig/aanwezig |
| V | 3-6 | Afwezig/Aanwezig |

## Appendix 5: MoCA score (in Dutch)

**Nederlandse versie**
**MONTREAL COGNITIVE ASSESSMENT (MOCA)**

Geboortedatum:
Jaren opleiding: Naam:
Geslacht: Datum:

**VISUOSPATIEEL/EXECUTIEF** — PUNTEN

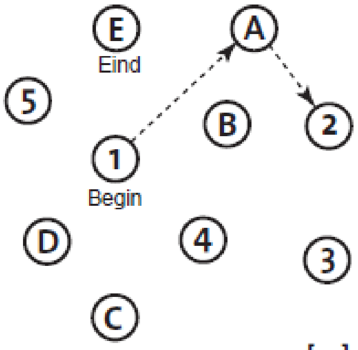


[ ]

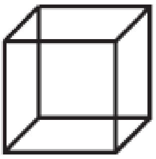

Kopieer de kubus [ ]

Teken een klok (tien over elf) (3 punten)

[ ] Omtrek [ ] Cijfers [ ] Wijzers — __/5

**BENOEMEN**

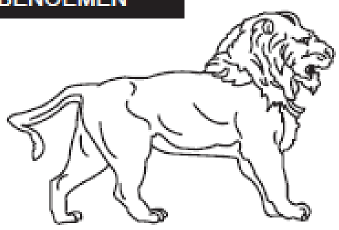
[ ]

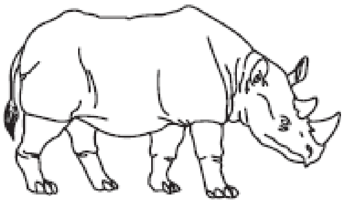
[ ]

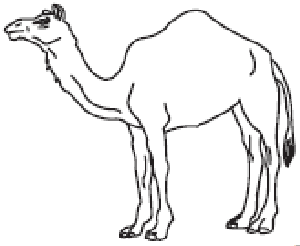
[ ] — __/3

**GEHEUGEN**

Lees de woorden op, proefpersoon moet ze nazeggen. Neem 2 maal af. Laat ze na 5 min. opnieuw opnoemen.

| | GEZICHT | FLUWEEL | KERK | MADELIEF | ROOD |
|---|---|---|---|---|---|
| 1e afname | | | | | |
| 2e afname | | | | | |

Geen punten

**AANDACHT**

Lees de rij cijfers op (1 cijfer/sec). Proefpersoon moet ze in dezelfde volgorde nazeggen [ ] 2 1 8 5 4
Proefpersoon moet ze in omgekeerde volgorde nazeggen [ ] 7 4 2 — __/2

Lees de rij letters op. De proefpersoon moet bij iedere letter A met zijn hand op de tafel tikken. Geen punten bij ≥ 2 ft
[ ] F B A C M N A A J K L B A F A K D E A A A J A M O F A A B — __/1

Serieel 7 aftrekken, beginnend bij 100 [ ] 93 [ ] 86 [ ] 79 [ ] 72 [ ] 65
4 of 5 goed: **3 pt** 2 of 3 goed: **2 pt** 1 goed: **1 pt** 0 goed: **0 pt** — __/3

**TAAL**

Zeg na: Ik weet alleen dat Jan vandaag geholpen zou worden. [ ]
De kat verstopte zich altijd onder de bank als er honden in de kamer waren. [ ] — __/2

Fluency: Noem binnen één minuut zo veel mogelijk woorden die beginnen met de letter D [ ] (N ≥ 11 woorden) — __/1

**ABSTRACTIE**

Overeenkomst tussen bijv. banaan en sinaasappel = fruit [ ] trein-fiets [ ] horloge-liniaal — __/2

**UITGESTELDE RECALL**

| | | GEZICHT | FLUWEEL | KERK | MADELIEF | ROOD | |
|---|---|---|---|---|---|---|---|
| | Woorden moeten herinnerd worden zonder cue | [ ] | [ ] | [ ] | [ ] | [ ] | Punten alleen voor recall zonder cue — __/5 |
| Optioneel | Categoriecue | | | | | | |
| | Meerkeuzecue | | | | | | |

**ORIËNTATIE**

[ ] Datum [ ] Maand [ ] Jaar [ ] Dag [ ] Locatie [ ] Plaats — __/6

© Z.Nasreddine MD 2004, translated to Dutch by P.L.J. Dautzenberg and J.F.M. de Jonghe
www.mocatest.org

Normaal ≥ 26 / 30 **TOTAAL** __/30
Tel er 1 pt bij op indien ≤ 12 jr opleiding

## Appendix 6: EQ-5D-5L questionnaire (in Dutch)

The EuroQoL 5-dimensions 5-level (EQ-5D-5L) questionnaire is a standardized measure of health outcome.

Zet bij iedere groep in de lijst hieronder een kruisje in het hokje dat het best past bij uw gezondheid VANDAAG.

MOBILITEIT

- Ik heb geen problemen met lopen ❑
- Ik heb een beetje problemen met lopen ❑
- Ik heb matige problemen met lopen ❑
- Ik heb ernstige problemen met lopen ❑
- Ik ben niet in staat om te lopen ❑

ZELFZORG

- Ik heb geen problemen met mijzelf wassen of aankleden ❑
- Ik heb een beetje problemen met mijzelf wassen of aankleden ❑
- Ik heb matige problemen met mijzelf wassen of aankleden ❑
- Ik heb ernstige problemen met mijzelf wassen of aankleden ❑
- Ik ben niet in staat mijzelf te wassen of aan te kleden ❑

DAGELIJKSE ACTIVITEITEN ***(bijv. werk, studie, huishouden, gezins- en vrijetijdsactiviteiten)***

- Ik heb geen problemen met mijn dagelijkse activiteiten ❑
- Ik heb een beetje problemen met mijn dagelijkse activiteiten ❑
- Ik heb matige problemen met mijn dagelijkse activiteiten ❑
- Ik heb ernstige problemen met mijn dagelijkse activiteiten ❑
- Ik ben niet in staat mijn dagelijkse activiteiten uit te voeren ❑

PIJN/ONGEMAK

- Ik heb geen pijn of ongemak ❑
- Ik heb een beetje pijn of ongemak ❑
- Ik heb matige pijn of ongemak ❑
- Ik heb ernstige pijn of ongemak ❑
- Ik heb extreme pijn of ongemak ❑

ANGST/SOMBERHEID

- Ik ben niet angstig of somber ❑
- Ik ben een beetje angstig of somber ❑
- Ik ben matig angstig of somber ❑
- Ik ben erg angstig of somber ❑
- Ik ben extreem angstig of somber ❑

De beste gezondheid die u zich kunt voorstellen

- We willen weten hoe goed of slecht uw gezondheid VANDAAG is.
- Deze meetschaal loopt van 0 tot 100.
- 100 staat voor de beste gezondheid die u zich kunt voorstellen.
  0 staat voor de slechtste gezondheid die u zich kunt voorstellen.
- Markeer een X op de meetschaal om aan te geven hoe uw gezondheid VANDAAG is.
- Noteer het getal waarbij u de X heeft geplaatst in onderstaand vakje.

UW GEZONDHEID VANDAAG = [ ]

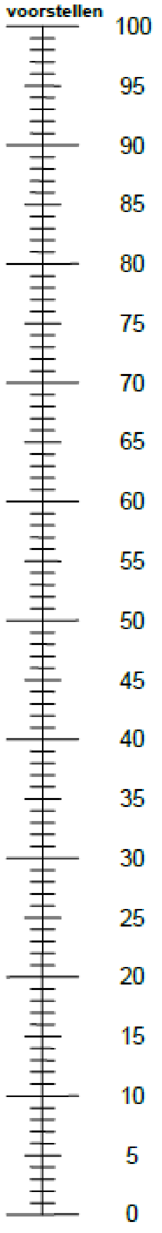


De slechtste gezondheid die u zich kunt voorstellen'


*Netherlands (Dutch) © 2009 EuroQol Group. EQ-5D™ is a trade mark of the EuroQol Group*

## Appendix 7: Questionnaire on AEs and SAEs (in Dutch)

### *CLASH studie*

---

### Vragenlijst over mogelijke klachten na de hersenvliesbloeding

Geachte meneer/mevrouw,
Wij vragen u vriendelijk om de volgende vragen in te vullen. De vragenlijst is bedoeld om te kijken naar mogelijke klachten die u ervaren heeft **tot vier weken** na de hersenvliesbloeding.

Heeft u tot **vier weken na** de hersenvliesbloeding last gehad van klachten die u voor de hersenvliesbloeding niet had?

……………………………………………

*Indien er sprake is van meerdere klachten zal de onderzoeker u vragen om voor elke klacht onderstaande vragen in te vullen. De onderzoeker zal u meerdere vragenlijsten geven om elke klacht apart in te vullen.

Indien ja, kunt u de klacht omschrijven?

……………………………………………

Op welke datum is de klacht begonnen (inclusief tijdstip)?

…………………………………………….

Zijn de symptomen behandeld met medicatie en indien ja wanneer?

…………………………………………….

Bent u weer volledig hersteld?

- o Ja
- o Nee

Indien ja: op welke datum was u weer volledig hersteld (inclusief tijdstip)?

…………………………………………….

Indien nee: wat is/zijn de beperking(en) die u hiervan ondervindt in het dagelijks leven?

…………………………………………….

Hartelijk dank voor het invullen van de vragenlijst!

## Appendix 8: mRS score (in Dutch)

| mRS score | |
|---|---|
| **Omschrijven** | **Score** |
| Geen symptomen. | 0 |
| Geen significante handicap, ondanks aanwezigheid van symptomen; kan alle dagelijks activiteiten en taken uitvoeren. | 1 |
| Lichte handicap; niet in staat om alle voorgaande activiteiten uit te voeren, maar kan eigen zaken regelen zonder hulp. | 2 |
| Matige handicap; behoeft enige hulp, maar is in staat om zonder hulp te lopen. | 3 |
| Matig zware handicap; niet in staat om zonder hulp te lopen en te voorzien in de eigen lichamelijk verzorging zonder hulp. | 4 |
| Ernstige handicap; bedgebonden, incontinent en behoeft constante verzorging en aandacht. | 5 |
| Overleden. | 6 |
| **Totaal score** | |

## Appendix 9: AE report form

### Adverse Event Form

| Study name | CLASH study | Sponsor | UMC Utrecht |
|---|---|---|---|
| Protocol number (METC/ABR) | | Subject ID | |

Has the participant had any Adverse Events* during this study? ☐ Yes ☐ No *(If yes, please list all Adverse Events below)*

| Severity | Study Intervention Relationship | Action Taken Regarding Study Procedure(s) | Outcome of Adverse Event | Expected | Meets criteria Serious Adverse Event |
|---|---|---|---|---|---|
| 1 = Mild<br>2 = Moderate<br>3 = Severe | 1 = Definitely related<br>2 = Probably related<br>3 = Possibly related<br>4 = Unlikely related<br>5 = Not related<br>6 = Not assessable | 1 = None<br>2 = Discontinued permanently<br>3 = Discontinued temporarily<br>4 = Reduced Dose<br>5 = Increased Dose<br>6 = Delayed Dose | 1 = Resolved, No Sequel<br>2 = AE still present- no treatment<br>3 = AE still present-being treated<br>4 = Residual effects present-not treated<br>5 = Residual effects present- treated<br>6 = Death<br>7 = Unknown | 1 = Yes<br>2 = No | 1 = Yes<br>2 = No<br>(If yes, complete SAE form) |

| Description Adverse Event | Start Date | Stop Date | Severity | Relationship to Study Procedure(s) | Action Taken | Outcome of Adverse Event | Expected? | Serious Adverse Event? | Initials Principal Investigator |
|---|---|---|---|---|---|---|---|---|---|
| 1. | | | | | | | | | |
| 2. | | | | | | | | | |
| 3. | | | | | | | | | |

* An Adverse Event is any undesirable experience occurring to a subject during the study, whether or not considered related to the investigational product, trial procedure or the experimental intervention.

## Appendix 10: SAE report form

# *SERIOUS ADVERSE EVENT FORM*

UMC Utrecht

| | |
|---|---|
| **Protocol No** ***(METC/ABR)*****:** | **EudraCT No** ***(if applicable)*****:** |
| **Study Title (short):** CLASH study | **Date of Report** ***(dd/mm/yy)*****:** |
| **Subject ID:** | **Year of birth**: |

***SAEs* will be reported through the web portal ToetsingOnline to the accredited METC that approved the protocol, within 7 days of first knowledge for SAEs that result in death or are life threatening followed by a period of maximum of 8 days to complete the initial preliminary report. *With the expectation of the SAEs described in section 9.2.2.***

| | |
|---|---|
| **1. Date of Report:** *(dd/mm/yy)* ____/____/____ | **2. Report Type:** ☐ Initial ☐ Follow-up ☐ Final |
| **3. Principal Investigator** (local)**:** | Name:<br>Institute: Office Phone:<br>Fax: E-mail: |
| **4. Date of Onset Serious Adverse Event:** (dd/mm/yy) | ____/____/_____ |
| **5. Brief description of Subject:** | Gender: M/F<br>Diagnosis: |

| |
|---|
| **6. Event/Reaction:** |

| |
|---|
| **7. Describe event:**<br>*(A summary of signs and symptoms, diagnosis, treatment of event, concurrent treatment, other relevant medical history, including re-challenge details if applicable. Please include the point in the study at which the event occurred.)* |

**8. Category of the serious adverse event:**

☐ death
☐ life-threatening
☐ hospitalization-initial or prolonged
☐ disability/incapacity
☐ congenital anomaly/birth defect
☐ required intervention to prevent all of the aforementioned situations

**9. Relationship of serious adverse event to the investigational medicinal product, the investigational medical device or the investigational treatment:**

| | Investigational Treatment | |
|---|---|---|
| ☐ 1 = unrelated | Expected | ☐ SAE |
| | Unexpected | ☐ SAE |
| ☐ 2 = unlikely | Expected | ☐ SAE |
| | Unexpected | ☐ SAE |
| ☐ 3 = possible | Expected | ☐ SAR |
| | Unexpected | ☐ SUSAR |
| ☐ 4 = probable | Expected | ☐ SAE |
| | Unexpected | ☐ SAE |
| ☐ 5 = definite | Expected | ☐ SAR |
| | Unexpected | ☐ SUSAR |

*Expected: The event is known, i.e. reported in earlier research as possibly related.*
*Unexpected:The event is unknown, i.e. not reported in earlier research.*

**10. Action taken with investigational product or the experimental treatment:**

☐ None
☐ Discontinued temporarily
☐ Discontinued
☐ Dose temporarily reduced
☐ Dose reduced

| **11. What is the outcome of the SAE?** | **12. Date event resolved** ____/____/_____ *(dd/mm/yy)***:** |
|---|---|
| ☐ Recovered<br>☐ Recovered with sequalae<br>☐ Continuing<br>☐ Resulted in Death<br>☐ Unknown | **13. Date patient died** ____/____/_____ *(dd/mm/yy)***:** |

| **14. Signature Person completing report:** | **Name:** | **Date:** |
|---|---|---|

| **15. Signature Principal Investigator:** | **Name:** | **Date:** |
|---|---|---|
